# Master Regulators of Oncogenic *KRAS* Response in Pancreatic Cancer: An Integrative Network Biology Analysis

**DOI:** 10.1371/journal.pmed.1002223

**Published:** 2017-01-31

**Authors:** Shivan Sivakumar, Ines de Santiago, Leon Chlon, Florian Markowetz

**Affiliations:** 1 Cancer Research UK Cambridge Institute, University of Cambridge, Cambridge, United Kingdom; 2 CRUK & EPSRC Cancer Imaging Centre in Cambridge and Manchester, Cambridge and Manchester, United Kingdom; MSKCC, UNITED STATES

## Abstract

**Background:**

*KRAS* is the most frequently mutated gene in pancreatic ductal adenocarcinoma (PDAC), but the mechanisms underlying the transcriptional response to oncogenic *KRAS* are still not fully understood. We aimed to uncover transcription factors that regulate the transcriptional response of oncogenic *KRAS* in pancreatic cancer and to understand their clinical relevance.

**Methods and Findings:**

We applied a well-established network biology approach (master regulator analysis) to combine a transcriptional signature for oncogenic *KRAS* derived from a murine isogenic cell line with a coexpression network derived by integrating 560 human pancreatic cancer cases across seven studies. The datasets included the ICGC cohort (*n* = 242), the TCGA cohort (*n* = 178), and five smaller studies (*n* = 17, 25, 26, 36, and 36). 55 transcription factors were coexpressed with a significant number of genes in the transcriptional signature (gene set enrichment analysis [GSEA] *p* < 0.01). Community detection in the coexpression network identified 27 of the 55 transcription factors contributing to three major biological processes: Notch pathway, down-regulated Hedgehog/Wnt pathway, and cell cycle. The activities of these processes define three distinct subtypes of PDAC, which demonstrate differences in survival and mutational load as well as stromal and immune cell composition. The Hedgehog subgroup showed worst survival (hazard ratio 1.73, 95% CI 1.1 to 2.72, coxPH test *p* = 0.018) and the Notch subgroup the best (hazard ratio 0.62, 95% CI 0.42 to 0.93, coxPH test *p* = 0.019). The cell cycle subtype showed highest mutational burden (ANOVA *p* < 0.01) and the smallest amount of stromal admixture (ANOVA *p* < 2.2e–16). This study is limited by the information provided in published datasets, not all of which provide mutational profiles, survival data, or the specifics of treatment history.

**Conclusions:**

Our results characterize the regulatory mechanisms underlying the transcriptional response to oncogenic *KRAS* and provide a framework to develop strategies for specific subtypes of this disease using current therapeutics and by identifying targets for new groups.

## Abstract 2

Florian Markowetz and colleagues study transcriptional mechanisms influenced by mutated KRAS, which is common in pancreatic ductal adenocarcinomas, and possible implications for disease characteristics and prognosis.

## Author Summary

**Why Was This Study Done?:**

- Outcomes for patients diagnosed with pancreatic cancer are very poor because surgical approaches plus other current treatments are often inadequate to treat this disease.
- Previous efforts have been made to subtype the disease in an effort to identify more clinically relevant groups for tailored treatment.
- To improve on these “landscape” studies, we focussed on transcriptional changes induced by *KRAS* mutations to understand perturbed pathways and their effects on patients.

**What Did the Researchers Do and Find?:**

- We created a transcriptional signature of oncogenic *KRAS* in an isogenic mouse ductal cell line.
- We then combined this signature with a coexpression network derived from a large collection of pancreatic cancer cases and used a well-validated algorithm to identify the transcription factors (so-called master regulators) responsible for the signature.
- The master regulators clustered into three distinct biological groups (Notch, cell cycle, and Hedgehog) characterised by significant differences in clinical survival and mutational load as well as immune cell and stromal infiltration.

**What Do These Findings Mean?:**

- Our results provide evidence that distinct modes of transcriptional reprogramming occur following *KRAS*-mediated transformation
- This improved understanding of pancreatic cancer biology may provide novel prognostic and therapeutic opportunities to counter this devastating disease.

## Introduction

Pancreatic ductal adenocarcinoma (PDAC) is the most lethal human malignancy, with a 5-y survival of 4% [1]. There are very few treatment options, with the only chance of a cure being surgical resection if the tumour is detected at an early, confined stage. Chemotherapeutic options are used in the palliative setting but have toxic side effects and do not extend survival for more than a few months. Pancreatic cancers display vast intratumoural heterogeneity with respect to their mutational profiles [2], but more than 90% of cases have a mutation in the *KRAS* oncogene, which almost exclusively is located in codon 12 [3].

Other highly recurring mutations in PDAC include *INK4/ARF*, *P53*, and *SMAD4* [4]. Recent studies [4–6] on the genomic landscape of PDAC have identified alterations in genes related to chromatin remodeling, DNA damage repair, and axon guidance, as well as focal amplifications in druggable genes—including *ERBB2*, *MET*, and *FGFR1—*in a small subset of patients. Several recent studies [7–9] have described the transcriptomic landscape of PDAC and identified different subtypes with a link to survival.

Here, we took advantage of seven existing transcriptomic studies containing in total 560 samples to explore *KRAS* biology (see S1 Computational Analysis). The role of *KRAS* in PDAC initiation is well known [10], and cancer growth and survival often directly depend on it [11]. However, the mechanisms by which *KRAS* contributes to PDAC progression, in particular its interactions with downstream pathways, have not been equally well characterised [12]. The aims of our study were to identify transcription factors determining the transcriptional response to oncogenic *KRAS* and to explore what impact their activity has on the development of the disease and patient outcome.

## Methods

This study did not have a protocol or prospective analysis plan. To achieve the first aim, we used master regulator analysis, a well-established network biology strategy [13–16], which combines a transcriptional signature with a coexpression network to identify key transcription factors. For the second aim, we used clustering techniques to identify patient subtypes based on transcription factor activities and characterised these subtypes by survival analysis, integration of mutation data, and methods that infer immune activity from gene expression profiles. For clarity, the Methods description is split into three main sections: defining a transcriptional signature for oncogenic *KRAS*, identification of master regulators of *KRAS* response, and characterisation of PDAC subtypes. All code and scripts to reproduce our analysis are available as annotated documents as part of the supplementary information (S1 Computational Analysis, S2 Computational Analysis).

### 1. Defining a Transcriptional Signature for Oncogenic *KRAS*

Here, we describe how we derived the gene expression signature of oncogenic *KRAS*, which provides the focus point of our study.

#### Murine ductal cell line

KF508 is a murine ductal pancreatic cell line we received from the Tuveson lab. The cells were isolated from a genotype Kras^LSL-G12D^ mouse according to a published protocol [17]. Briefly, the pancreas of a KRAS^LSL-G12D^ mouse was dissected, minced, and digested at 37°C in Hank’s balanced salt solution (HBSS) (Hank’s balanced salt solution + 5 mmol/L glucose + 0.05 mmol/L CaCl2) containing 2 mg/mL type V collagenase (Sigma, St. Louis, Missouri). After 15 min, the digested material was filtered through a 105 μm nylon mesh (Small Parts, Inc.). Fragments trapped on the mesh were digested further in 0.05% trypsin–0.53 mmol/L ethylenediaminetetraacetic acid (Life Technologies) for 2 min. Proteases were inactivated by addition of DMEM/F12 (Invitrogen) supplemented with 10% fetal bovine serum. The tissue was washed three times in HBSS to remove collagenase completely. The ductal fragments were plated on 2.31 mg/mL rat tail collagen type I (BD Biosciences) precoated plastic dishes for growth in monolayer. It is noteworthy that the cells were spontaneously immortalized—i.e., they continued to proliferate after a few passages without any additional changes to promote immortalisation. The cells were transferred to plastic after five passages. The mouse is very well characterised in the literature [10, 18, 19]. The cell line was generated from a mouse with C57Bl6/129 background of unknown age and sex, and a positive E-cadherin expression was used to validate epithelial origin (2016 email from Dr. Kris Frese to Shivan Sivakumar, unreferenced). KF508 cells contain the mutant version of the *Kras* allele (G12D) preceded by a Lox-Stop-Lox cassette inhibiting transcription. Infection with Cre-expressing adenovirus (adeno-cre) deletes the floxed stop sequence, permitting Cre-mediated recombination and expression of oncogenic *Kras*^G12D^, whilst adenovirus-mock was used as a negative control. For adenoviral infections, cells were transduced with 1x10^7^ PFU adenoviral-mock or adenoviral-cre per mL of media (virus was obtained from the University of Iowa).

#### Media to propagate ductal cells

The ductal cell medium was adapted from the Schreiber protocol. A bottle of DMEM/F12 media was heated to 37°C for 30 min. The media was supplemented with 1.22mg/ml nicotinamide (Sigma), 5 mg/ml glucose (Sigma), 5% ITS + cell culture supplement (VWR), 5% Nu-Serum IV (VWR), and 20 ng/ml Epidermal Growth Factor (Peprotech). This mixture was subsequently filter sterilized through a 0.22 μM filter. To check expression of oncogenic *Kras*, the Ras GTPase activation kit from Millipore was used.

#### Gene expression profiling

The microarray experiment was performed by the genomics core facility in the CRUK Cambridge Institute. Briefly, total RNA was extracted from six sets of mock- and cre-treated ductal cells using the Qiagen RNeasy kit. The RNA was labelled and hybridized. This experiment was carried out on two Illumina Mousev2 BeadChips using 24 arrays (1 sample per BeadChip). These arrays interrogate 46,235 randomly distributed bead types, and in this experiment there was a mean of 21 beads per bead type (standard deviation of 6). In total, there are 20,562 genes being interrogated in this experiment, with 8,472 genes being interrogated by more than one bead type and 12,014 bead types not being assigned to a gene symbol. Differential expression analysis between the two groups (“mock” versus “cre”) was carried out using the limma Bioconductor package [20]. Mouse Ensembl IDs were mapped to human Ensembl IDs in Ensembl 50, using biomart (http://www.ensembl.org/biomart).

### 2. Identification of Master Regulators

Here, we describe the data and methods to build a coexpression network for PDAC and how it is used to identify the transcription factors determining the *KRAS* signature, the so-called master regulators.

#### Collection of 560 gene expression profiles from 7 independent studies

Raw gene expression data files were obtained from Gene Expression Omnibus (GEO) for a total of five independent, pancreatic-related studies [8,18–22]. GEO accession numbers for these five studies are GSE17891 (*n* = 26), GSE15471 (*n* = 36), GSE16515 (*n* = 36), GSE32676 (*n* = 25), and GSE2109 (*n* = 17). All five downloaded datasets were generated on the Affymetrix GeneChip^®^ Human Genome U133 Plus 2.0 array. The ICGC microarray gene expression data (*n* = 242) plus clinical metadata and mutational profiles were directly obtained from the authors of Bailey et al. 2016 [9]. TCGA RNA-seq data [22] (*n* = 178) was obtained with permission from the Cancer Genomics Hub (https://cghub.ucsc.edu). Clinical metadata for the TCGA cohort was downloaded via cBioPortal [21].

#### Processing of gene expression datasets

QC analysis was performed using affy and affyPLM [22]. R packages and normalization was performed with RMA (robust multichip averaging) method [23]. Batch effects in the ICGC cohort were removed by applying the ComBat algorithm [24]. TCGA RNA-seq data was transformed with the variance-stabilizing transformation method [25].

#### Identification of core disease processes

Network inference was done using the RTN [13, 14] package and coexpression analysis using partial correlations. We applied the pcor.shrink function from the corpcor [26] with default parameters. The significance of each partial correlation was computed using the fdrtool R package [13,14,27,28]. Master regulators were identified with the msviper functionality of the viper package [29], and community detection was performed by using the fastgreedy.community function in the igraph R package [30].

### 3. Characterisation of PDAC Subtypes

The core disease processes define three subtypes, which we comprehensively characterized.

#### Pathway analysis

Pathway and gene ontologies enrichment analyses were performed in R by hypergeometric test or gene set enrichment analysis as indicated. Kyoto Encyclopedia of Genes and Genomes (KEGG) and two GO gene set collections obtained from KEGG.db and GO.db databases in R were used.

#### Characterization of PDAC subtypes

In order to detect the similarity between tumour samples based on the activity of the 27 master regulators, we used viper [29] to perform a single-sample analysis and compute a matrix with activity scores for each one of the 27 regulators. The distance between the tumour samples was computed with the signature distance method with the default parameters in viper [29]. PDAC subgroups were identified by applying hierarchical clustering with agglomerative average linkage.

#### Survival analysis

Survival analysis was performed using the Kaplan–Meier estimator, comparison among groups was analysed using log-rank tests, and multivariate analysis was performed using the Cox proportional hazard model. For the ICGC cohort, we adjusted the survival differences for age, gender, and tumour stage covariates. For the TCGA cohort, we modelled the interaction between tumour subtype (group label) and a binary (yes/no) targeted therapy indicator, adjusting for age, gender, tumour stage, and radiation treatment indicator. The two binary treatment indicators considered (targeted therapy and radiation treatment) were retrieved from “targeted_molecular_therapy” and “radiation_treatment_adjuvant” labels, respectively, of the associated TCGA clinical metadata. These indicators refer to whether the patient had adjuvant and/or postoperative pharmaceutical or radiation therapy. More specific information about the treatment regime was not possible to obtain.

#### Analysis of mutational data

We used the previously identified landscape [9] of somatic mutations and copy number alterations in the ICGC cohort to investigate the frequency of samples with mutations in 39 key genes in pancreatic cancer [5]. Somatic mutations were filtered out to include only alterations with high and moderate impact on the basis of their predicted effect on the protein [31]. Copy number alterations were restricted to losses of at least one copy and to amplifications of more than five copies.

#### Analysis of leukocyte infiltration and stromal content

ESTIMATE [32] was used to gauge the degree of leukocyte infiltration and stromal content within the tumour microenvironment for each of the three derived pancreatic cancer subtypes. The Wilcoxon rank sum test was used to elucidate whether the computed immune and stromal infiltrates were significantly different across the subtypes.

We compared the samples by their enrichment for the Hedgehog, Notch, and cell cycle pathways using single-sample gene set enrichment analysis (ssGSEA).We used the R implementation of ssGSEA: GSVA (gene set variation analysis) with default parameters. Three gene set collections were downloaded from the Broad Institute Molecular Signature Database (MSigDB): the curated KEGG, BIOCARTA v5.0, and C5 Gene Ontology collections [33]. These were aggregated and filtered in order to build a single collection containing only immunological pathways. For each sample, Hedgehog, Notch and cell cycle enrichment was determined by running ssGSEA, using a curated gene set for each pathway. We furthermore calculated the enrichment for each immunological pathway in the collection by running ssGSEA across all the samples. First-order partial correlation coefficients between Hedgehog/Notch and the immunological pathways were computed using the R package “ppcor” to correct for the strong positive correlation between Hedgehog and Notch (see S2 Computational Analysis). *p*-values were multiplied by the number of correlations to correct for multiple testing-associated type I errors. Associations between Hedgehog and Notch and an immunological pathway were deemed “significantly associated” if one or the other had *p* < 0.05.

#### Gene expression deconvolution

We used the deconvolution tool CIBERSORT to estimate the fraction of immune cells in the bulk tumour using gene expression data [34]. CIBERSORT uses the expression profiles of purified samples to derive a matrix of 22 immune cell type signatures (LM22), which can be used to elucidate the immune microenvironment composition of bulk tumour samples [34]. We preprocessed the discovery and validation datasets according to CIBERSORT requirements. For the microarray data, probes mapping to more than one gene were removed from the analysis. For genes mapping to more than one probe, we selected the probe with the highest variance value across the sample space.

Data were quantile normalised and CIBERSORT was run with 1,000 permutations. A threshold of *p* < 0.05 defined a successful CIBERSORT deconvolution. First-order partial correlations were used to evaluate whether any significant associations existed between any of the leukocyte cell types and the Hedgehog/Notch pathways. The ICGC and TCGA cohorts were treated as discovery and validation cohorts, respectively. ICGC cohort *p*-values were adjusted for multiple testing by multiplying by the number of partial correlation tests. A threshold of *p* < 0.05 defined significance in both cohorts. The t-statistics of the partial correlation between Hedgehog/Notch and the leukocyte fractions was used as a comparison metric (see S2 Computational Analysis).

Immune checkpoint gene expression, such as those for PD-1 and PD-L1, were tested for association with both the Hedgehog and Notch pathways through the use of first-order partial correlation analysis. 44 pancreatic cancer cell lines were downloaded from the Broad Institute Cancer Cell Line Encyclopedia. These were classified into “Hedgehog,” “cell cycle,” or “Notch” by means of the nearest shrunken centroids method implemented via the R package “pamr.” This was then verified by comparing the subtype classification with each cell line’s ssGSEA-derived enrichment score for the Hedgehog, cell cycle, and Notch pathways.

## Results

### Network Analysis Identifies *KRAS*-Specific Master Regulators

**Fig 1:**
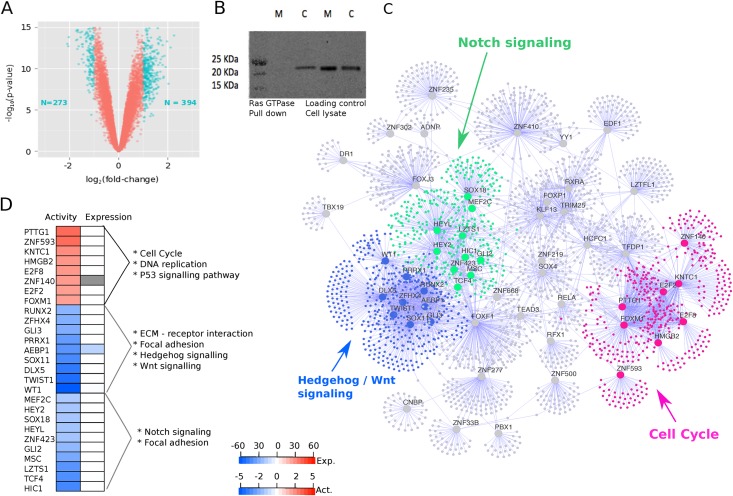
Oncogenic *KRAS* is regulated by three groups of master regulators. **A.** Volcano plot showing the magnitude of the differential gene expression between murine mock ductal cells and murine cre ductal cells (with activated oncogenic *Kras*). Each dot represents one probe with detectable expression in both conditions. The coloured dots mark the threshold (*p* < 0.05 and log2 fold-change > 1) for defining a gene as differentially expressed. **B.** Ras GTPase assay shows increased GTPase activity in cre cells blotted with pan-Ras antibody (M, mock; c, cre). **C.** Visual representation of master regulators (MRs) identified with msVIPER analysis (*p* < 0.01). The nodes in the networks represent the 55 master regulators (large dots) and the corresponding inferred targets (smaller dots). The edges in the network represent the regulatory relationship between regulators and the inferred targets. The colours highlight the community structure of the network identified via greedy optimization of modularity. The three groups of nodes correspond to a total of 27 master regulators and represent three distinct disease processes enriched for cell cycle (pink), Hedgehog/Wnt signalling (blue), and Notch signalling (green) pathways. **D.** For the 27 MRs in the three core processes, the heat map shows their activity (first column) and differential expression in the *KRAS* signature (second column) as obtained by Virtual Inference of Protein-activity by Enriched Regulon (VIPER) analysis. “Expression” refers to the differential expression value after *KRAS* induction (cell line experiment). The colour code of in the heat map corresponds to the t-statistic value obtained after limma differential expression analysis, with blue representing down-regulated genes and red representing up-regulated genes after *KRAS* activation. “Activity” refers to the differential protein activity value after *KRAS* induction with red or blue representing activation or inactivation, respectively. The protein activity score is quantitatively inferred by the aREA algorithm in VIPER by systematically analysing expression of genes coexpressed with the transcription factor (TF).

A *KRAS*-specific transcriptional signature enables us to understand the regulatory patterns induced in the presence of this mutation through downstream network analysis. To facilitate this analysis, we first derived a transcriptional signature of oncogenic *KRAS* by using a previously reported murine pancreatic ductal cell line with an inducible Lox-Stop-Lox (LSL) cassette in front of the *KRAS*^G12D^ oncogene to regulate transcription [35]. Comparing the transcriptomes of induced *KRAS*-on cells with *KRAS*-off control population in six replicates, we identified 667 differentially expressed probe sets (Fig 1A). Using gene set enrichment analysis, we found that the *KRAS* signature was enriched for many transcript changes in the pathways associated with the MAPK pathway, regulation of cell growth, and regulation of the actin cytoskeleton, which is in line with established functions of *KRAS* (S1 Table). Given that these cell lines are derived from murine samples, genes in the signature were mapped to their equivalent human orthologue where possible and removed from the signature if no orthologue exists.

To study which transcription factors (TFs) act as master regulators for the observed transcriptional changes, we used 560 gene expression profiles collected from seven independent studies [8, 9, 36–40] to infer a coexpression network between transcription factors and their potential targets. Details of datasets are described in the Methods section. We used different safeguards against biases potentially incurred during data integration. Every dataset was normalized independently and corrected for batch-effects if it contained sample subgroups (S2 Fig). We then used a shrinkage estimate of partial correlations [26] to infer a network between regulators and their targets in each dataset. The shrinkage estimate automatically adapts to differences in sample size and ensures reliable results across studies. A significant partial correlation corresponds to an edge in the network, and the set of all inferred targets of a regulator is called its “regulon.” We confirmed that the regulons were well conserved between the seven networks (S3 Fig). We then converted the *p*-values of all edges into Z-scores and combined them into a single, integrated network using Stouffer’s meta-analysis score [28], which weights the significance of an edge by the size of each study to ensure that larger cohorts contribute more to the integrated network. Thus, the final integrated network consists of edges showing consistent results across all seven studies.

In the next step, we superimposed the transcriptional *KRAS* signature onto the regulatory network using the VIPER (Virtual Inference of Protein-activity by Enriched Regulon analysis) algorithm [5, 9, 13, 14, 29, 40]. VIPER tests for significant overlap between genes in the transcriptional signature and genes in each regulon. 55 regulons were significantly enriched for signature genes, and we called the corresponding TFs “master regulators” (MRs) of activated oncogenic *KRAS* (S4 Fig). Several MRs have been previously implicated in PDAC, such as GLI3, AEBP1, and CASP5, while some of the MRs have not been associated with PDAC before, such as TCF21, TWIST1, and FOXF2.

To identify the core biological processes represented by these master regulators, we used a community detection algorithm on the transcriptional network and identified Notch signaling, Hedgehog/Wnt signalling, and cell cycle as close clusters of 27 of the 55 MRs (S5 Fig). Eight of the 27 MRs had an overall activation in protein gene up-regulation activity, and the other 19 had repressed protein activity (Fig 1D). The Notch and Hedgehog/Wnt clusters cluster closer together in the network, whereas the cell cycle MRs form a distinct group (Fig 1C).

### *KRAS* Master Regulators Define Three Core Regulatory Processes

**Fig 2:**
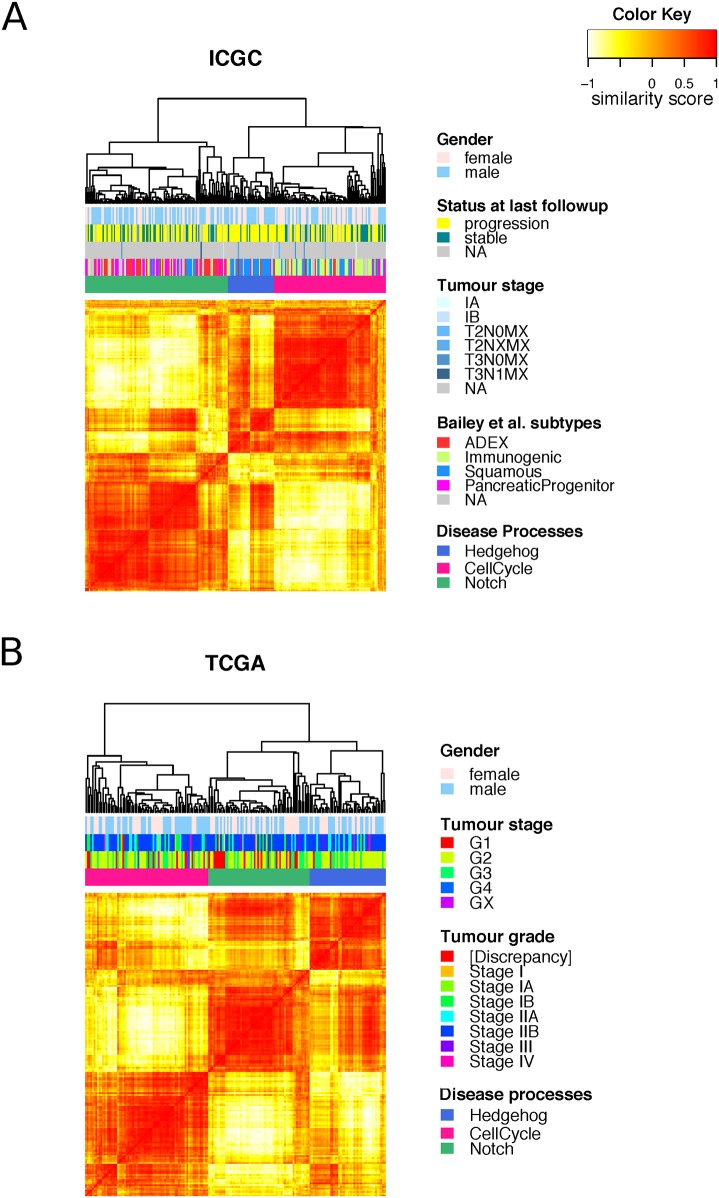
Clustering of master regulators into different functional groups. Heat maps showing the similarity between the samples in the **A**. ICGC and **B**. TCGA cohorts as measured by “signature distance” between the MRs activity profiles [27]. Unsupervised analysis identified three classes of tumours with differential activities of the three identified disease processes: cell cycle (pink), Hedgehog/Wnt (blue), and Notch (green).

Based on the expression of genes within each regulon, we inferred the activity of the MRs in each patient sample (S1 Computational Analysis). These activity scores clearly defined three disease processes (Fig 2). This result indicates that even though PDAC has *KRAS* as a dominant oncogene driver, it develops along one of three distinct paths, which can be identified by their underlying biological pathways.

The murine cell line we used to derive the transcriptional signature harboured a G12D mutation, whereas patients in the TCGA and ICGC cohorts also showed a variety of *KRAS* mutation types. However, we did not find any of our subtypes biased towards one particular *KRAS* mutation type (S7 Fig).

When comparing our disease regulatory processes to the subtypes identified by Bailey et al. [9], we found that the Hedgehog/Wnt process is overrepresented by samples from the squamous subtype (p_hyper_ = 1.1x10^−11^), the cell cycle process by samples from the immunogenic subtype (p_hyper_ = 7.8x10^−12^), and the Notch by samples from the ADEX (*p*-value = 8.2x10^−8^) and pancreatic progenitor (p_hyper_ = 6.1x10^−8^) subtypes (S6 Fig).

### MR Regulatory Processes Show Different Survival and Mutational Load

In all further analyses, we focused on the two biggest studies in our dataset collection and used the ICGC cohort [9] (*n* = 242) as a discovery set and the TCGA cohort [40] (*n* = 178) as an independent validation set. The cohorts are comparable and patients all had their pancreatic cancers resected at early stage disease (typically stage I/II).

**Fig 3:**
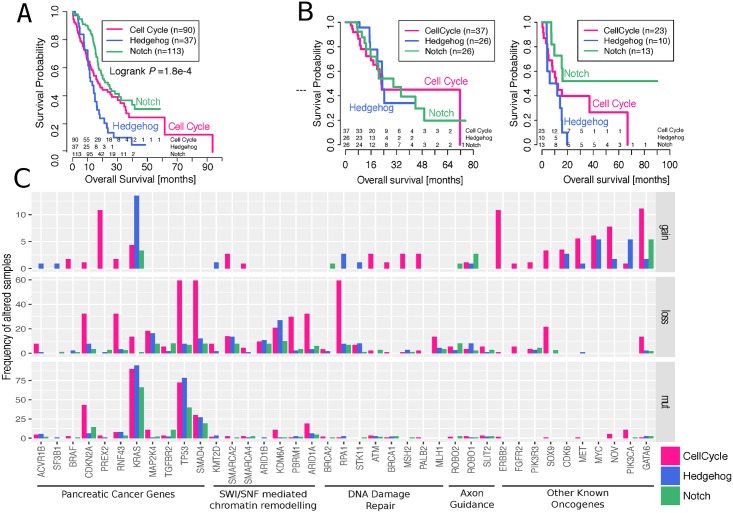
Differences in survival and mutational burden between subtypes. **A.** Kaplan–Meier survival curves of the different tumour subgroups using the ICGC cohort. Numbers of subjects at risk at the start of each time interval are shown above the *x*-axis. The groups overall showed significant survival differences (logrank *p*-value = 1.8e–4). More specifically, Hedeghog/Wnt group HR = 1.73, 95% CI 1.1 to 2.72, coxPH test *p*-value = 0.018; Notch group HR = 0.62, 95% CI 0.42 to 0.93, coxPH test *p*-value = 0.019; when compared to the cell cycle group and after correcting for gender, age, and tumour stage. **B.** Kaplan–Meier survival curves of the different tumour subgroups using the TCGA cohort for subsets of individuals that did or did not receive adjuvant targeted therapy treatment. Numbers of subjects at risk at the start of each time interval are shown above the *x*-axis. Hedeghog/Wnt group HR = 4.12, 95% CI 1.2 to 13.8, coxPH test *p*-value = 0.02; when compared to the cell cycle group and after correcting for gender, age, tumour stage, and radiation therapy indicator. **C.** Mutations in key genes and pathways in pancreatic cancer. The upper and middle panels show the frequency of altered samples by copy number changes (gains refer to amplifications of >5 copies); the bottom panel shows the frequency of altered samples by nonsilent single nucleotide variants, small insertions, or deletions with moderate-to-high biological effect.

To quantify the clinical importance of the different regulatory processes, we compared them to survival data (clinical features of both cohorts are summarized in S2 Table). In discovery and validation cohorts, the Hedgehog/Wnt group has a very poor prognosis, while the Notch group has the best prognosis (Fig 3A) (Cox proportional hazards regression model, Hedeghog/Wnt group HR = 1.73, 95% CI 1.1 to 2.72, *p*-value = 0.018; Notch group HR = 0.62, 95% CI 0.42 to 0.93, *p*-value = 0.019; when compared to the cell cycle group and after correcting for gender, age, and tumour stage). Our findings agree with previous observations linking the repression of the Hedgehog pathway to more aggressive pancreatic cancers [9, 41, 42], and this particular subtype also overlapped with the poor prognostic group found in Bailey et al. [9].

For consistency with the ICGC cohort, we substratified the TCGA dataset into treated and untreated samples. The untreated cohort demonstrated identical survival stratification to our discovery cohort, with the Notch group exhibiting favourable outcome and Hedgehog/Wnt exhibiting the worst. Although these results reciprocated the findings in the discovery cohort, the *p*-value was not significant at a threshold of 0.05, which is in part due to the small sample size and the low number of events.

Using a multivariate Cox proportional hazards regression analysis, we noticed that different pharmaceutical treatments had an effect on survival rates (ANOVA *p*-value = 0.04; after adjusting for gender, age, and tumour stage). We split the cohorts into “treated” and “nontreated” based on the treatment indicator in the TCGA data and observed that the prognosis for the Hedgehog/Wnt group was worse only if the patients are not treated (HR = 4.12 compared to cell cycle group; 95% CI 1.2 to 13.8, coxPH test *p*-value = 0.02; after correcting for gender, age, tumour stage, and radiation therapy indicator). In the vast majority of cases, gemcitabine, a known chemotherapy agent, was listed as listed as the “drug name” by itself (57%) or in combination with another chemotherapy drug (e.g. oxaliplatin, irinotecan, Abraxane) or one of the other listed drugs (39%), suggesting that chemotherapy was the basis of the “targeted therapy” indicator. However, it was not possible to obtain information about the specifics of the treatment regime, and we also noted that 12 cases annotated as “not treated” in the TCGA data were also listed as receivers of gemcitabine. Thus, our analyses are exploratory and no final conclusions are possible, yet there is some evidence that targeted therapeutics used in the Hedgehog/Wnt group show a pharmacological benefit to this group with a dismal prognosis.

We next investigated the molecular basis for these survival differences and compared the mutational patterns between the three subtypes (Fig 3C). We found that the cell cycle process had a significantly (ANOVA *p*-value < 0.01) higher mutational burden than the Hedgehog/Wnt and Notch processes—with an average of 9.4% of altered samples in the cell cycle group, compared to 4.6% and 3.2% in the Hedgehog/Wnt and Notch groups, respectively—when considering a panel of 39 key pancreatic cancer genes and pathways [5]. The same pattern was observed in the data from the TCGA cohort (S8 Fig).

### MR Regulatory Processes Show Different Immune Activity

To understand how well the patient subtypes were represented in experimental model systems, we used the Broad Institute’s cell line resource [43] and found that all the cell lines shared the characteristics of the cell cycle process but not of the other two processes (S9 Fig). As cell lines are artificially homogeneous cell populations, we hypothesized that Hedgehog and Notch activity are determined by the tumour’s interaction with its microenvironment.

**Fig 4:**
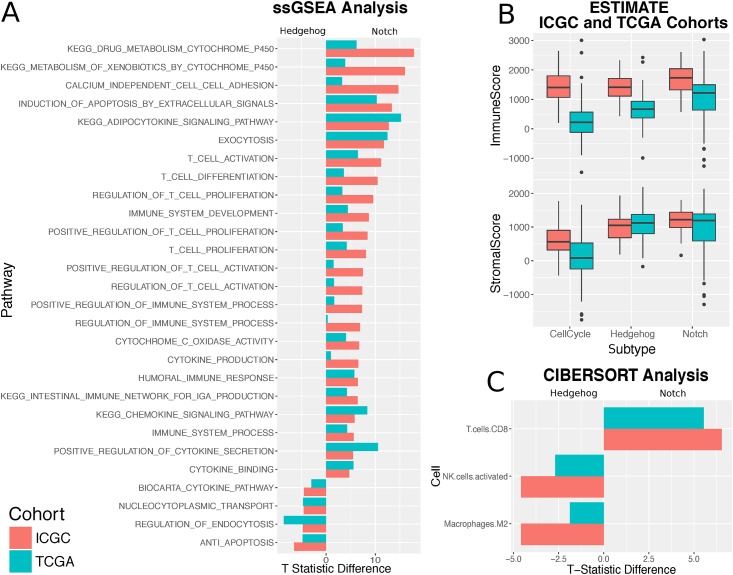
Subtypes show different immune activity. **A.** Bar plot showing the Pearson partial correlation t-statistic difference between Hedgehog and Notch for ssGSEA pathway enrichment scores significantly associated with one subtype or the other. **B.** Boxplots from the ESTIMATE analysis showing the variation in the stromal and immune content between the Hedgehog, Notch, and cell cycle subgroups for both the ICGC and TCGA cohorts. **C.** Bar plot showing the difference in Pearson partial correlation coefficient difference between Hedgehog and Notch for estimated CIBERSORT leukocyte cell fractions significantly associated with one subtype or the other. A negative difference highlights strong association with Hedgehog, whereas a positive value indicates a strong association with Notch.

We thus focused on differences in the tumour microenvironment to explain the differences between regulatory processes and used ESTIMATE [32] to infer stromal and immune cell admixture from gene expression data. The samples in the Hedgehog and Notch processes showed a significantly higher stromal infiltration compared to the cell cycle subtype (Fig 4B) on both the discovery and validation cohort. All three subgroups showed significantly varied levels of immune cell enrichment, with Notch being the most immunogenic and cell cycle being the least. Although Hedgehog and cell cycle samples demonstrate considerable stromal infiltration, Hedgehog is significantly less immunogenic in both cohorts (ICGC: *p* = 1.7e–05, TCGA: *p* = 7.8e–08; Wilcoxon rank sum test). By contrast, the difference in stromal content between the Hedgehog and Notch classes is considerably subtler (ICGC: *p* = 0.0016; TCGA *p* = 0.97; Wilcoxon rank sum test).

We used complementary strategies to explore the immune content of the three processes. First, we aggregated a collection of 77 immunological pathways from MSigDB. We found significant positive associations between Notch activity and 22 pathways. Of these were enrichment for T cell–related pathways, such as those pertaining to T cell activation, proliferation, and differentiation (Fig 4A). General immune system pathways, including the adaptive immune response and the positive regulation of the immune response, were similarly far more associated with Notch than Hedgehog. No significant associations were observed between T cell–related pathways and Hedgehog activity enrichment across both datasets (S10 Fig).

We then focused on dissecting the heterogeneity of leukocyte populations in the tumour samples using a leukocyte deconvolution method [32]. Both methods use a panel of cell type–specific signatures to identify which cell types are present in a mixed gene expression profile (see Methods). Results between the ICGC and TCGA cohorts correlated well, with both demonstrating the significant prevalence of infiltrating CD8+ T cells in Notch pathway–dominated samples (Fig 4C) and a dominant presence of M2 macrophages in samples exhibiting strong Hedgehog signalling.

Finally, we correlated Notch and Hedgehog activity with the expression of known immune therapy targets [44]: programmed cell death protein 1 (PD-1), programmed death-ligand 1 (PD-L1), cytotoxic T lymphocyte–associated protein 4 (CTLA4), TIGIT, and LAG3. Across both the discovery and validation cohorts, CTLA4, PD-1, and TIGIT showed significant positive correlations with Notch activity, whereas no significant associations were observed with Hedgehog activity (S10 Fig).

## Discussion

The first aim of our study was to identify transcription factors determining the transcriptional response to oncogenic *KRAS*. To address this aim, we used master regulator analysis to combine a murine gene expression signature of oncogenic *KRAS* with a coexpression network integrating data from 560 patients. We found that the *KRAS*-specific master regulators represented three main biological processes: Notch, repressed Hedgehog/Wnt, and the cell cycle. The second aim was to explore the extent to which activity of these biological processes had on the development of the disease and patient outcome. We demonstrated that the functional groupings of the transcription factors represented distinct clinical entities of PDAC, with differences in survival, mutational load, and immune activity. These three patient subtypes represent three routes of PDAC development, characterized by three different transcriptional programs.

Our study has several limitations. Although the number of samples studied here is large compared to those of other publications in PDAC, the size of the patient cohorts prevents strong conclusions about effect sizes and clinical impact. By reanalysing mainly published data, our study is constrained by the quantity and quality of the information provided in these studies. For example, mutational profiles were not available for all patients and the clinical information is often incomplete, which impedes the assessment of treatment effects. In addition, we did not have access to tissue sections for the analysed genomic profiles that could have been used to validate hypotheses about immune activity in the subtypes.

The groups we identified in general agreed with previous patient stratifications, with one surprising exception: the cell cycle group overlapped significantly with Bailey et al.’s immunogenic subtype [9], even though in our reanalysis of the data, these patients show little evidence for immune activity. This indicates that more work needs to be done to consolidate different stratification schemes into consensus subtypes of pancreatic cancer.

We found some evidence demonstrating the potential clinical importance of the groups we identified. Using survival data from two independent cohorts, we demonstrated that the Hedgehog process has the worst prognosis and the Notch process has the best prognosis. We demonstrated that the cell cycle process has the largest mutational burden of all the regulatory processes and has a smaller stromal composition. At the same time, the Notch and Hedgehog/Wnt groups showed substantial stromal contributions. We characterized stromal content and found M2 macrophages enriched in the Hedgehog/Wnt process and CD8+ T cells in the Notch process. Furthermore, analysis of checkpoint markers showed that the T cells collated with CTLA, PD-1, and TIGIT. This indicates that the Notch subtype is potentially amenable to immunotherapy. The Hedgehog/Wnt group, even though it has the worst prognosis, is potentially amenable to newer targeted therapies.

In summary, these examples show how the transcriptional signatures of *KRAS*-specific master regulators could be used in the clinic to stratify patients and guide therapeutic decisions.

## Supporting Information

S1 Fig Overall schematic of the project. (EPS)

S2 Fig Preprocessing of GEO microarray data. (A) RLE and NUSE distributions for the seven cohorts of PDAC gene expression profiling used. (B) Distribution of probe intensity scores before and after RMA normalisation.
(EPS)

S3 Fig Network comparisons. (A) Edge weight correlation between pairs of networks. For each regulon, Pearson correlation coefficients were computed by comparing the edge z-scores between pairs of networks. The box plots display the distribution of regulon Pearson correlation coefficients when considering all edges (orange) or when considering only the edges with significant FDR in both networks (q-value < 0.05) (blue). The correlation between edge weights increases when considering only significant edges. (B) Regulon overlap between pairs of networks. Regulon overlap was computed by considering the edges with significant FDR (q-value < 0.05) and the *p*-value for the degree of overlap calculated by a hypergeometric test. (C) Agreement of *p*-values obtained by msVIPER analysis for the top 55 master regulators in all seven individual networks and in the combined fused Network (comb). Networks with higher number of samples (ICGC and TCGA cohorts) are in better agreement with the combined network result.–log10 (*p*-values) are coloured from the smallest *p*-values (red) to the largest (blue). White cells in the heat map correspond to missing values; these exist for master regulators with no inferred edges at a FDR < 0.05. (D) Agreement of normalized enrichment scores (NES) obtained by msVIPER analysis for the top 55 master regulators in all seven individual networks and in the combined fused network (comb). Networks with a higher number of samples (ICGC and TCGA cohorts) are in better agreement with the combined network result. Negative NES scores are coloured in blue and positive NES scores in red. White cells in the heat map correspond to missing values; these exist for master regulators with no inferred edges at a FDR < 0.05.
(EPS)

S4 Fig Identification of master regulators using msVIPER. msVIPER plot showing the projection of the negative (repressed, shown in blue colour) and positive (activated, shown in red colour) targets for each TF, as inferred by RTN and partial-correlation analysis when reverse engineering the regulatory network (vertical lines resembling a barcode), on the KRAS gene expression signature. The KRAS signature is represented in the *x*-axis, where the genes were rank-sorted based on the t-statistic for the differential gene expression test (limma analysis) from the one most down-regulated to the one most up-regulated in the “cre” condition compared to “mock” (i.e., after oncogenic KRAS activation). The two-column heat map displayed on the right side of the figure shows the inferred differential activity (first column) and differential expression (second column), with the rank of the displayed genes in the KRAS signature (shown all the way to the right).
(EPS)

S5 Fig KEGG pathway enrichment analysis for the three identified disease processes. (A) Bar plot showing all enriched categories in the KEGG pathway database at a FDR-adjusted *p*-value < 0.01. (B) The plot shows all enriched categories at FDR adjusted *p*-value < 0.01 per group. The total number of identified genes in each group is provided as numbers in parentheses in the *x*-axis. The dots in the plot are colour-coded based on their corresponding FDR-adjusted *p*-values. The size of the dots corresponds to the "gene ratio," which corresponds to the proportion of genes from each group in each category of KEGG pathways.
(EPS)

S6 Fig Comparison of disease regulatory processes identified in this study with the tumour subtypes identified previously in Bailey et al. 2016. The Hedgehog/Wnt process is overrepresented by samples from the squamous subtype (*p*-value = 1.1e–11), the cell cycle process by samples from the immunogenic subtype (*p*-value = 7.8e–12) and the Notch by samples from the ADEX (*p*-value = 8.2e–8) and pancreatic progenitor (*p*-value = 6.1e-8) subtypes. The *p*-values for the degree of overlap were calculated by a hypergeometric test.
(EPS)

S7 Fig Distribution of KRAS mutation types within the three disease regulatory processes. (A) Heat maps showing the similarity between the samples in the ICGC and TCGA cohorts as measured by signature distance between the MRs’ activity profiles. Metadata (columns) show the three identified disease processes: cell cycle (pink), Hedgehog/Wnt (blue), and Notch (green) and different KRAS mutational status: wild-type (wt), G12D, G12R, G12V, and other (“other” includes A11T, G12A, G12C, G12L, G12S, G13C, G13P, GQ60GK, Q61H, and Q61R). (B) Proportion of samples from each disease regulatory process with different types of KRAS mutations. No significant enrichment of a type of KRAS mutation was identified in the ICGC (*p*-value = 0.6471) or the TCGA (*p*-value = 0.3495) cohorts. *p*-values were calculated using the Fisher exact test.
(EPS)

S8 Fig Mutations in key genes and pathways in the TCGA cohort. The upper and middle panels show the frequency of altered samples by copy number changes (GISTIC score ≥ 1 for gains, GISTIC score ≤ –1 for losses); the bottom panel shows the frequency of altered samples by nonsynonymous somatic mutations in gene coding regions.
(EPS)

S9 Fig Cell line analysis. (A) ssGSEA analysis is used to measure the enrichment of the Hedgehog/Wnt, Notch, and cell cycle pathways for 44 cell line transcriptomes downloaded from the Broad Institute’s cell line resource (https://portals.broadinstitute.org/ccle/). It is evident that all cell lines are consistently strongly enriched for cell cycle, whereas Hedgehog and Notch are down-regulated. (B) Heat map showing the expression of cell cycle, Hedgehog/Wnt, and Notch master regulators across all 44 cell line transcriptomes from the Broad Institute’s cell line resource. It can be seen that almost all master regulators pertaining to the cell cycle group are overexpressed and cluster together, whereas no overexpression is observed in the Hedgehog/Wnt and Notch master regulators.
(EPS)

S10 Fig Analysis of immunogenicity. (A) Bar plots of the partial correlation t-statistic between TIGIT, PDCD1 (PD-1), and CTLA4 with the ssGSEA enrichment scores of the Hedgehog and Notch pathways. This analysis is done for both the ICGC and TCGA cohorts. The dotted line designates the significance threshold. The significant associations are consistent across both cohorts. (B) Bar plots of the partial correlation t-statistic between the enrichment scores for a set of T cell–related pathways and the enrichment scores for the Hedgehog and Notch pathways. The dotted line designates the respective significance threshold for the ICGC and TCGA cohorts. It is evident that only Notch has a significant, positive association with the given T cell pathways across both datasets. Hedgehog, on the other hand, has no significant association with these pathways whatsoever. These findings are consistent across both datasets.
(EPS)

S1 Computational Analysis Zipped file containing a set of documentation, R code, results, and datasets to fully reproduce the results and analysis of the following results subsections of the publication: “Network analysis identifies KRAS-specific master regulators,” “KRAS master regulators define three core regulatory processes,” and “MR regulatory processes show different survival and mutational load.” Also included is a PDF output of the complied code.
(ZIP)

S2 Computational Analysis Zipped file containing a set of documentation, R code, results, and datasets to fully reproduce the results and analysis of the following results subsection of the publication: “MR regulatory processes show different immune activity.” Also included is a PDF output of the complied code.
(ZIP)

S1 STROBE Checklist A checklist of items concerning cohort information included in this publication in accordance with the *PLOS Medicine* guidelines. (DOC)

S1 Table Pathway GSEA analysis of KRAS differentially expressed genes. Obtained using HTSanalyzeR for gene set collections obtained from Biological Processes Gene Ontology, Molecular Function Gene Ontology, and KEGG GENES database. The table shows significantly enriched terms and/or pathways according to both the GSEA and hypergeometric test.
(XLS)

S2 Table Summary of clinical features in the ICGC and TCGA cohorts. Data are represented as mean (SD), median (OQR), *n* (%) or *n*.
(PDF)

## Abbreviations

CTLA4: cytotoxic T lymphocyte–associated protein 4
GSEA: gene set enrichment analysis
MR: master regulator
PDAC: pancreatic ductal adenocarcinoma
PD-1: programmed cell death protein 1
PD-L1: programmed death-ligand 1
TF: transcription factor
VIPER: Virtual Inference of Protein-activity by Enriched Regulon analysis
