# Supplementary material for: Master Regulators of Oncogenic KRAS Response in Pancreatic Cancer: An Integrative Network Biology Analysis
Source: PLoS Med. 2017 Jan 31;14(1):e1002223. doi: 10.1371/journal.pmed.1002223 (PMC5283690; doi:10.1371/journal.pmed.1002223)
Supplement: S1 Computational Analysis — Also included is a PDF output of the complied code. (ZIP) [file pmed.1002223.s011.zip › S1_Sweave/S1_Sweave.pdf]

Supporting Information for –Kras-associated regulatory hubs in pancreatic cancer–

October 19, 2016

Contents

I Data Pre-processing **5**

1 Data source and download **5**
1.1 Data description 5
1.2 TCGA data access 5

2 Pre-processing Affymetrix array data **6**
2.1 Libraries for processing Affymetrix array data 6
2.2 Probe quality control 6
2.3 Normalisation 9
2.4 Probe annotation 11

3 Processing ICGC array data **12**
3.1 Preparing the gene expression matrix 12
3.2 Batch effect removal 13
3.3 Probe annotation 15

4 Processing TCGA data **16**
4.1 Gene counts 16
4.2 Transforming TCGA RNA-seq data 16

5 Saving the data **17**

II Master Regulator Analysis **18**

6 Network inference and analysis **18**
6.1 Transcription network inference pipeline 18
6.1.1 Check the consistency of the input data and remove non-informative probes 18
6.1.2 Partial correlation computation 18
6.1.3 Calculation of False discovery rates 19
6.2 Network fusion 19

7 Obtaining the KRAS signature **20**
7.1 Pathway analysis 23

8 Finding Master Regulators of KRAS **30**
8.1 KRAS null model 30

8.2 Preparing network data 30
8.3 msVIPER analysis 30

9 Network comparisons **32**
9.1 Edge correlation between pairs of networks 32
9.2 Network activity comparison 34

10 Community search algorithm to identify core pathways **36**
10.1 Pathway analysis of the identified TF signatures 37

III Characterizing Pancreatic Cancer subtypes **41**

11 Subtype analysis **41**
11.1 PDAC subtypes for the TCGA and ICGC cohorts 41
11.2 Evaluation of the best number of clusters 41
11.3 Comparing the different subtypes with previous datasets 46

12 Survival analysis **48**
12.1 Survival differences in the ICGC cohort 50
12.2 Survival differences in the TCGA cohort 53

13 Analysis of mutation data **59**

14 References **64**

15 Session Info **66**

Description

This document contains a set of documentation, R code, results and datasets to fully reproduce the analysis and results of the correspondent publication. The full documentation is split into three parts:

- Part I: Data Pre-processig: Pre-processing of gene expression data.
- Network inference, network fusion and identification of master regulators of KRAS response.
- tumor subtype, survival, and mutational anlaysis

We first declare a function that will check if the data required for the analysis is within the working directory of the Sweave file:

```
#check directory structure
dataPath <- file.path(getwd(),"data/")
codePath <- file.path(getwd(),"code/")
depPath <-  file.path(getwd(),"dependencies/")

#Download the data if if does not exist already
if (!file.exists(dataPath)) { dir.create(dataPath) }
if (!file.exists(codePath)) { dir.create(codePath) }
if (!file.exists(depPath)) { dir.create(depPath) }

site <- "www.lightcone.net/KRAS-MasterRegulators-S1/"
fnames <- c("dependencies/hgu133plus2hsentrezg.db_19.0.0.tar.gz",
            "dependencies/hgu133plus2hsentrezgcdf_19.0.0.tar.gz",
            "dependencies/hgu133plus2hsentrezgprobe_19.0.0.tar.gz",
            "code/KRAS-master-regulators-S1_functions.R",
            "data/figure2B.png",
            "data/GexpMatricesConverted.RData",
            "data/KRAS-master-regulators-S1_data.RData",
            "data/MyTable_GEOdata.txt",
            "data/PreComputedCombNet.RData",
            "data/PreComputedKRASnullmodel.RData",
            "data/PreComputedKRASsig.RData",
            "data/PreComputedKRASsigPathway.RData",
            "data/PreComputedMRspathwayAnlaysis.RData",
            "data/PreComputedNetworks.RData",
            "data/TCGASeqcounts.RData",
            "data/PreComputedTCGAfreqmutadata.RData")

for (f in fnames) {
    if (!file.exists(file.path(getwd(),f))) {
      download.file(file.path(site,f),file.path(getwd(),f),"curl")
    }
}
```

```
#Load the package manager function
source("code/KRAS-master-regulators-S1_functions.R")

#download the data used for the analysis in this document
load("data/KRAS-master-regulators-S1_data.RData")
```

Load the libraries and data that we utilise for the analyses.

```
pkgTest("AnnotationDbi")
pkgTest("org.Hs.eg.db")
```

```
pkgTest("hgu133plus2hsentrezg.db", tar="dependencies/hgu133plus2hsentrezg.db_19.0.0.tar.gz", repos=NULL)
pkgTest("hgu133plus2hsentrezgcdf", tar="dependencies/hgu133plus2hsentrezgcdf_19.0.0.tar.gz", repos=NULL)
pkgTest("hgu133plus2hsentrezgprobe", tar="dependencies/hgu133plus2hsentrezgprobe_19.0.0.tar.gz", repos=NUL[illegible]
Packages <- c("affy","affyPLM","knitr","sva","made4","devtools","xtable","Rsubread","igraph","reshape2","l[illegible]
out <- sapply(Packages, pkgTest)
```

Part I

Data Pre-processing

1 Data source and download

1.1 Data description

Raw gene expression data files were obtained from Gene Expression Omnibus (GEO) (Edgar et al., 2002) for a total of five independent pancreatic-related studies. All five downloaded data sets were generated on Affymetrix GeneChip Human Genome U133 Plus 2.0 array. Additionally, we obtained microarray gene expression data from the ICGC project for 242 primary PDAC tumors (Bailey et al., 2016). Microarray data for the ICGC dataset was generated on Illumina HumanHT-12 v4.0 beadchip. The Lumi normalisation algorithm was applied using bgAdjust.affy quantile log2 as per the manifest. Finally, we used a total of 178 Pancreatic adenocarcinoma Primary Solid Tumor RNA-seq gene expression data from the TCGA consortium. TCGA data was downloaded from CGHub.

	GEO	Paper	Cases
1	GSE17891	Collisson et al., 2011	27 PDAC + 19 cell lines
2	GSE15471	Badea et al., 2008	36 PDAC + 36 Normal-adjacent
3	GSE16515	Pei et al., 2009	36 PDAC + 16 Normal-adjacent
4	GSE32676	Donahue et al., 2012	25 PDAC + 7 Non-malignant Pancreas
5	GSE2109	Expo project*	17 PDAC
6	–	ICGC project	242 PDAC
7	–	TCGA project	178 PDAC

Table 1: Overview of the expression profiling datasets selected for the QC and Network analysis. For the Network analysis only the PDAC tumor datasets were used. *https://expo.intgen.org/geo/home.do

1.2 TCGA data access

To download the TCGA data, we used gtdownload command line tool version 3.8.7-45 downloaded from https://cghub.ucsc.edu/software/downloads.html (GeneTorrent-3.8.7-45.pkg). The metadata file ("manifest.xml") for 178 RNA-seq cases was accessed from the CGHub Data Browser in August 2015 https://browser.cghub.ucsc.edu. The following search filters were used:

Found 178 results. Applied filter(s):

- Assembly: GRCh37/HG19 (GRCh37 OR GRCh37-lite OR GRCh37_BI_Variant OR GRCh37-lite-+-HPV_Redux-build OR GRCh37-lite_WUGSC_variant_1 OR GRCh37-lite_WUGSC_variant_2 OR HG19 OR HG19_Broad_variant OR HS37D5), GRCh37, GRCh37-lite, GRCh37_BI_Variant, GRCh37-lite-+-HPV_Redux-build, GRCh37-lite_WUGSC_variant_1, GRCh37-lite_WUGSC_variant_2, HG19, HG19_Broad_variant, HS37D5
- Disease: Pancreatic adenocarcinoma (PAAD)
- Study: TCGA (phs000178), CCLE (*Other_Sequencing_Multiisolate)
- Sample Type: Primary Solid Tumor (01 OR TARGET_01)
- State: Live (live)
- Library Type: RNA-Seq

2 Pre-processing Affymetrix array data

2.1 Libraries for processing Affymetrix array data

The affy objects used in this session depend on the CDF package *hgu133plus2hsentrezgcdf*. Both, the probe sequence mapping file and the custom CDF package can be downloaded from the website http://brainarray.mbni.med.umich.edu/brainarray/Database/CustomCDF/genomic_curated_CDF.asp

In detail, we used the custom CDF package for the HGU133Plus2 platform (version 19.0.0) that can be downloaded here:

- http://brainarray.mbni.med.umich.edu/Brainarray/Database/CustomCDF/19.0.0/entrezg.asp

The source file for the corresponding R package can be downloaded here:

- http://mbni.org/customcdf/19.0.0/entrezg.download/hgu133plus2hsentrezgcdf_19.0.0.tar.gz

And the qcdef files were obtained from:

- http://web.mit.edu/r/r_v3.0.1/lib/R/library/simpleaffy/extdata/hgu133plus2cdf.qcdef

2.2 Probe quality control

Quality control (QC) analysis was performed for every independent study to identify problematic arrays. QC analysis was performed using *affy* and *affyPLM* Bioconductor R packages (Gautier et al., 2004). Firstly, we create an array object per gene

```
#Studies analysed:
table(GEOannot[,"tissue"], GEOannot[,"study"])

##
##             Badea08 Collisson11 Donahue12 exp005 Pei09
##   cell_line       0          20         0      0     0
##   Normal         36           0         7      0    16
##   PDAC           36          27        25     17    36

#affy object per study
GEObatchlist

## $Badea08
## AffyBatch object
## size of arrays=1164x1164 features (53 kb)
## cdf=HGU133Plus2_Hs_ENTREZG (19764 affyids)
## number of samples=72
## number of genes=19764
## annotation=hgu133plus2hsentrezg
## notes=
##
## $Collisson11
## AffyBatch object
## size of arrays=1164x1164 features (40 kb)
## cdf=HGU133Plus2_Hs_ENTREZG (19764 affyids)
## number of samples=47
## number of genes=19764
## annotation=hgu133plus2hsentrezg
## notes=
##
## $Donahue12
```

```
## AffyBatch object
## size of arrays=1164x1164 features (33 kb)
## cdf=HGU133Plus2_Hs_ENTREZG (19764 affyids)
## number of samples=32
## number of genes=19764
## annotation=hgu133plus2hsentrezg
## notes=
##
## $exp005
## AffyBatch object
## size of arrays=1164x1164 features (25 kb)
## cdf=HGU133Plus2_Hs_ENTREZG (19764 affyids)
## number of samples=17
## number of genes=19764
## annotation=hgu133plus2hsentrezg
## notes=
##
## $Pei09
## AffyBatch object
## size of arrays=1164x1164 features (43 kb)
## cdf=HGU133Plus2_Hs_ENTREZG (19764 affyids)
## number of samples=52
## number of genes=19764
## annotation=hgu133plus2hsentrezg
## notes=
```

The *affyPLM* package fits probe-level robust regressions (probe-level model, PLM) to obtain probe-set summaries. The Relative Log Expression (RLE) and the Normalized Unscaled Standard Error (NUSE) were derived from the PLM and used to quantitatively access array quality.

```
#probe-level metric calculations per study
library(affyPLM)
PLMlist <- lapply(GEObatchlist,fitPLM)
```

The Relative Log Expression (RLE) values are computed for each probe-set in the array by calculating the ratio between the expression of a probe-set and the median expression of each probe-set across all arrays of the experiment. Since most probes are not expected to change across the arrays, the distribution of these log-ratios should be similar in range and be centered around 0.

NUSE values represents the individual probe standard error (SE) from the PLM fit. The SE values are normalised at the probe-level across the arrays so that the distribution of SE values across all arrays should be centered around 1.

A boxplot of RLE and NUSE values is represented for each array in each study in the figures below:

```
par(mfrow=c(5,2), mar=c(2,2,2,2))
outliers=list()
outliers[["Badea08"]] <- plotQC_perstudy("Badea08",PLMlist[["Badea08"]],GEOannot)
legend("topleft",c("Normal","Tumor"),col=c("red","darkblue"),pch=15)
outliers[["Collisson11"]] <- plotQC_perstudy("Collisson11",PLMlist[["Collisson11"]],GEOannot)
legend("topleft",c("Normal","Cell line"),col=c("red","purple"),pch=15)
outliers[["Donahue12"]] <- plotQC_perstudy("Donahue12",PLMlist[["Donahue12"]],GEOannot)
legend("topleft",c("Normal","Tumor"),col=c("red","darkblue"),pch=15)
outliers[["exp005"]] <- plotQC_perstudy("exp005",PLMlist[["exp005"]],GEOannot)
outliers[["Pei09"]] <- plotQC_perstudy("Pei09",PLMlist[["Pei09"]],GEOannot)
legend("topleft",c("Normal","Tumor"),col=c("red","darkblue"),pch=15)
```

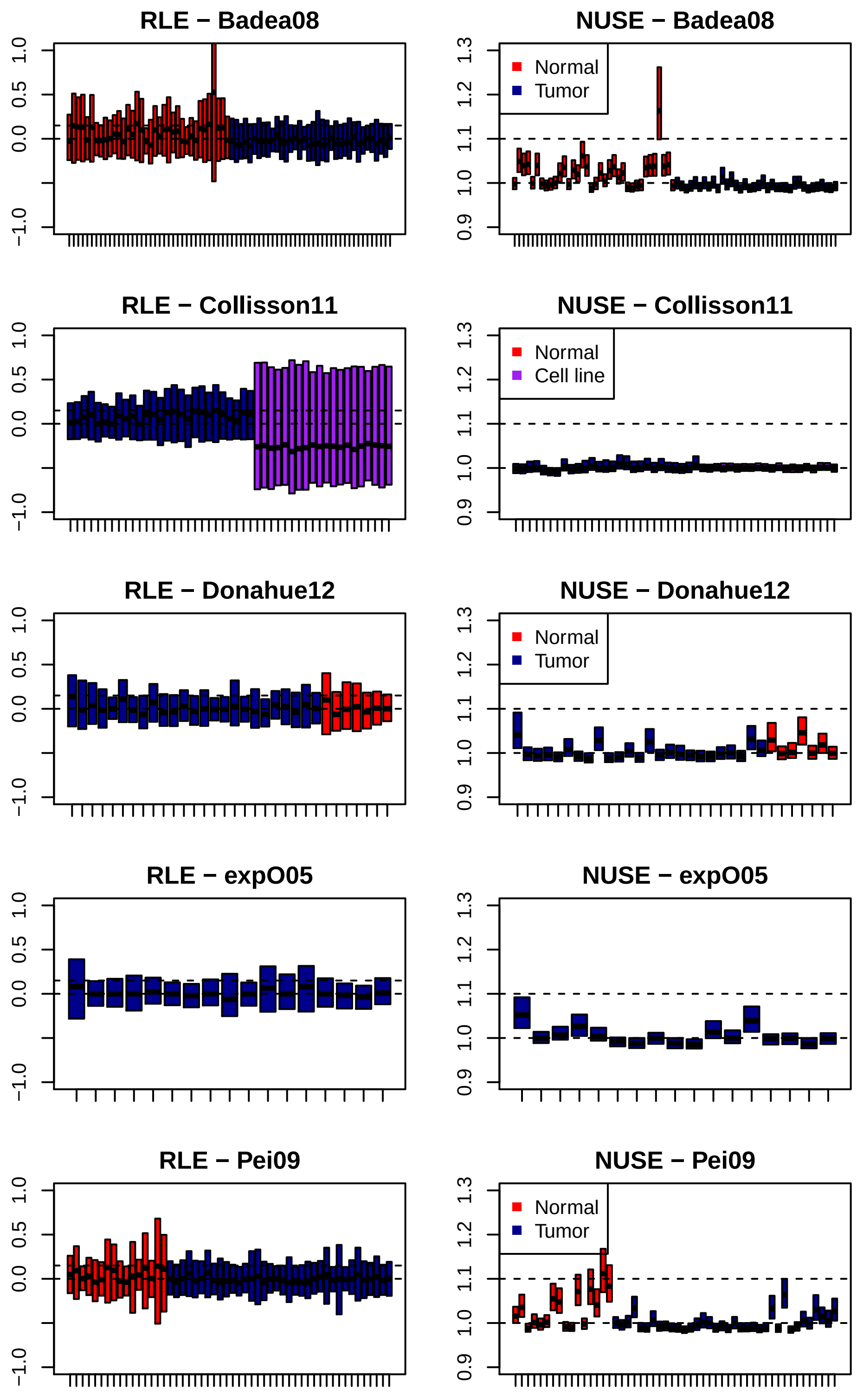

Figure 1: RLE and NUSE distributions for the seven cohorts of PDAC gene expression profiling used.

The boxplots allowed checking the following:

1. If all distributions have RLE and NUSE values centered near 0 and 1, respectively
2. If there is any array with an higher spread of NUSE or RLE values than that of other arrays from the same experiment.

We used the RLE threshold of 0.15 and a NUSE threshold of 1.1 to identify outliers. A total of 5 arrays were identified as outliers:

```
RLE_bad <- unlist(sapply(outliers, function(x) {return(x$RLE_bad)}))
NUSE_bad <- unlist(sapply(outliers, function(x) {return(x$NUSE_bad)}))
arrays_to_delete <- unique(c(RLE_bad,NUSE_bad))

library(knitr)
kable(GEOannot[RLE_bad,])
```

	study	tissue
GSM388094.CEL.gz	Badea08	Normal
GSM388110.CEL.gz	Badea08	Normal
GSM388111.CEL.gz	Badea08	Normal
GSM446752.CEL.gz	Collisson11	PDAC

```
kable(GEOannot[NUSE_bad,])
```

	study	tissue
GSM388111.CEL.gz	Badea08	Normal
GSM414972.CEL.gz	Pei09	Normal

Since the downstream analysis will only focus on PDAC data, only 1 array (GSM446752.CEL.gz from Collisson et al., 2011) was actually excluded from downstream analysis

```
#remove bad arrays and take only the PDAC tumors
batchlist.clean <- lapply(names(GEObatchlist), function(study) {
  annot <- GEOannot_per_study[[study]]
  dataAffy <- GEObatchlist[[study]]
  names <- rownames(annot)[annot[,"tissue"] == "PDAC"]
  dataPDAC <- dataAffy[,names]
  if (any(sampleNames(dataPDAC) %in% arrays_to_delete)) {
    badArray <- as.numeric(na.omit(match(arrays_to_delete, sampleNames(dataPDAC))))
    print (paste0("removed array from ", study, ": ", colnames(dataPDAC)[badArray]))
    return(dataPDAC[, -badArray])
  }else{
    return(dataPDAC)
  }
})

## [1] "removed array from Collisson11: GSM446752.CEL.gz"

names(batchlist.clean) <- names(GEObatchlist)
```

2.3 Normalisation

For each of the five cohorts, the resulting matrix was processed using the custom CDF version 19 (http://brainarray.mbni.med.umich.edu/ and RMA (Robust Multichip Averaging) (Irizarry et al. 2003) normalisation in R (www.r-project.org).

```
eset <-lapply(batchlist.clean, rma)
```

```
# Plot Not-normalized vs Normalized CEL files
par(mfrow=c(5,2), mar=c(2,2,2,2))
```

```
for (study in names(batchlist.clean)) {
  boxplot(batchlist.clean[[study]], main=paste(study, "before RMA"))
  boxplot(eset[[study]], main=paste(study,"after RMA"))
}
```

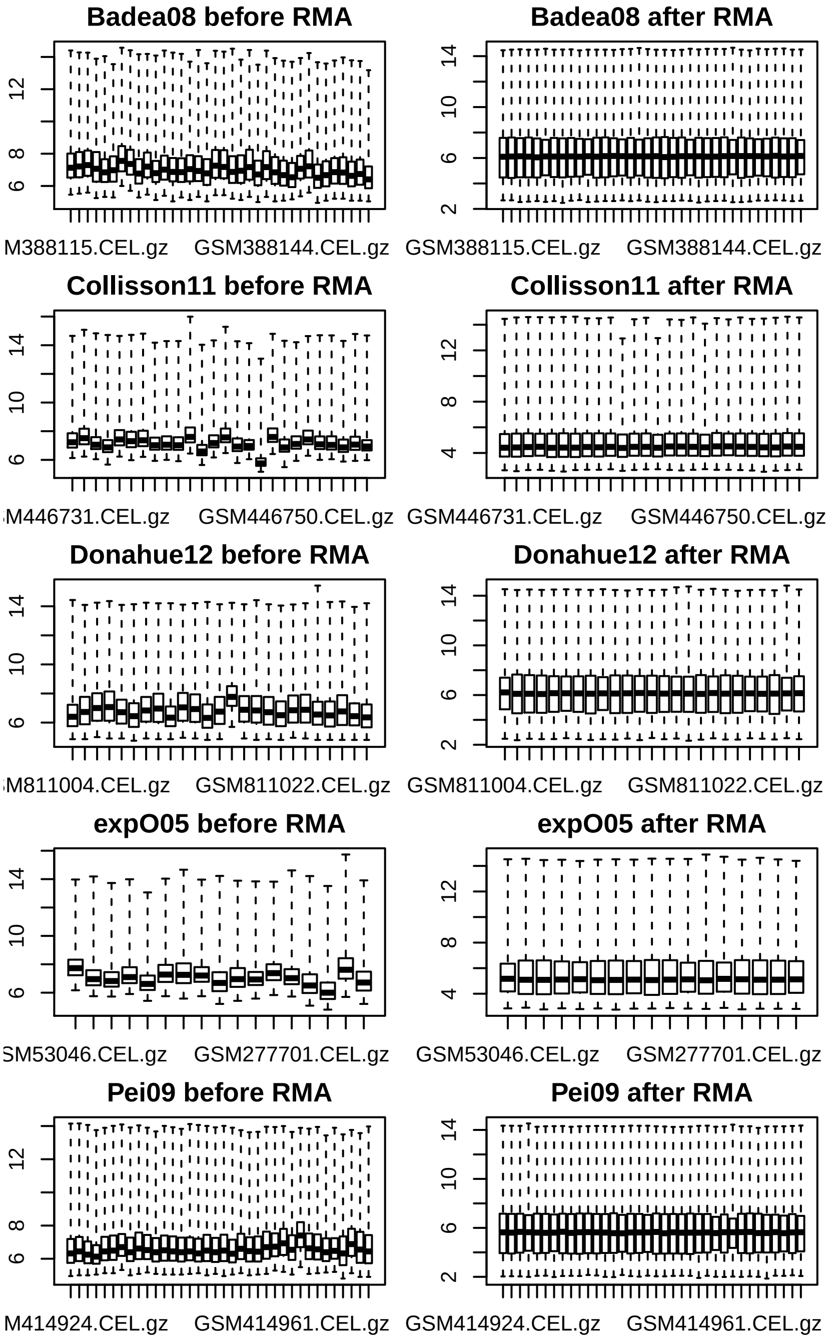

Figure 2: Distribution of probe intensity scores before and after RMA normalisation.

2.4 Probe annotation

We converted all probe IDs to Entrez ids using the standard "db" file for the Human U133 Plus 2.0 affymetrix array. We filtered the final expression matrix so that it contained only probes for which there was an correspondent Entrez gene ID.

```
ex <- lapply(eset, exprs)
geneIDs <- lapply(ex, getGeneExpIDs)

#any dups?
for (gene.IDs in geneIDs) {
  NrNAs = sum(is.na(gene.IDs[,2]))
  NrDups = sum(duplicated(gene.IDs[!(is.na(gene.IDs[,2])),2]))
  print (paste("there are", nrow(gene.IDs), "probe,gene IDs"))
  print (paste("there are", NrNAs, "geneIDs without any ENTREZ symbol"))
  print (paste("there are", NrDups, "duplicated geneIDs"))
}

## [1] "there are 19764 probe,gene IDs"
## [1] "there are 228 geneIDs without any ENTREZ symbol"
## [1] "there are 0 duplicated geneIDs"
## [1] "there are 19764 probe,gene IDs"
## [1] "there are 228 geneIDs without any ENTREZ symbol"
## [1] "there are 0 duplicated geneIDs"
## [1] "there are 19764 probe,gene IDs"
## [1] "there are 228 geneIDs without any ENTREZ symbol"
## [1] "there are 0 duplicated geneIDs"
## [1] "there are 19764 probe,gene IDs"
## [1] "there are 228 geneIDs without any ENTREZ symbol"
## [1] "there are 0 duplicated geneIDs"
## [1] "there are 19764 probe,gene IDs"
## [1] "there are 228 geneIDs without any ENTREZ symbol"
## [1] "there are 0 duplicated geneIDs"

ex <- lapply(eset, exprs)
lapply(ex, dim)

## $Badea08
## [1] 19764    36
##
## $Collisson11
## [1] 19764    26
##
## $Donahue12
## [1] 19764    25
##
## $exp005
## [1] 19764    17
##
## $Pei09
## [1] 19764    36

GEOArray.Gexp <- lapply(ex, function(x) {
  ids <- getGeneExpIDs(x)
  cv.filter(x, ids)
})

## [1] "removed 228 entries without annotation"
```

```
## [1] "removed 0 duplicated entries"
## [1] "removed 228 entries without annotation"
## [1] "removed 0 duplicated entries"
## [1] "removed 228 entries without annotation"
## [1] "removed 0 duplicated entries"
## [1] "removed 228 entries without annotation"
## [1] "removed 0 duplicated entries"
## [1] "removed 228 entries without annotation"
## [1] "removed 0 duplicated entries"
```

```
lapply(GEOArray.Gexp, function(x) {dim(x[[1]])})
```

```
## $Badea08
## [1] 19536    36
##
## $Collisson11
## [1] 19536    26
##
## $Donahue12
## [1] 19536    25
##
## $exp005
## [1] 19536    17
##
## $Pei09
## [1] 19536    36
```

3 Processing ICGC array data

3.1 Preparing the gene expression matrix

We combined all normalised gene expression values in a matrix:

```
head(ICGCbatchinfo)
```

```
##        qcmg_processing_id biospecimen_id qcmg_project submitted_patient_id   icgc_id
## 1 ICGC-ABMB-20100303-07-TR        8012195    APGI_1955            APGI_1955 ICGC_0007
## 2 ICGC-ABMB-20100303-09-TR        8012205    APGI_1956            APGI_1956 ICGC_0008
## 3 ICGC-ABMB-20100303-10-TR        8012209    APGI_2040            APGI_2040 ICGC_0025
## 4 ICGC-ABMJ-20100603-06-TR        8013037    APGI_2051            APGI_2051 ICGC_0032
## 6 ICGC-ABMJ-20100702-06-TR        8013915    APGI_1830            APGI_1830 ICGC_0004
## 7 ICGC-ABMJ-20100702-09-TR        8013931    APGI_1966            APGI_1966 ICGC_0012
##   other_publication_id      sample_code material                      description
## 1            GARV_0034 7:Primary tumour    2:RNA                           Cancer
## 2            GARV_0035 7:Primary tumour    2:RNA                           Cancer
## 3            GARV_0062 7:Primary tumour    2:RNA                           Cancer
## 4            GARV_0071 7:Primary tumour    2:RNA ID:2051 Panc-T Parent ID: 8011635
## 6            GARV_0029 7:Primary tumour    2:RNA                ID:1830 Panc-T RNA
## 7            GARV_0041 7:Primary tumour    2:RNA                ID:1966 Panc-T RNA
##   date_received bioa_rin_score nanodrop_concentration nanodrop_260_280 nanodrop_260_230
## 1    03/03/2010           7.50                  66.25             2.02             0.55
## 2    03/03/2010           8.00                  68.23             2.08             1.73
## 3    03/03/2010           8.35                  53.72             2.03             1.82
```

```
## 4    03/06/2010          9.50                 168.59              2.07              1.29
## 6    02/07/2010          7.98                 242.95              2.09              1.74
## 7    02/07/2010          8.80                 204.09              2.06              1.54
##   sample_qpure_score arrays_completed Scannedr BATCH
## 1                 NA     5445316009_D        1     1
## 2                 NA     5445316009_E        1     1
## 3                 NA     5445316009_F        1     1
## 4                 NA     5445316009_G        1     1
## 6                 NA     5445316009_I        1     1
## 7                 NA     5445316009_J        1     1
```

```
ICGCGexp[1:10,1:10]
```

```
##                 8012195   8012205   8012209   8012328   8012394   8012428   8012677
## ILMN_1343291 13.942443 13.819479 14.040784 13.942443 13.930886 14.092015 14.040784
## ILMN_1343295 11.852576 11.345243 13.352230 11.737992 12.396142 12.091738 11.350949
## ILMN_1651199  2.123852  2.012364  2.005809  1.871987  2.140078  2.752428  1.987634
## ILMN_1651209  2.304978  2.516348  2.451407  2.137959  2.421264  3.660697  1.902012
## ILMN_1651210  2.546217  2.410674  2.208591  2.172246  2.113216  2.116485  2.201574
## ILMN_1651221  3.029262  2.822703  2.159942  2.492385  2.298454  2.159337  3.695020
## ILMN_1651228 12.162862 12.015299 11.559326 12.275019 11.238307 11.857365 12.586567
## ILMN_1651229  6.901288  7.071891  7.456835  8.029240  6.874039  6.717896  5.807718
## ILMN_1651230  2.002684  2.579393  2.331634  2.586197  2.238173  1.974890  2.267989
## ILMN_1651232  3.161219  3.453613  3.458834  2.751046  2.858534  3.363544  4.458366
##                 8012776   8012790   8012856
## ILMN_1343291 13.987400 14.010251 13.885775
## ILMN_1343295 13.137923 12.262701 11.454908
## ILMN_1651199  2.829044  2.273209  2.459653
## ILMN_1651209  1.933946  2.141534  2.040283
## ILMN_1651210  2.372229  2.541454  2.347386
## ILMN_1651221  3.061494  3.894246  2.523510
## ILMN_1651228 11.284704 12.128771 12.791030
## ILMN_1651229  6.496272  6.292950  6.636473
## ILMN_1651230  2.620492  2.102784  2.367027
## ILMN_1651232  3.211214  2.681778  2.430957
```

```
dim(ICGCGexp)
```

```
## [1] 47265   242
```

3.2 Batch effect removal

To remove mild inter-study effects ComBat algorithm implemented in the *sva* R package was used. `ComBat` is a popular approach to remove batch effects developed by Johnson et al., 2007.

First, we combined the information between batch numbers and array IDs:

```
library(sva)
library(made4)
batches <- factor(ICGCbatchinfo$BATCH[match(colnames(ICGCGexp), ICGCbatchinfo$biospecimen_id)])
```

```
ex.combat <- ComBat(dat=ICGCGexp,batch=batches, mod=NULL)
```

```
## Found 19 batches
## Adjusting for 0 covariate(s) or covariate level(s)
## Standardizing Data across genes
```

```
## Fitting L/S model and finding priors
## Finding parametric adjustments
## Adjusting the Data
```

We performed a PCA (Principal Components Analysis) to understand if there were any remaining batch effects in the data.

```
gTypeCols <- rainbow(length(unique(batches)))

par(mfrow=c(1,2))
fit <- princomp(ICGCGexp, cor=TRUE)
plot(fit$loadings[,1], fit$loadings[,2],
     col=gTypeCols[batches],pch=19,
     xlab="PC1 loadings",
     ylab="PC2 loadings",
     main="Before ComBat")
legend("topright", legend = levels(batches),
       col = gTypeCols, lty = 1, lwd = 5,
       cex = 0.5)

fit2 <- princomp(ex.combat, cor=TRUE)
plot(fit2$loadings[,1], fit2$loadings[,2],
     col=gTypeCols[batches],pch=19,
     xlab="PC1 loadings",
     ylab="PC2 loadings",
     main="After ComBat")
legend("topright", legend = levels(batches),
       col = gTypeCols, lty = 1, lwd = 5,
       cex = 0.5)
```

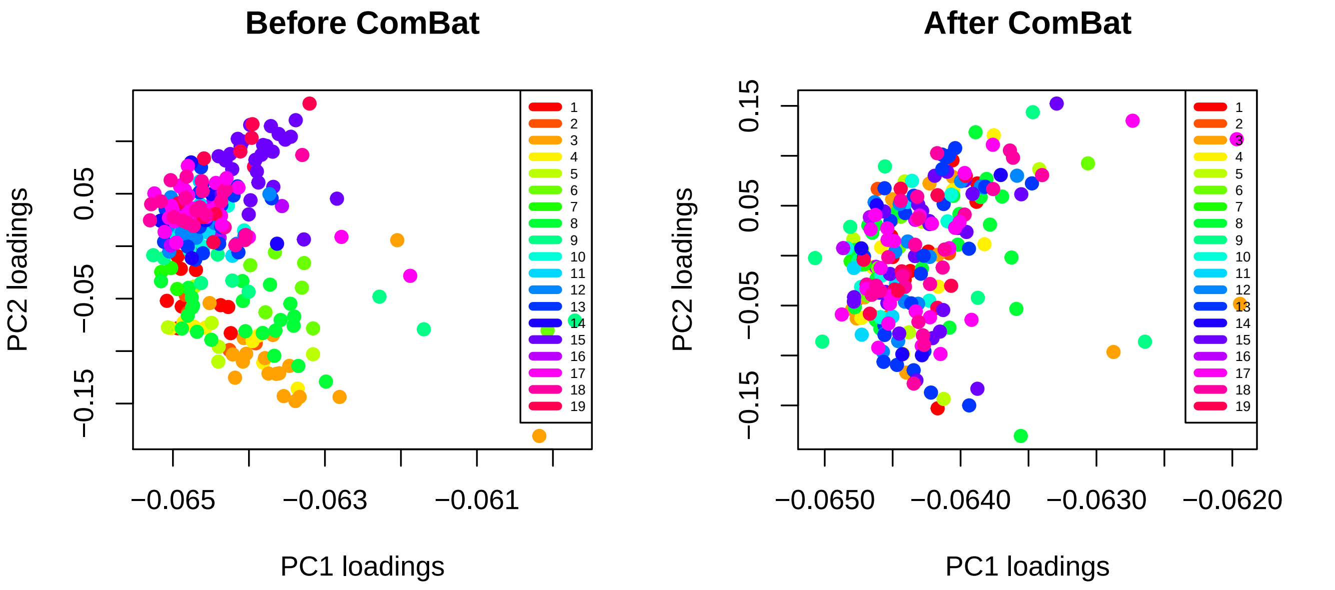

Figure 3: Principal components analysis (PCA) of gene expression data from the ICGC cohort before and after batch adjustment with ComBat algorithm

3.3 Probe annotation

We downloaded a table of Illumina IDs (PROBEID, ENTREZ, SYMBOL) from http://www.ncbi.nlm.nih.gov/geo/query/acc.cgi?acc=GPL10558

```
head(ICGCprobeinfo)
```

```
##                   PROBEID ENTREZ     SYMBOL
## ILMN_3166687 ILMN_3166687     NA ERCC-00162
## ILMN_3165565 ILMN_3165565     NA ERCC-00071
## ILMN_3164808 ILMN_3164808     NA ERCC-00009
## ILMN_3165363 ILMN_3165363     NA ERCC-00053
## ILMN_3166504 ILMN_3166504     NA ERCC-00144
## ILMN_3164750 ILMN_3164750     NA ERCC-00003
```

all 47,265 probe IDs in the gene expression table are in the `ICGCprobeinfo` file

```
all(ICGCprobeinfo$ID %in% rownames(ICGCGexp))

## [1] TRUE

all(rownames(ICGCGexp) %in% ICGCprobeinfo$ID)

## [1] FALSE

length(rownames(ICGCGexp))

## [1] 47265

dim(ICGCprobeinfo)

## [1] 47265     3
```

We checked that there were no duplicated probeIDs in the table

```
any(duplicated(ICGCprobeinfo$PROBEID))

## [1] FALSE
```

However, there are some probes that correspond to duplicated ENTREZ IDs

```
any(duplicated(ICGCprobeinfo$ENTREZ[!(is.na(ICGCprobeinfo$ENTREZ))]))

## [1] TRUE
```

We filtered the final expression matrix so that it contained only probes for which there was an correspondent Entrez gene ID, and duplicated IDs were filtered out so that the ID with the highest coefficient of variation is kept

```
ICGCArray.Gexp <- cv.filter(ICGCGexp, ICGCprobeinfo)

## [1] "removed 3359 entries without annotation"
## [1] "removed 13444 duplicated entries"

dim(ICGCGexp)

## [1] 47265   242

dim(ICGCArray.Gexp[[1]])

## [1] 30462   242
```

4 Processing TCGA data

4.1 Gene counts

We used *Rsubread* package to count the reads in genes. *Rsubread* has a number of built-in annotations that we can make use of.

`featureCounts` will use the in-built SAF-format annotation file. By default, the annotations (each row is a feature) are grouped into meta-features using their values in the `GeneID` column. We used the default arguments for read summarisation:

- allowMultiOverlap=FALSE: reads are only allowed to be assigned to only 1 feature (or meta-feature)
- isPairedEnd=FALSE. Individual reads are counted instead of read-pairs
- strandSpecific=0. Does not preform strand-specific read counting
- minMQS=0. integer giving the minimum mapping quality score a read must satisfy in order to be counted.
- countMultiMappingReads=FALSE. This function uses "NH" tag to find multiple mappings. When set to FALSE multi-mapping reads/fragments are not counted
- ignoreDup=FALSE. Reads marked as duplicates are not featureCounts ignored
- nthreads=5. Number of threads used for running the function

To create the table of gene counts we used the following code:

```
library(Rsubread)
list_of_dirs <- gsub(".gto","", list.files(path = ".", pattern=".gto"))
filesToCount <- c()
for (dir in list_of_dirs){
  filesToCount <- c(filesToCount, file.path(dir,list.files(dir, pattern=".bam")[[1]]))
}

#counts
TCGASeqcounts <- featureCounts(filesToCount, annot.inbuilt="hg19", ignoreDup=F, nthreads=10)
```

4.2 Transforming TCGA RNA-seq data

We saved the R objects needed for further analysis (TCGASeqcounts and hg19_annot_SAF) in a RData file named "TCGASeqcounts.RData" (available in the "data" directory).

```
load("data/TCGASeqcounts.RData")
```

In RNA-Seq data, genes with larger average expression have on average larger observed variances across samples, that is, they vary in expression from sample to sample more than other genes with lower average expression. This phenomena is known as data heteroscedasticity. This effect is clearly visible when we plot the per-gene standard deviation (taken across the 178 samples), against the rank of the average expression. The plot below was taken by using the meanSdPlot function from the *vsn* package and demonstrates the mean-variance dependency clearly.

RNA sequencing data was transformed with variance-stabilizing transformation (VST) method. VST for RNA-seq data was purposed by Anders and Huber, 2010 and it is implemented in the *DESeq2* package.

```
library(DESeq2)

## Loading required package: GenomicRanges
## Loading required package: Rcpp
## Loading required package: RcppArmadillo

## Warning: replacing previous import 'S4Vectors::Position' by 'ggplot2::Position' when loading
'DESeq2'
```

```
library(vsn)
TCGASeqcounts$vsd <-varianceStabilizingTransformation(TCGASeqcounts$counts, blind=TRUE)
```

After VST the standard deviation becomes more constant along the whole dynamic range, but there are still unequal for all genes.

```
library(vsn)
par(mfrow=c(1,2))
meanSdPlot(as.matrix(TCGASeqcounts$counts),ylim=c(0,8e3),main="TCGA:counts")
meanSdPlot(as.matrix(TCGASeqcounts$vsd), ylim = c(0,2.5),main="TCGA:VST")
```

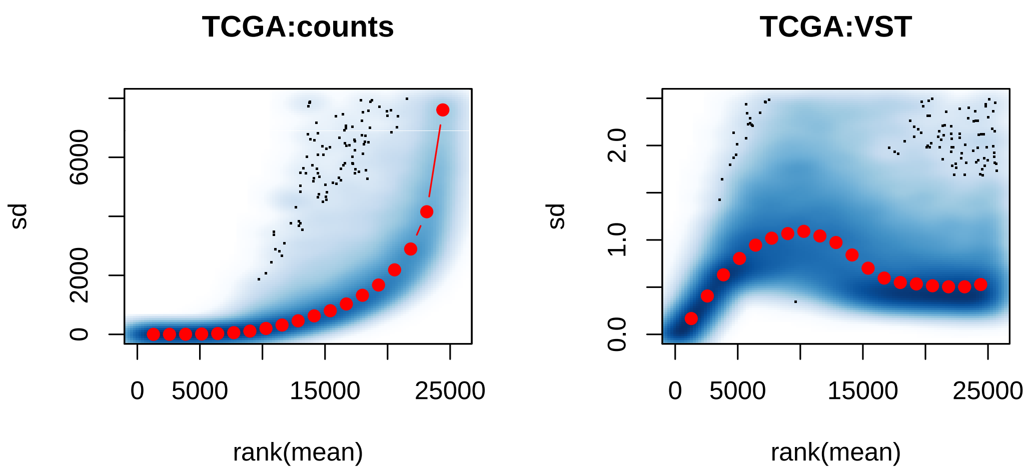

Figure 4: Per-gene standard deviation (taken across samples) against the rank of the mean for the PDAC TCGA datasets. Data variance depends on the mean value, i.e. genes having larger mean expression values also have larger variances

5 Saving the data

The final gene expression matrices were saved as RData objects:

```
TCGAseq.Gexp <- TCGASeqcounts$vsd
ICGCarray.Gexp <- ICGCArray.Gexp$gexp
Badea08.Gexp <- GEOArray.Gexp[["Badea08"]]$gexp
Collisson11.Gexp <- GEOArray.Gexp[["Collisson11"]]$gexp
Donahue12.Gexp <- GEOArray.Gexp[["Donahue12"]]$gexp
exp005.Gexp <- GEOArray.Gexp[["exp005"]]$gexp
Pei09.Gexp <- GEOArray.Gexp[["Pei09"]]$gexp
#save(TCGAseq.Gexp, ICGCarray.Gexp, Badea08.Gexp, Collisson11.Gexp, Donahue12.Gexp,
#     exp005.Gexp, Pei09.Gexp, file="data/GexpMatricesConverted.RData")
```

We provide the pre-computed R objects in the "data" directory:

```
load("data/GexpMatricesConverted.RData")
```

Part II

Master Regulator Analysis

6 Network inference and analysis

6.1 Transcription network inference pipeline

Network inference was done by co-expression analysis: if two genes show similar expression profiles across samples, they are supposed to share a regulatory relationship. We used partial correlations to infer rthe co-regulatory relationship between any two genes conditioned on all remaining genes

We implemented the R code in the R package *RTN* Version 1.11.1: *reconstruction of transcriptional networks and analysis of master regulators* (Fletcher et al., 2013; Castro et al., 2016), which is designed for the reconstruction of transcriptional networks. The pipeline is run in three steps (i) Check the consistency of the input data and remove non-informative genes (ii) compute partial correlations (iii) compute the significance of partial correlation scores.

6.1.1 Check the consistency of the input data and remove non-informative probes

The first step is to create an object of class TNI to be used with methods developed in RTN. To run RTN you will need gene expression dataset and a list of transcription factors or regulators. The list of transcription factors used in this study was derived from that used in a previous ARACNe/MRA publication (Carro et al., 2010) by converting Affymetrix probe IDs into the matching Entrez IDs.

We have done this for all seven PDAC cohorts used in this study:

```
Badea08.rtni <- new("TNI", gexp=Badea08.gexp, transcriptionFactors=tfs)
Collisson11.rtni <- new("TNI", gexp=Collisson11.gexp, transcriptionFactors=tfs)
Donahue12.rtni <- new("TNI", gexp=Donahue12.gexp, transcriptionFactors=tfs)
exp005.rtni <- new("TNI", gexp=exp005.gexp, transcriptionFactors=tfs)
Pei09.rtni <- new("TNI", gexp=Pei09.gexp, transcriptionFactors=tfs)
ICGCArray.rtni <- new("TNI", gexp=ICGCArray.gexp, transcriptionFactors=tfs)
TCGASeq.rtni <- new("TNI", gexp=TCGASeq.gexp, transcriptionFactors=tfs)
```

Secondly, just as described in the *RTN* package documentation, the generic function `tni.preprocess` is used to run several checks on the input data. At this step, RTN will check if gene symbols are consistently matched between the input datasets, and will filter out genes that exhibit low coefficient of variation (CV), below the CV median value.

```
Badea08.rtni <- tni.preprocess(Badea08.rtni)
Collisson11.rtni <- tni.preprocess(Collisson11.rtni)
Donahue12.rtni <- tni.preprocess(Donahue12.rtni)
exp005.rtni <- tni.preprocess(exp005.rtni)
Pei09.rtni <- tni.preprocess(Pei09.rtni)
ICGCArray.rtni <- tni.preprocess(ICGCArray.rtni)
TCGASeq.rtni <- tni.preprocess(TCGASeq.rtni)
```

6.1.2 Partial correlation computation

The function `tni2tpc.corpcor` was used to infer the transcriptional networks using partial correlations. This function works as a wrapper around the `pcor.shrink` function from the *corpcor* package, originally developed by Korbinian Strimmer's lab (Schäfer and Strimmer 2005; Opgen-Rhein and Strimmer 2007). Within the RTN wrapper `tni2tpc.corpcor`, the shrinkage function used to estimate the partial correlation matrix is `pcor.shrink` with default parameters. Firstly

the shrinkage estimates of partial correlation are computed for each gene-gene pair. Then, a subset of the symmetric matrix is removed to only consider TF-gene partial correlation estimates.

```
Badea08.rtni <- tni2tpc.corpcor(Badea08.rtni)
Collisson11.rtni <- tni2tpc.corpcor(Collisson11.rtni)
Donahue12.rtni <- tni2tpc.corpcor(Donahue12.rtni)
exp005.rtni <- tni2tpc.corpcor(exp005.rtni)
Pei09.rtni <- tni2tpc.corpcor(Pei09.rtni)
ICGCArray.rtni <- tni2tpc.corpcor(ICGCArray.rtni)
TCGASeq.rtni <- tni2tpc.corpcor(TCGASeq.rtni)
```

Due to the large-scale datasets and the computing power required to compute the transcription networks, we provide 7 pre-computed networks R object:

```
load("data/PreComputedNetworks.RData")
```

6.1.3 Calculation of False discovery rates

The significance of each partial correlation score is the last step within the `tni2tpc.corpcor` function. This step makes use of the `fdrtool` of the *fdrtool* package. The *fdrtool* package allows to estimate null values and the parameters of the null distribution for a variety of null models (p-values, z-scores, correlation coefficients, t-scores) (Strimmer 2008). The obtained RTN `TPC-Class` object contains the unfiltered edges with the corresponding p-values, q-values and probablity estimates as calculated with the `fdrtool` function. It is possible to apply a desired threshold to count how many significant edges exist in each network:

```
#how many are significant based on FDR cutoff Q=0.05?
a <- sapply(allNetworks, tpc.countedges, cutoff.method="qval", cutoff.tr=0.05, verbose=TRUE)

## Significant edges: 9033 Corresponding to 0.06% of all possible edges
## Significant edges: 959 Corresponding to 0.01% of all possible edges
## Significant edges: 3036 Corresponding to 0.02% of all possible edges
## Significant edges: 1988 Corresponding to 0.01% of all possible edges
## Significant edges: 12872 Corresponding to 0.08% of all possible edges
## Significant edges: 67821 Corresponding to 0.27% of all possible edges
## Significant edges: 338142 Corresponding to 1.62% of all possible edges

#how many are significant based on prob > 0.9 (i.e. local fdr < 0.1)?
a <- sapply(allNetworks, tpc.countedges, cutoff.method="prob", cutoff.tr=0.9, verbose=TRUE)

## Significant edges: 9442 Corresponding to 0.06% of all possible edges
## Significant edges: 928 Corresponding to 0.01% of all possible edges
## Significant edges: 3011 Corresponding to 0.02% of all possible edges
## Significant edges: 2128 Corresponding to 0.01% of all possible edges
## Significant edges: 12678 Corresponding to 0.08% of all possible edges
## Significant edges: 62460 Corresponding to 0.25% of all possible edges
## Significant edges: 271427 Corresponding to 1.3% of all possible edges
```

6.2 Network fusion

We combined the 7 derived networks by using the Stouffer's Z-score method (Stouffer et al., 1949). This method is based on the average of the Z-score values and allows for incorporation of sample sizes as the study weights. If the i_th Zscore is weighted by w_i, then the meta-analysis Zscore is:

$$Z \sim \frac{\sum_{i=1}^{k} w_i Z_i}{\sqrt{\sum_{i=1}^{k} w_i^2}}, \quad (1)$$

which follows a standard normal distribution under the null hypothesis.

Because in our study partial correlation scores can be either positive or negative, we use the two-sided p-value in the calculation of the Z_i. The two-sided p-value is equal to p_i/2, and the associated Zscore is:

$$Z_i = \phi - 1(1 - p_i/2) \quad (2)$$

To compute the fused Network we used the R function `tpc.fuseNetwork` from the R package *RTN*. This function computes the combined Z-score for the overall meta-analysis, and makes use of the fdrtool package to further calculate the associated q-values and FDR for each edge in the combined network.

```
combinedNetwork <- tpc.fuseNetwork(allNetworks)
```

Due to large computation time, we provide the combined network R object:

```
load("data/PreComputedCombNet.RData")
```

7 Obtaining the KRAS signature

An experimental design was constructed in a way that we have two treatments ("Mock" vs "Cre"). RNA samples were collected from six biological replicates. Two RNA samples were collected from each biological replicate (these are the technical replicates). Samples were hybridized to Illumina Mousev2 BeadChips using 24 arrays (1 sample per Bead-Chip). Differential expression analysis between the two groups ("Mock" vs "Cre") was carried out using *limma* Bioconductor package (Ritchie et al., 2015)

```
#design matrix
library(limma)
groups <- as.factor(KRAStargets$Sample_Group)
design = model.matrix(~0 + groups)
colnames(design) = sub("groups", "", colnames(design))

#aWeights Norm
aWeights <- arrayWeights(KRASgexp,design=design)
dotchart(aWeights,labels=KRAStargets$Sample_Name,
         groups=as.factor(KRAStargets$Sample_Group),pch=19,
         color= col_matrix[,"Sample_Group"],main="Array Weights")
```

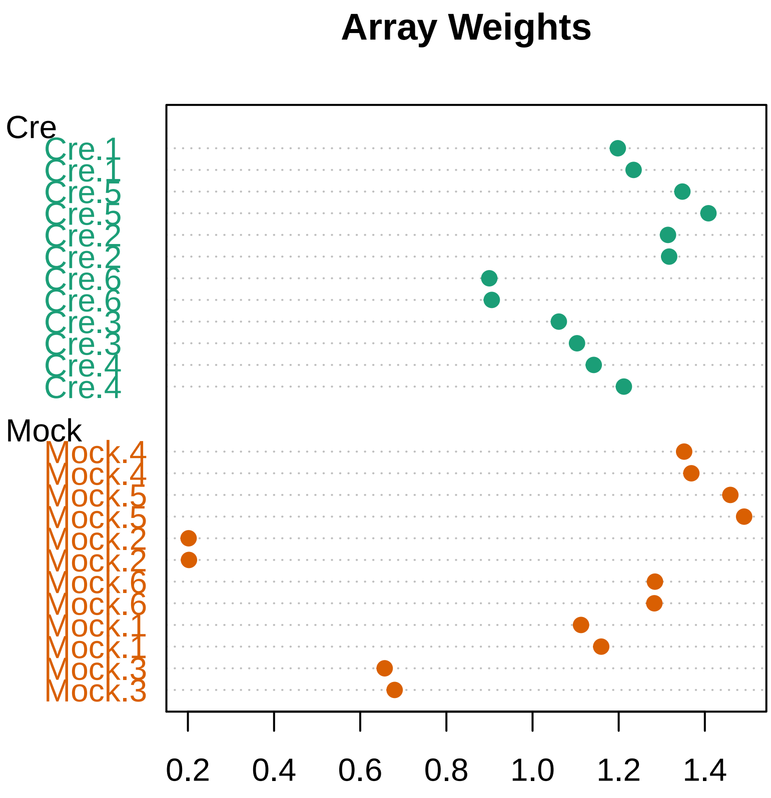

Figure 5: P95 Values per Group

The experiment had two levels of variability. First, there is the variation between biological replicates. Secondly, there the variability within replicates, i.e. the repeat measurements made on the same RNA sample (technical replicates). Therefore, we used the limma function `duplicateCorrelation` to estimate the correlation between measurements made on the same technical replicate. The inter-technical replicate correlation was then used as an input into the linear model fit:

```
fit <- getfit(KRASgexp, aWeights, KRAStargets, contrast="Cre-Mock",annotation=KRASannotation)
KRASsig <- getsighuman(fit[,c("IlluminaID","t","P.Value")],
                       annotTable=KRASannotTable, gexp=KRASgexp)
```

Due to large computation time, we provide the R object containing the KRAS signature and fit model:

```
load("data/PreComputedKRASsig.RData")
```

The initial dataset consisted of 46,235 unique probes, corresponding to 20,562 mouse genes (based on Entrez) and 19,575 mouse ID based on ENSEMBL IDs.

```
sig <- fit[,c("IlluminaID","t","P.Value")]
sigM <- merge(unique(sig), unique(KRASannotTable), by="IlluminaID", all.x=T)
```

```
dim(fit) #[1] 46235     19

## [1] 46235    19

length(unique(fit$ENTREZID)) #[1] 20562

## [1] 20562

length(unique(sigM$ENSEMBL)) #[1] 19575

## [1] 19575
```

The following volcano plot shows the log2 fold-changes and -log10 p-values obtained obtained for all 46,235 unique mouse probes:

```
fit$threshold <- as.factor(fit$P.Value <= 0.05 & abs(fit$logFC) >= 1) #for visualisation
up <- sum(fit$P.Value <= 0.05 & fit$logFC >= 1)
down <- sum(fit$P.Value <= 0.05 & fit$logFC <= -1)
g = ggplot(data=fit, aes(x=logFC, y=-log10(P.Value), colour=threshold)) +
  geom_point(alpha=0.4, size=1.75) +
  xlim(c(-3, 3)) + ylim(c(0, 15)) +
  xlab("log2 fold change") + ylab("-log10 p-value") +
  annotate("text", x=-2.5, y=11, label= paste("N=",down, "\n(down-regulated)"), col="#00BFC4" , size=3, fo
  annotate("text", x= 2.6, y=11, label= paste("N=",up, "\n(up-regulated)") , col="#00BFC4", size=3, fontfa
plot(g)
```

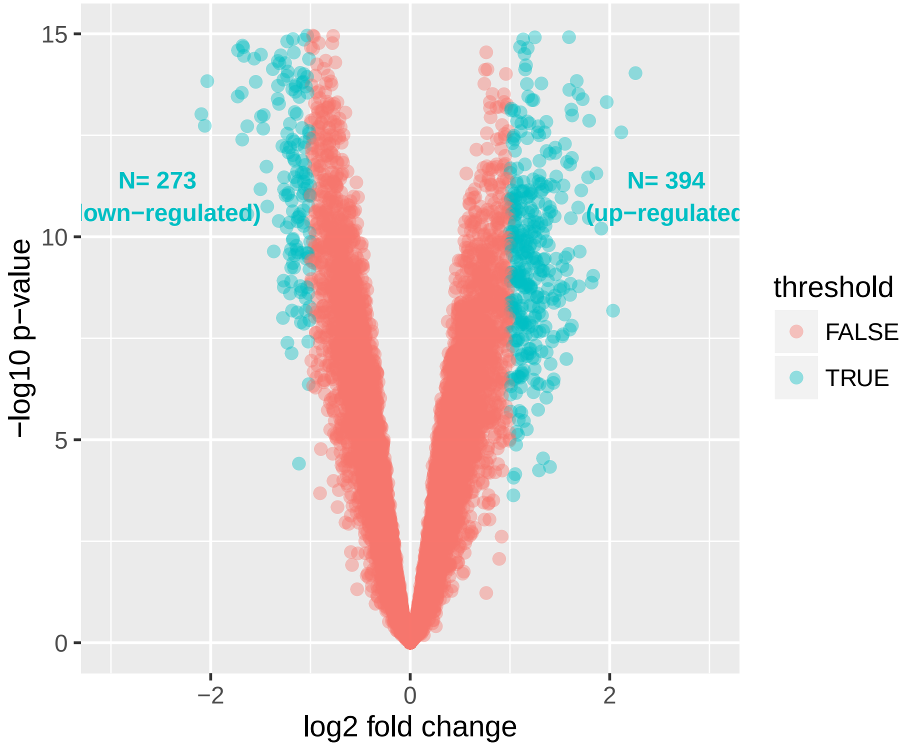

Figure 6: Volcano plot showing the magnitude of the differential gene expression between "Mock" and "Cre". Each dot represents one probe with detectable expression in both conditions. The colored dots mark the threshold (p <0.05 and log2 fold-change 1) for defining a gene as differentially expressed.

Based on BiomaRt homolog gene annotations, we obtained 17,032 unique human Entrez IDs.

```
nas <- which(is.na(sigM[,"hsapiens_entrezgene"]))
length(unique(sigM$hsapiens_entrezgene)) #[1] 17032

## [1] 17032
```

After removing bad quality probes and NA ids, we obtained 16,742 human Entrez IDs. Finally, we combined multiple mouse Illumina probe IDs mapping to the same human Entrez gene ID by taking the average t-statistic of all Illumina probes.

```
sig2 <- sigM[-nas,] #DEG is signature without the NAS
badquality <- which(sig2$PROBEQUALITY == "Bad")
sig2 <- sig2[-badquality,] #delete bad quality
length(unique(sig2$hsapiens_entrezgene)) #[1] 16742

## [1] 16742

length(KRASsig[[1]])

## [1] 16742
```

7.1 Pathway analysis

To identify enriched pathways and gene ontologies in the KRAS signature we performed a gene set enrichment analysis using the R package *HTSanalyzeR*. For the pathway analysis we used Kyoto Encyclopedia of Genes and Genomes (KEGG) and two GO gene set collections obtained from *KEGG.db* and *GO.db* R packages, respectively.

```
gsca <- pathwayAnalysis(fit, pvaluecutoff=0.05)
```

Due to long computation time, we provide the R object with the analysis results in the data folder:

```
load("data/PreComputedKRASsigPathway.RData")
```

```
#summarize results
HTSanalyzeR::summarize(gsca)

##
## -No of genes in Gene set collections:
##         input above min size
## GO_MF    4248            728
## GO_BP    9732           1219
## PW_KEGG   225            201
##
##
## -No of genes in Gene List:
##           input valid duplicate removed converted to entrez
## Gene List 34221 34221             20561               20561
##
##
## -No of hits:
##      input preprocessed
## Hits  6830         4851
##
##
## -Parameters for analysis:
##                minGeneSetSize pValueCutoff pAdjustMethod
## HyperGeo Test 15             0.05         BH
```

```
##
##      minGeneSetSize pValueCutoff pAdjustMethod nPermutations exponent
## GSEA 15             0.05         BH            100           1
##
##
## -Significant gene sets (adjusted p-value< 0.05 ):
##           GO_MF GO_BP PW_KEGG
## HyperGeo   112   192      50
## GSEA        49   165      33
## Both        20    57      17
```

The following enrichment maps (Figure 7 and Figure 8) help visualising the GSEA results, representing gene sets as nodes connected by edges denoting the Jaccard similarity coefficient between two gene sets. Nodes are colored by the sign of the enrichment scores.

In summary, The KRAS signature is enriched for GO terms and pathways associated to:

- Cell cycle checkpoints and cell cycle control: E.g. "cell cycle", "G1/S" transition of the mitotic cell cycle".
- Events that activate signaling pathways that promote cell growth. E.g. "MAPK signaling pathway", "regulation of MAPK cascade", "p53 binding", "Toll-like receptor signaling pathway"
- reorganization of processes that are crucial to cell migration. E.g. "actin binding", "regulation of cell adhesion", "regulation of epithelial to mesenchymal transition", "cell migration".

```
#enrichment map
par(mfrow=c(1,2))
viewEnrichMap(gsca, resultName="GSEA.results",
gscs=c("GO_BP"), allSig=FALSE, ntop=10, gsNameType="term",
displayEdgeLabel=FALSE, layout="layout.fruchterman.reingold")
```

```
## IGRAPH UNW- 10 35 --
## + attr: name (v/c), geneSetSize (v/n), size (v/n), color (v/c), label (v/c),
## | label.dist (v/n), label.cex (v/n), label.font (v/n), label.color (v/c), weight
## | (e/n), color (e/c), width (e/n)
## + edges (vertex names):
##  [1] GO_BP.GO:0000082--GO_BP.GO:0000122 GO_BP.GO:0000082--GO_BP.GO:0001666
##  [3] GO_BP.GO:0000082--GO_BP.GO:0001701 GO_BP.GO:0000122--GO_BP.GO:0001503
##  [5] GO_BP.GO:0000122--GO_BP.GO:0001525 GO_BP.GO:0000122--GO_BP.GO:0001558
##  [7] GO_BP.GO:0000122--GO_BP.GO:0001568 GO_BP.GO:0000122--GO_BP.GO:0001570
##  [9] GO_BP.GO:0000122--GO_BP.GO:0001657 GO_BP.GO:0000122--GO_BP.GO:0001666
## [11] GO_BP.GO:0000122--GO_BP.GO:0001701 GO_BP.GO:0001503--GO_BP.GO:0001525
## + ... omitted several edges
```

```
viewEnrichMap(gsca, resultName="GSEA.results",
gscs=c("GO_MF"), allSig=FALSE, ntop=10, gsNameType="term",
displayEdgeLabel=FALSE, layout="layout.fruchterman.reingold")
```

```
## IGRAPH UNW- 10 27 --
## + attr: name (v/c), geneSetSize (v/n), size (v/n), color (v/c), label (v/c),
## | label.dist (v/n), label.cex (v/n), label.font (v/n), label.color (v/c), weight
## | (e/n), color (e/c), width (e/n)
## + edges (vertex names):
##  [1] GO_MF.GO:0000166--GO_MF.GO:0002039 GO_MF.GO:0000166--GO_MF.GO:0003677
##  [3] GO_MF.GO:0000166--GO_MF.GO:0003700 GO_MF.GO:0000166--GO_MF.GO:0003714
##  [5] GO_MF.GO:0000166--GO_MF.GO:0003723 GO_MF.GO:0000166--GO_MF.GO:0003735
##  [7] GO_MF.GO:0000166--GO_MF.GO:0003743 GO_MF.GO:0000166--GO_MF.GO:0003755
##  [9] GO_MF.GO:0001227--GO_MF.GO:0002039 GO_MF.GO:0001227--GO_MF.GO:0003677
## [11] GO_MF.GO:0001227--GO_MF.GO:0003700 GO_MF.GO:0001227--GO_MF.GO:0003714
```

```
## + ... omitted several edges
```

```
par(mfrow=c(1,1))
viewEnrichMap(gsca, resultName="GSEA.results",
gscs=c("PW_KEGG"), allSig=FALSE, ntop=20, gsNameType="term",
displayEdgeLabel=FALSE, layout="layout.fruchterman.reingold")
```

```
## IGRAPH UNW- 20 62 --
## + attr: name (v/c), geneSetSize (v/n), size (v/n), color (v/c), label (v/c),
## | label.dist (v/n), label.cex (v/n), label.font (v/n), label.color (v/c), weight
## | (e/n), color (e/c), width (e/n)
## + edges (vertex names):
##  [1] PW_KEGG.mmu04144--PW_KEGG.mmu04062 PW_KEGG.mmu04144--PW_KEGG.mmu01100
##  [3] PW_KEGG.mmu04144--PW_KEGG.mmu05016 PW_KEGG.mmu04144--PW_KEGG.mmu04110
##  [5] PW_KEGG.mmu04144--PW_KEGG.mmu04114 PW_KEGG.mmu04144--PW_KEGG.mmu03040
##  [7] PW_KEGG.mmu04144--PW_KEGG.mmu04142 PW_KEGG.mmu04144--PW_KEGG.mmu04010
##  [9] PW_KEGG.mmu04062--PW_KEGG.mmu01100 PW_KEGG.mmu04062--PW_KEGG.mmu05010
## [11] PW_KEGG.mmu04062--PW_KEGG.mmu05016 PW_KEGG.mmu04062--PW_KEGG.mmu04110
## + ... omitted several edges
```

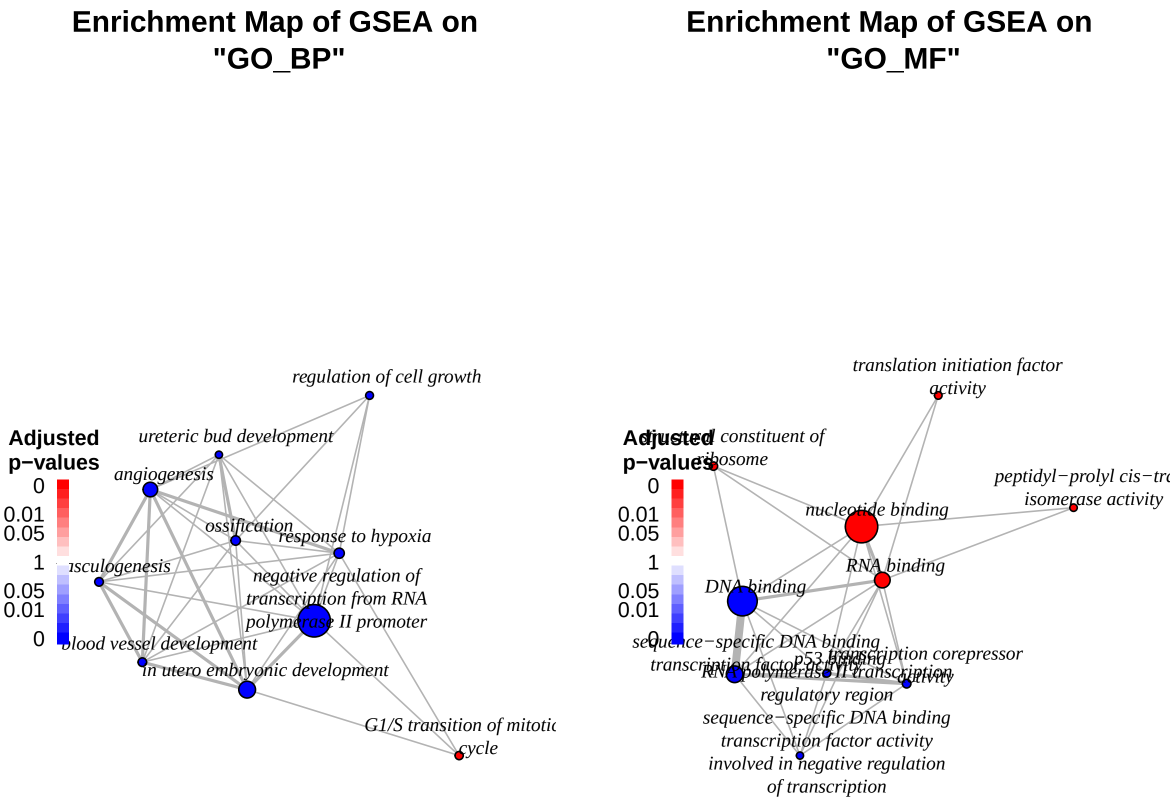

Figure 7: Pathway analysis of KRAS differentially expressed genes. The plot displays the enrichment map obtained by HTSAnalyzeR, for gene set collections obtained from Biological Processes (BP) and Molecular Function (MF) Gene Ontologies.

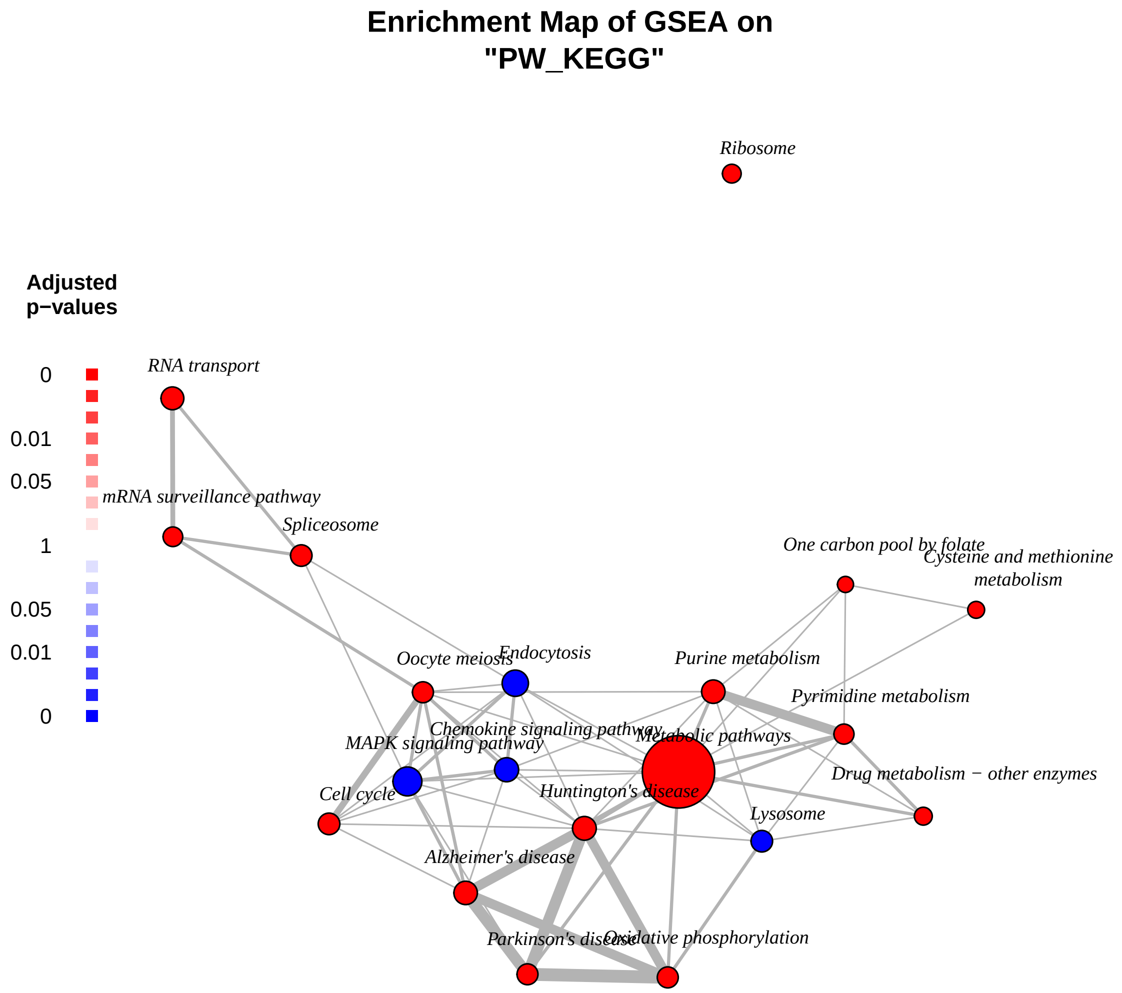

Figure 8: Pathway analysis of KRAS differentially expressed genes. The plot displays the enrichment map obtained by using HTSAnalyzeR, for gene set collections obtained from KEGG GENES database.

The following tables display the results:

	Gene.Set.Term	HyperGeo.Adj.Pvalue	GSEA.A
GO:0000082	G1/S transition of mitotic cell cycle	0.00	
GO:0000122	negative regulation of transcription from RNA polymerase II promoter	0.00	
GO:0001525	angiogenesis	0.00	
GO:0001843	neural tube closure	0.00	
GO:0001934	positive regulation of protein phosphorylation	0.00	
GO:0006260	DNA replication	0.00	
GO:0006281	DNA repair	0.00	
GO:0006351	transcription, DNA-templated	0.00	
GO:0006355	regulation of transcription, DNA-templated	0.00	
GO:0006357	regulation of transcription from RNA polymerase II promoter	0.00	
GO:0006397	mRNA processing	0.00	
GO:0006412	translation	0.00	
GO:0006417	regulation of translation	0.00	
GO:0006730	one-carbon metabolic process	0.03	
GO:0006897	endocytosis	0.00	
GO:0007049	cell cycle	0.00	
GO:0007059	chromosome segregation	0.02	
GO:0007067	mitotic nuclear division	0.00	
GO:0007179	transforming growth factor beta receptor signaling pathway	0.00	
GO:0007507	heart development	0.00	
GO:0008284	positive regulation of cell proliferation	0.01	
GO:0008285	negative regulation of cell proliferation	0.00	
GO:0008360	regulation of cell shape	0.04	
GO:0008380	RNA splicing	0.00	
GO:0010628	positive regulation of gene expression	0.00	
GO:0010718	positive regulation of epithelial to mesenchymal transition	0.03	
GO:0015031	protein transport	0.00	
GO:0016055	Wnt signaling pathway	0.00	
GO:0016126	sterol biosynthetic process	0.02	
GO:0016310	phosphorylation	0.00	
GO:0016477	cell migration	0.02	
GO:0030030	cell projection organization	0.03	
GO:0030324	lung development	0.04	
GO:0030335	positive regulation of cell migration	0.00	
GO:0030336	negative regulation of cell migration	0.02	
GO:0031175	neuron projection development	0.04	
GO:0035458	cellular response to interferon-beta	0.03	
GO:0035556	intracellular signal transduction	0.00	
GO:0043086	negative regulation of catalytic activity	0.03	
GO:0043392	negative regulation of DNA binding	0.04	
GO:0043406	positive regulation of MAP kinase activity	0.05	
GO:0043410	positive regulation of MAPK cascade	0.03	
GO:0043433	negative regulation of sequence-specific DNA binding transcription factor activity	0.00	
GO:0045668	negative regulation of osteoblast differentiation	0.03	
GO:0045669	positive regulation of osteoblast differentiation	0.03	
GO:0045785	positive regulation of cell adhesion	0.05	
GO:0045892	negative regulation of transcription, DNA-templated	0.00	
GO:0045893	positive regulation of transcription, DNA-templated	0.00	
GO:0045944	positive regulation of transcription from RNA polymerase II promoter	0.00	
GO:0048568	embryonic organ development	0.00	
GO:0050731	positive regulation of peptidyl-tyrosine phosphorylation	0.04	
GO:0051028	mRNA transport	0.00	
GO:0051289	protein homotetramerization	0.03	
GO:0051301	cell division	0.00	
GO:0051897	positive regulation of protein kinase B signaling	0.00	
GO:0060348	bone development	0.00	
GO:0072001	renal system development	0.04	

Table 2: Pathway GSEA analysis of KRAS differentially expressed genes. Obtained by with HTSAnalyzeR for gene set collections obtained from Gene Ontolgy Biological Processes (BP). The table shows significantly enriched GO terms (adjusted p-value <0.05) according to both, the GSEA and Hypergeometric test.

	Gene.Set.Term	HyperGeo.Adj.Pvalue	GSEA.Adj.Pvalue
GO:0000166	nucleotide binding	0.00	0.00
GO:0002039	p53 binding	0.00	0.00
GO:0003677	DNA binding	0.00	0.00
GO:0003714	transcription corepressor activity	0.00	0.00
GO:0003723	RNA binding	0.00	0.00
GO:0003779	actin binding	0.00	0.00
GO:0005096	GTPase activator activity	0.00	0.00
GO:0005178	integrin binding	0.03	0.00
GO:0005515	protein binding	0.00	0.00
GO:0008134	transcription factor binding	0.00	0.00
GO:0008270	zinc ion binding	0.00	0.00
GO:0016874	ligase activity	0.00	0.00
GO:0019901	protein kinase binding	0.00	0.00
GO:0019903	protein phosphatase binding	0.00	0.00
GO:0031625	ubiquitin protein ligase binding	0.00	0.00
GO:0032403	protein complex binding	0.00	0.00
GO:0044212	transcription regulatory region DNA binding	0.01	0.00
GO:0044822	poly(A) RNA binding	0.00	0.00
GO:0046872	metal ion binding	0.00	0.00
GO:0050660	flavin adenine dinucleotide binding	0.00	0.00

Table 3: Pathway analysis of KRAS differentially expressed genes. Obtained by with HTSAnalyzeR for gene set collections obtained from Gene Ontology Molecular Function (MF). The table shows significantly enriched GO terms (adjusted p-value <0.05) according to both, the GSEA and Hypergeometric test.

	Gene.Set.Term	HyperGeo.Adj.Pvalue	GSEA.Adj.Pvalue
mmu04144	Endocytosis	0.00	0.00
mmu01100	Metabolic pathways	0.00	0.00
mmu04110	Cell cycle	0.00	0.00
mmu04114	Oocyte meiosis	0.02	0.00
mmu03015	mRNA surveillance pathway	0.00	0.00
mmu03040	Spliceosome	0.00	0.00
mmu03013	RNA transport	0.00	0.00
mmu00230	Purine metabolism	0.03	0.00
mmu00240	Pyrimidine metabolism	0.00	0.00
mmu04142	Lysosome	0.00	0.00
mmu04010	MAPK signaling pathway	0.00	0.00
mmu05145	Toxoplasmosis	0.00	0.00
mmu03030	DNA replication	0.00	0.00
mmu03420	Nucleotide excision repair	0.00	0.00
mmu04620	Toll-like receptor signaling pathway	0.01	0.00
mmu04380	Osteoclast differentiation	0.00	0.00
mmu00534	Glycosaminoglycan biosynthesis - heparan sulfate	0.01	0.00

Table 4: Pathway GSEA analysis of KRAS differentially expressed genes. Obtained by with HTSAnalyzeR for gene set collections obtained from KEGG GENES database. The table shows significantly enriched KEGG pathways (adjusted p-value <0.05) according to both, the GSEA and Hypergeometric test.

8 Finding Master Regulators of KRAS

Master regulators of KRAS were identified with the `msviper` functionality of the *viper* (Virtual Inference of Protein-activity by Enriched Regulon analysis) package.

8.1 KRAS null model

To account for the correlation structure between genes (gene expression is not statistically independent between genes), the null model for `msviper` is defined by using a set of signatures obtained after permuting the samples at random. The function `limmaNull` performs such process by shuffling the samples at random. `limmaNull` uses Limma's empirical Bayes moderated t-test to produce a numerical matrix of z-scores, with genes in rows and permutation iterations in columns, than can be used as null model for the msVIPER analysis.

```
#null models
nullmodel <- limmaNull(KRASgexp, KRAStargets, KRASannotTable, KRASannotation, nPermutations=1000)
```

Due to large computation time required to compute the null model, we provide the R object with the pre-computed null model for the KRAS signature:

```
load("data/PreComputedKRASnullmodel.RData")
```

8.2 Preparing network data

To run *viper* analysis, We firstly converted the `TPC-class` object to a Regulation object, and re-annotated names of factors (add gene symbol to Entrez IDs):

```
regul <- TFuse2regulon(combinedNetwork[["fusedNet"]])
```

8.3 msVIPER analysis

The msVIPER analysis is performed by the `msviper` function. It requires the KRAS signature, the PDAC network object and the null model as arguments, and produces an object of class "msVIPER", containing the estimated enrichment for each regulon, including the Normalized Enrichment Score (NES) and p-values, as output.

```
library(viper)
mrs <- msviper(KRASsig[["statistic"]], regul, nullmodel, verbose =FALSE)
```

The top identified master regulators are shown in in Figure 9:

```
p1 <- sort(mrs$es$p.value)
MRs <- names(p1)[p1 < 0.01]
plot(mrs, length(MRs))
```

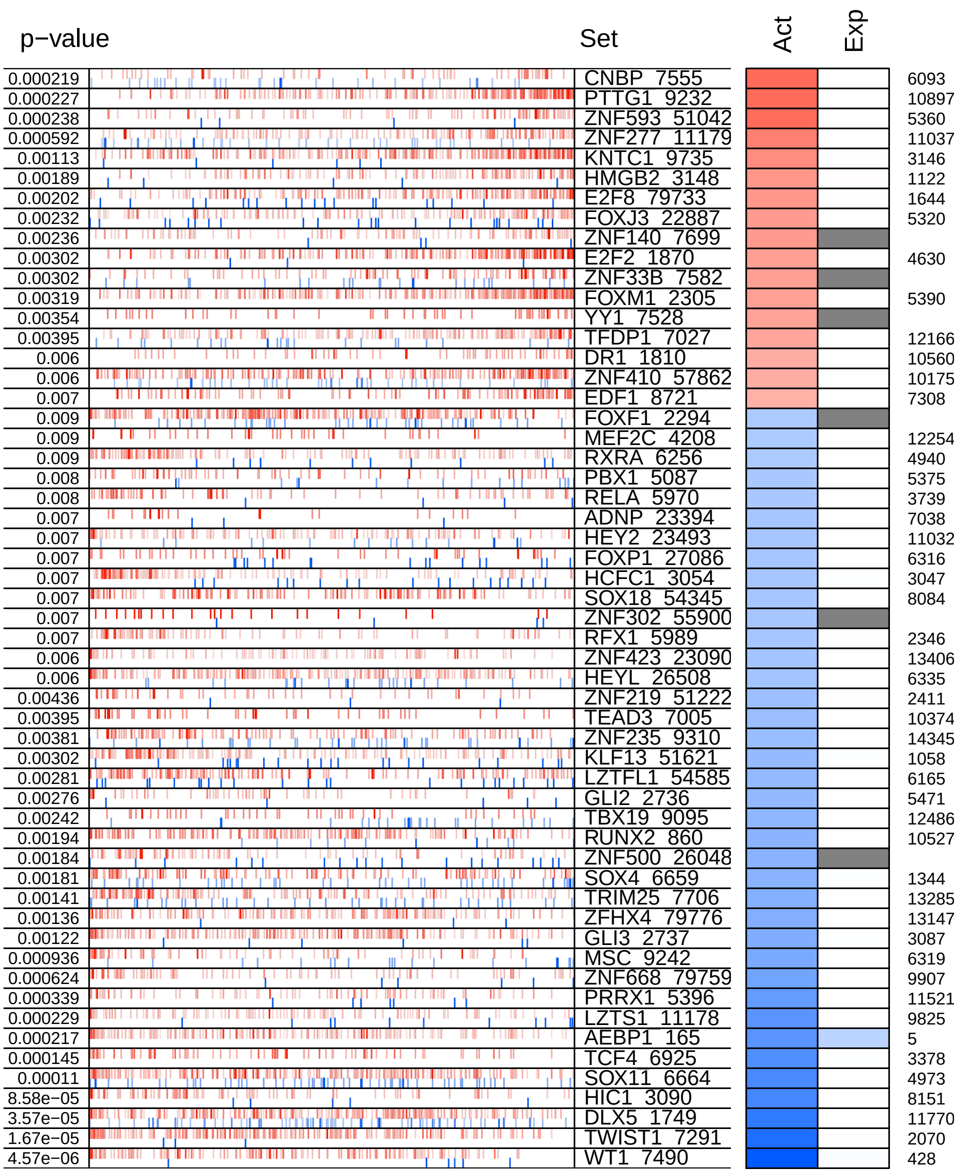

Figure 9: msVIPER plot showing the projection of the negative (repressed, shown in blue color) and positive (activated, shown in red color) targets for each TF, as inferred by RTN and partial-correlation analysis when reverse engineering the regulatory network (vertical lines resembling a bar-code), on the KRAS gene-expression signature. The KRAS signature is represented in the-axis, where the genes were rank-sorted based on the t-statistic for the DEG test (limma analysis) from the one most down-regulated to the one most upregulated in the 'Cre' condition compared to "Mock". The two-columns heatmap displayed on the right side of the figure shows the inferred differential activity (first column) and differential expression (second column), with the rank of the displayed genes in the KRAS signature (shown all the way to the right).

9 Network comparisons

9.1 Edge correlation between pairs of networks

We compared the derived regulons between pairs of networks. For each pairwise comparison, we computed two different quantitative metrics regulon-wise:

- We compared the vectors of edge weights and determined if they were significantly correlated by computing the Pearson correlation coefficient (Figure 10)
- We tested the overlap between the inferred target genes (significant edges defined by q-value <0.05) using Fisher's exact test (Figure 11)

```
compareRes <- compareallNetworks(allNetworks, combinedNetwork[["fusedNet"]])
```

```
CorPerRegDist_all <- lapply(compareRes, function(x) unlist(x[["CorPerReg_all"]]))
CorPerRegDist_sig <- lapply(compareRes, function(x) unlist(x[["CorPerReg_sig"]]))
FETdist <- lapply(compareRes, "[[", "FETPerReg")

#compare correlation coefficient scores per Regulon
library(ggplot2)
library(reshape2)
all <- melt(do.call("cbind",CorPerRegDist_all))
all$type <- "all"
sig <- melt(do.call("cbind",CorPerRegDist_sig))
sig$type <- "sig"
a <- rbind(all,sig)
ggplot(a, aes(x=Var2, y=value, fill=type)) +
  geom_boxplot() + coord_flip() + xlab("network pairs") +
  ylab("Edge Pearson correlation coeficient per regulon") +
  ggtitle("Edge correlation")
#compare regulon overlap
a <- melt(do.call("cbind",FETdist))
ggplot(a, aes(x=Var2, y=value)) + ylim(0,10) +
  geom_boxplot() + coord_flip() + xlab("network pairs") +
  ylab("-log10(p-value) Fisher Exact Test") +
  xlab("") + ggtitle("Regulon overlap test")
```

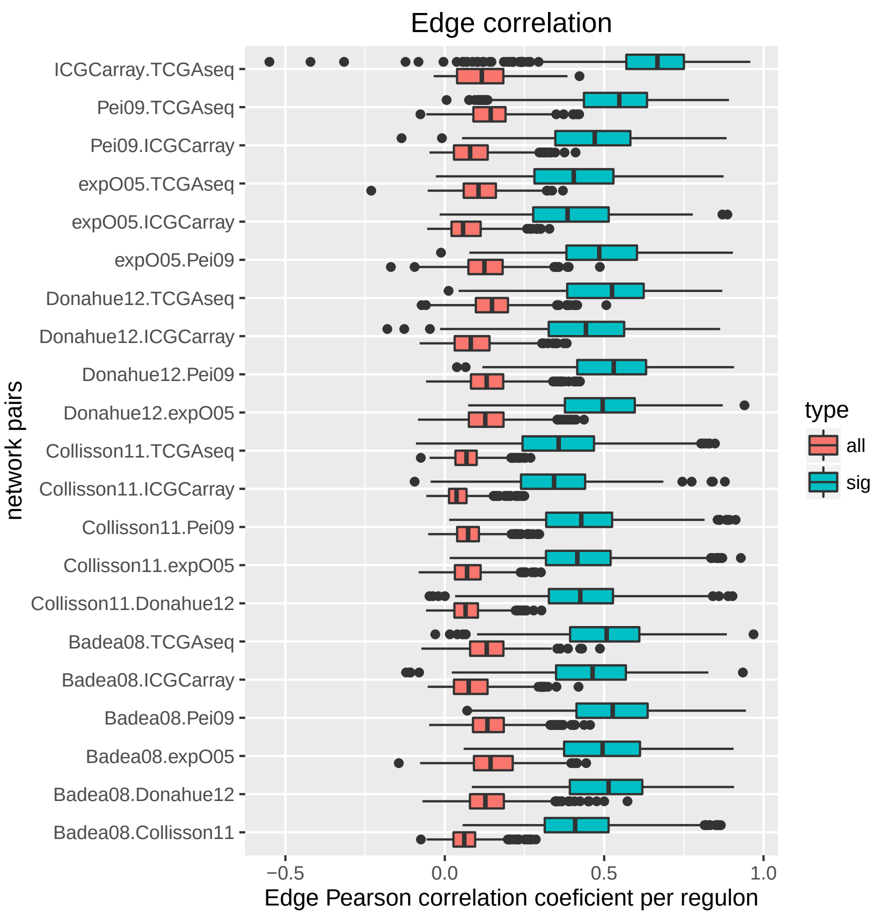

Figure 10: Edge weight correlation between pairs of networks. For each regulon, Pearson correlation coefficients were computing by comparing the edge z-scores between pairs of networks. The boxplots display the distribution of regulon Pearson correlation coefficients when considering all edges (orange), or when considering only the edges with significant FDR in both networks (q-value <0.05) (blue). The correlation between edge weights increases when considering only significant edges.

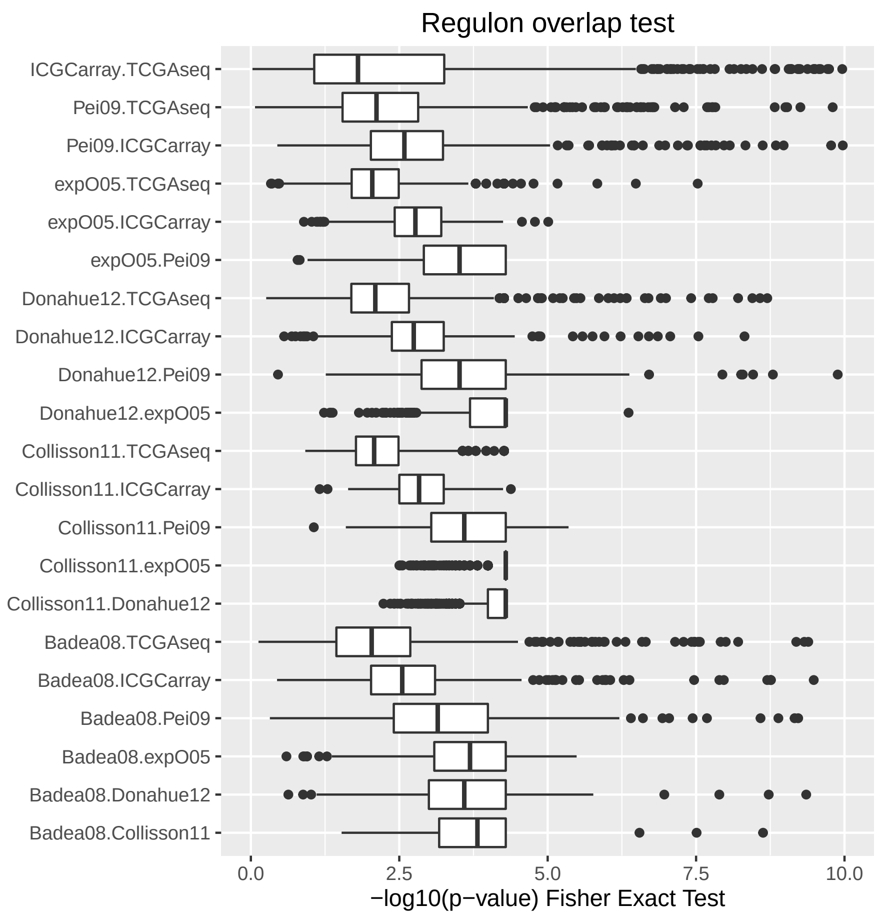

Figure 11: Regulon overlap between pairs of networks. Regulon overlap was computed by considering the edges with significant FDR (q-value <0.05) and the p-value for the degree of overlap calculated by a Hypergeometric test.

9.2 Network activity comparison

Since our goal has been to identify transcriptional regulators that drive the KRAS-related phenotype, a key question is whether individual networks yield comparable results to the combined network. We ran msVIPER for each individual network and compared the vectors of activity obtained from the combined network and each one of the individual networks to determine if they were significantly correlated (Figure 12 and Figure 13).

```
#compare network activity
result <- getActivity(allNetworks, combinedNetwork[["fusedNet"]], KRASsig, nullmodel)
pvalue.matrix <- result[["pvalue.matrix"]]
NES.matrix <- result[["NES.matrix"]]
```

The figure below shows, for each one of the 55 master regulators, how the normalized enrichment scores (NES) and the associated p-values obtained by msVIPER analysis are in good agreement for all individual networks when compared to the combined network. As expected, networks with higher number of samples (ICGC and TCGA cohorts) are in better agreement with the combined network result. Nonetheless, the power of the analysis was improved by combining the seven different cohorts:

```
pheatmap(as.matrix(pvalue.matrix[rownames(pvalue.matrix) %in% MRs,]),
         scale="none",cluster_rows=F, cluster_cols=F)
pheatmap(as.matrix(NES.matrix[rownames(NES.matrix) %in% MRs,]),
```

```
         scale="none",cluster_rows=F, cluster_cols=F)
```

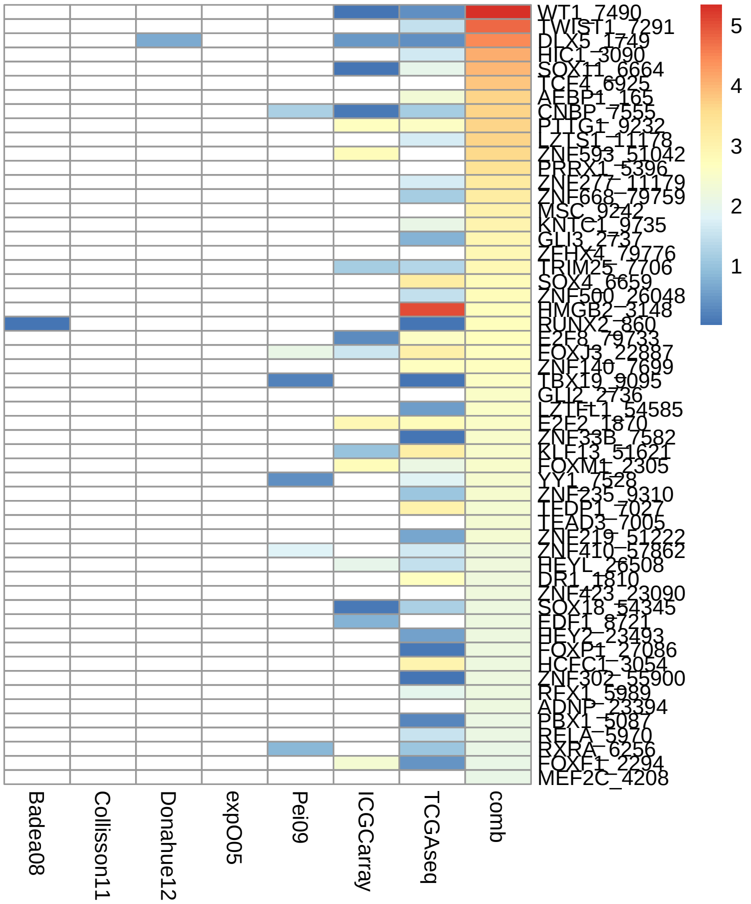

Figure 12: Agreement of p-values obtained by msVIPER analysis for the top 55 master regulators in all seven individual networks and in the combined fused Network (comb). Networks with higher number of samples (ICGC and TCGA cohorts) are in better agreement with the combined network result. -log10(p-values) are colored from the smallest p-values (red) to the largest (blue). White cells in the heatmap correspond to missing values, these exist for master regulators with no inferred edges at a FDR <0.05.

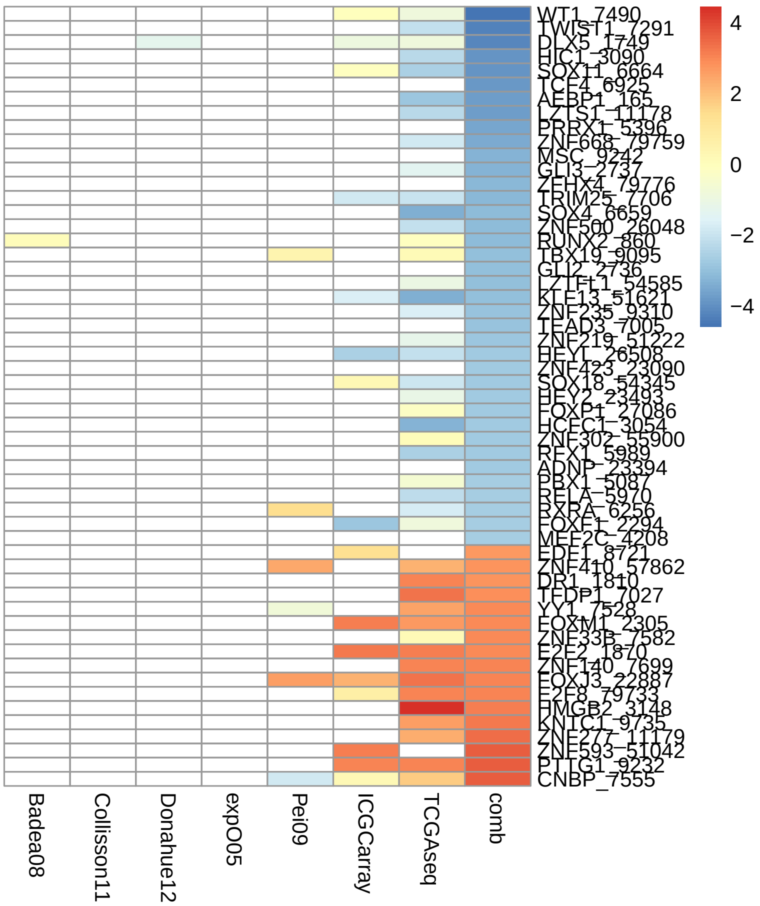

Figure 13: Agreement of normalized enrichment scores (NES) obtained by msVIPER analysis for the top 55 master regulators in all seven individual networks and in the combined fused Network (comb). Networks with higher number of samples (ICGC and TCGA cohorts) are in better agreement with the combined network result. Negative NES scores are colored in blue and positive NES scores in red. White cells in the heatmap correspond to missing values, these exist for master regulators with no inferred edges at a FDR <0.05.

10 Community search algorithm to identify core pathways

To identify communities within the top 55 master regulators, we used the Fastgreedy algorithm implemented in the R function `fastgreedy.community` of the *igraph* package.This function tries to find dense subgraph, also called communities in graphs via directly optimizing a modularity score using the Clauset-Newman-Moore algorithm (Clauset et al., 2004).

The analysis reaveled 3 major communities, corresponding to 27 Regulators and their target genes.

```
res <- plotcom(combinedNetwork[["fusedNet"]],MRs)
names(res) <- c("CellCycle","Hedgehog","Notch")
#save(res,p1,MRs, mrs, file="data/comunities.Rda")
```

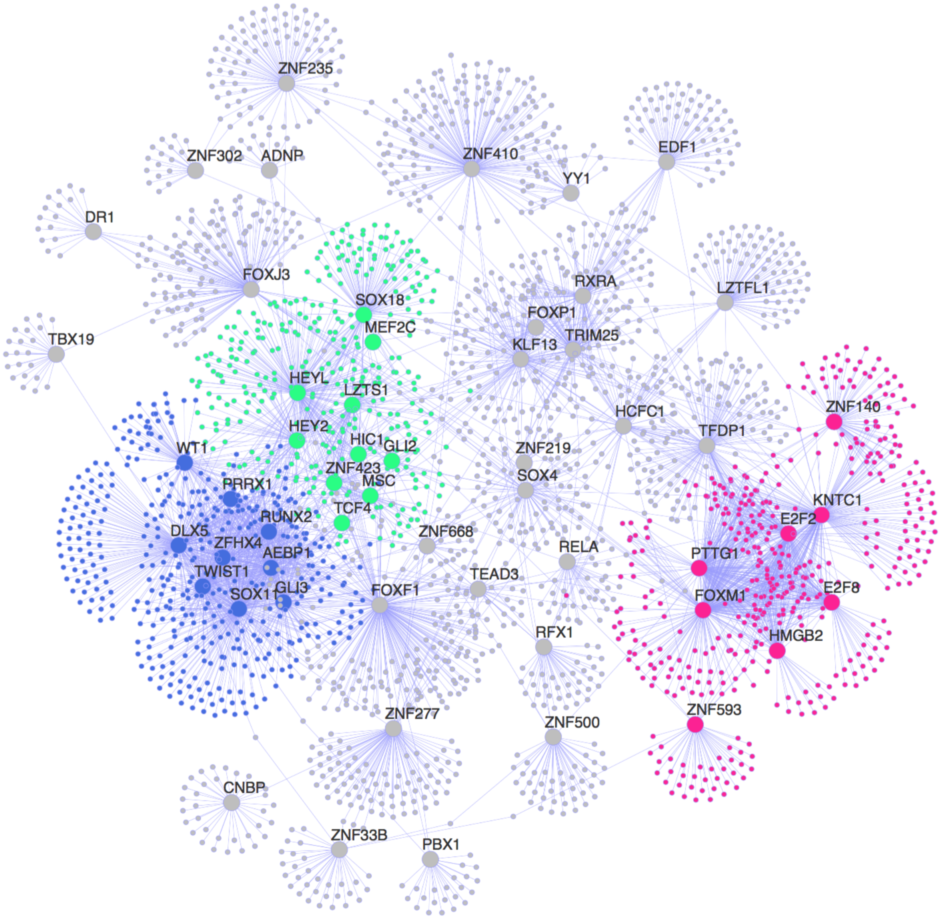

Figure 14: Visual representation of MRs identified with msVIPER analysis (p <0.01). The nodes in the networks represent the 55 master regulators (large dots) and the corresponding inferred targets (smaller dots). The edges in the network represent the regulatory relationship between regulators and the inferred targets. The colors highlight the community structure of the network identified by via greedy optimization of modularity. The three groups of nodes correspond to a total of 27 master regulators and represent three distinct disease processes enriched for cell cycle (pink), Hedgehog/Wnt signaling (blue) and Notch signaling (green) pathways.

10.1 Pathway analysis of the identified TF signatures

In the following section, we performed a pathway analysis of these 3 communities using the R package *clusterProfiler* to identify predominant biological pathways enriched in the three gene lists. Pathway analysis was performed using pathway annotations provided by the Kyoto Encyclopedia of Genes and Genomes (KEGG) which is available via the *KEGG.db* and *clusterProfiler* R packages.

```
geneids <- lapply(res, getentrez)
universe <- getentrez(rownames(combinedNetwork[["fusedNet"]]))

#for barplot
kk <- lapply(geneids, Keggplot, universe=universe, pvalueCutoff=0.01)
kk_reactome <- lapply(geneids, reactomePAplot, universe=universe, pvalueCutoff=0.01)

#fpr compare cluster plot
names(geneids) <- c("group1","group2","group3")
```

```
ck <- compareCluster(geneids, fun='enrichKEGG', organism="human")
```

Due to the large computation time we provide the pathway analysis result as an R object

```
load("data/PreComputedMRspathwayAnlaysis.RData")
```

We visualize the enrichment result in a barplot. The barplot shows all enriched categories in each group. The x-axis represents the enrichment result p-value -log10(p.adjust) after p-value adjustment. P-value adjustment was performed with the Benjamini Hochberg (FDR) method.

```
multiplot(barplotkk(kk[[1]], main="group1"),
          barplotkk(kk[[2]], main="group2"),
          barplotkk(kk[[3]], main="group3"),cols=3)
```

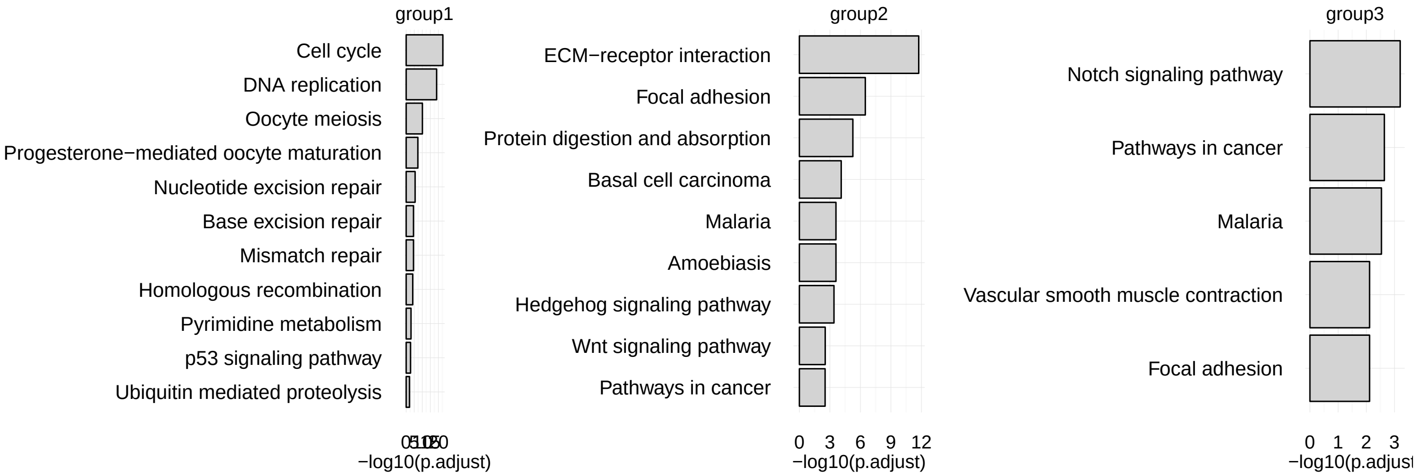

Figure 15: KEGG Pathway enrichment analysis of the genes in each of the three disease processes. The barplot shows all enriched categories at a adjusted p-value <0.01.

There are clear differences in the most enriched categories per group. For example:

- Group1 is enriched in genes involved with Cell Cycle and DNA-replication controlling functions.
- Group2 is enriched in ECMreceptor interaction, Focal adhesion and "Hedgehog/Wnt" signaling genes.
- Group3 is enriched in Focal adhesion and Notch signaling genes.

Group2 and Group3 show some similar enriched pathways, such as "Focal adhesion" and "Pathways in cancer". The following "grouped" plot shows more clearly the different enriched pathways between groups. The total number of identified genes in each category are provided as numbers in parentheses in the x-axis. The dots in the plot are color-coded based on their corresponding FDR adjusted p-values. Color gradient ranging from red to blue correspond to in order of increasing p-values. That is, red indicate low p-values (high enrichment), and blue indicate high p-values (low enrichment). The size of the dots correspond to the "Gene Ratio" which corresponds to the proportion of genes from each group in each KEGG pathway.

```
plot(ck, showCategory=10)
```

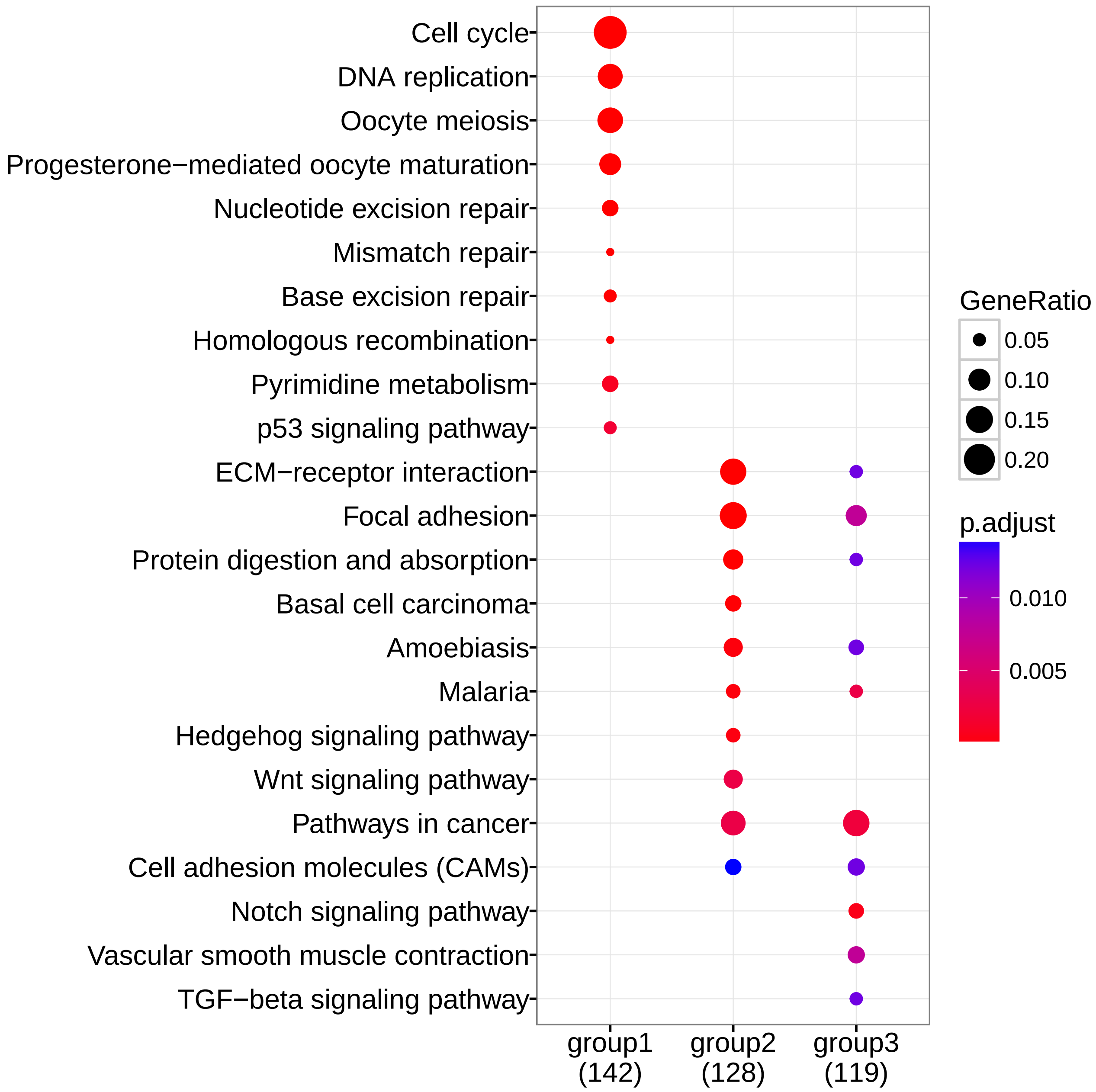

Figure 16: KEGG Pathway enrichment analysis of the genes in each of the three disease processes. The plot shows all enriched categories at FDR adjusted p-value <0.01 per group. The total number of identified genes in each group are provided as numbers in parentheses in the x-axis. The dots in the plot are color-coded based on their corresponding FDR adjusted p-values. The size of the dots correspond to the "Gene Ratio" which corresponds to the proportion of genes from each group in each category of KEGG pathways.

The following tables display the results:

	group	ID	Description	GeneRatio	p.adjust
1	group 1	hsa04110	Cell cycle	32/142	1.54096565401427e-23
2	group 1	hsa03030	DNA replication	18/142	1.17912360338892e-19
3	group 1	hsa04114	Oocyte meiosis	19/142	7.81787448584938e-11
4	group 1	hsa04914	Progesterone-mediated oocyte maturation	14/142	5.2523271134736e-08
5	group 1	hsa03420	Nucleotide excision repair	9/142	2.69717153969186e-06
6	group 1	hsa03430	Mismatch repair	6/142	2.71714411268614e-05
7	group 1	hsa03410	Base excision repair	7/142	2.71714411268614e-05
8	group 1	hsa03440	Homologous recombination	6/142	8.02522856389859e-05
9	group 1	hsa00240	Pyrimidine metabolism	9/142	0.000970615801411671
10	group 1	hsa04115	p53 signaling pathway	7/142	0.00188876769185718
11	group 1	hsa04120	Ubiquitin mediated proteolysis	9/142	0.00834288164238199
12					
13	group 2	hsa04512	ECM-receptor interaction	18/128	1.837262015187e-12
14	group 2	hsa04510	Focal adhesion	19/128	3.2772634651433e-07
15	group 2	hsa04974	Protein digestion and absorption	11/128	5.49371605101094e-06
16	group 2	hsa05217	Basal cell carcinoma	8/128	7.70129771227645e-05
17	group 2	hsa05146	Amoebiasis	10/128	0.000252693723386758
18	group 2	hsa05144	Malaria	7/128	0.000252693723386758
19	group 2	hsa04340	Hedgehog signaling pathway	7/128	0.00039651714375963
20	group 2	hsa04310	Wnt signaling pathway	10/128	0.00288925042984484
21	group 2	hsa05200	Pathways in cancer	16/128	0.00295419339304143
22					
23	group 3	hsa04330	Notch signaling pathway	7/119	0.000627481776113118
24	group 3	hsa05200	Pathways in cancer	17/119	0.00228593307609322
25	group 3	hsa05144	Malaria	6/119	0.00290115048416437
26	group 3	hsa04510	Focal adhesion	11/119	0.00765737073009146
27	group 3	hsa04270	Vascular smooth muscle contraction	8/119	0.00765737073009146

Table 5: KEGG Pathway enrichment analysis of the genes in each of the three disease processes. Obtained by with ClusterProfiler for gene set collections obtained from KEGG database. The table shows significantly enriched KEGG pathways (FDR adjusted p-value <0.01) according the Hypergeometric test.

Part III

Characterizing Pancreatic Cancer subtypes

11 Subtype analysis

11.1 PDAC subtypes for the TCGA and ICGC cohorts

In order to detect the similarity between tumor samples based on the activity of the 27 master regulators we used the methodology developed be Alvarez et al 2016. We took the gene expression matrix for the TCGA and ICGC cohorts and applied the function *viper* from the *viper* package to perform a single-sample analysis and compute a matrix with activity scores for each one of the 27 regulators. The matrix of 27 activity scores for each tumor sample was then used to compute the similarity of between different tumor samples.

```
#run
TFs2cluster <- unlist(lapply(res, function(x) {x[x %in% MRs]}), use.names=F)

TCGAact <- runViper(TCGAseq.Gexp,net=combinedNetwork[["fusedNet"]], MR=TFs2cluster)
TCGA.distance <- signatureDistance(TCGAact, scale=F)

ICGCact <- runViper(ICGCarray.Gexp,net=combinedNetwork[["fusedNet"]], MR=TFs2cluster)
ICGC.distance <- signatureDistance(ICGCact, scale=F)
```

11.2 Evaluation of the best number of clusters

The "signatureDistance" class object can be used to perform cluster analysis of the samples.

```
par(mfrow=c(1,2))
layout(rbind(c(4,3,8,7),c(2,1,6,5)),
       widths = c(1,2,1,2), heights = c(1,2), respect = FALSE)

heatmap(as.matrix(as.dist(TCGA.distance)), Rowv = as.dendrogram(hclust(as.dist(TCGA.distance),
method = "average")), symm = T, labRow="",labCol="", main="TCGA")
heatmap(as.matrix(as.dist(ICGC.distance)), Rowv = as.dendrogram(hclust(as.dist(ICGC.distance),
method = "average")), symm = T, labRow="",labCol="", main="ICGC")
```

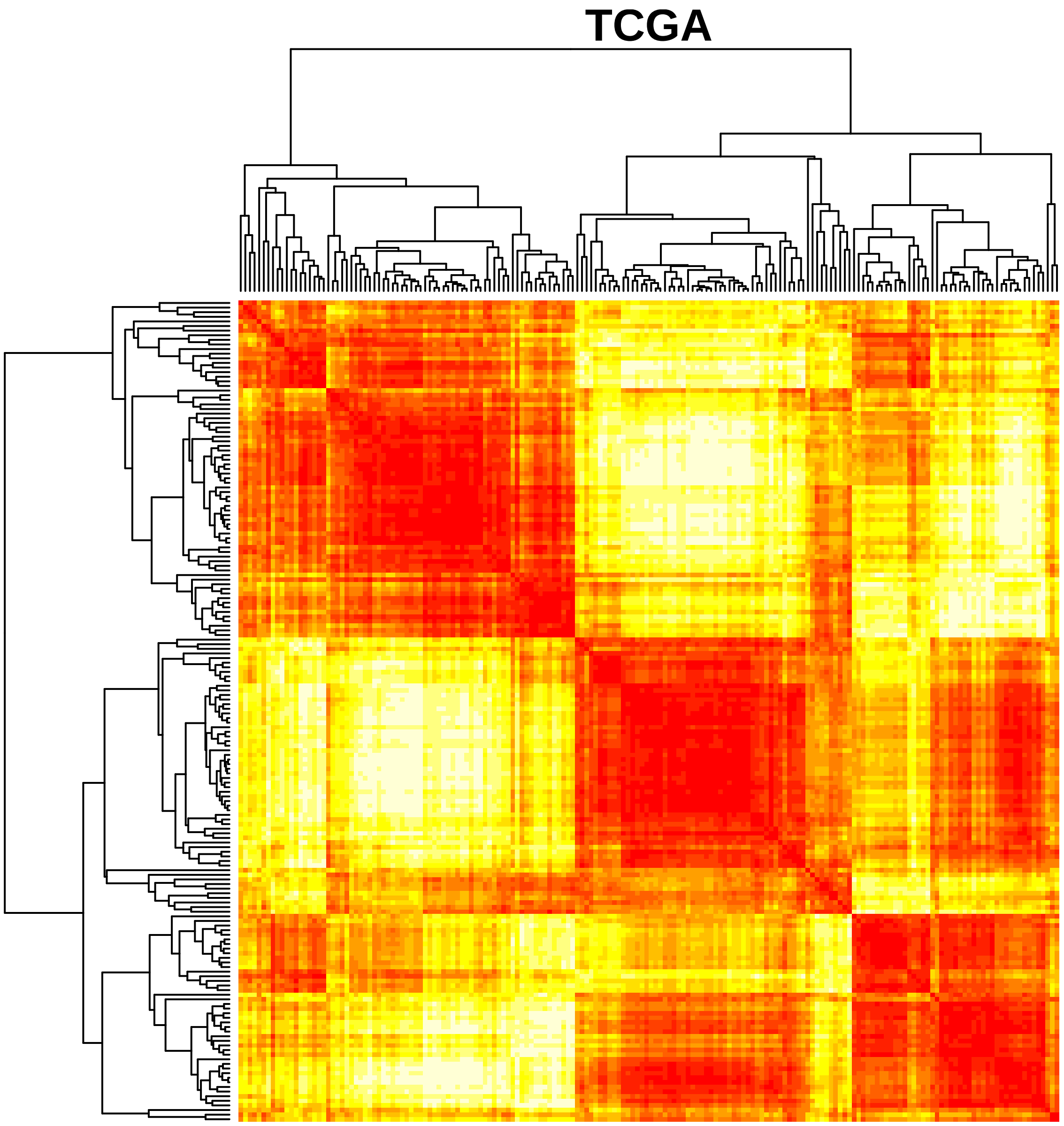

Figure 17: Heatmap showing the similarity between the samples in the TCGA cohort as measured by 'signature distance' between the VIPER-inferred transcriptional regulator's activity profiles.

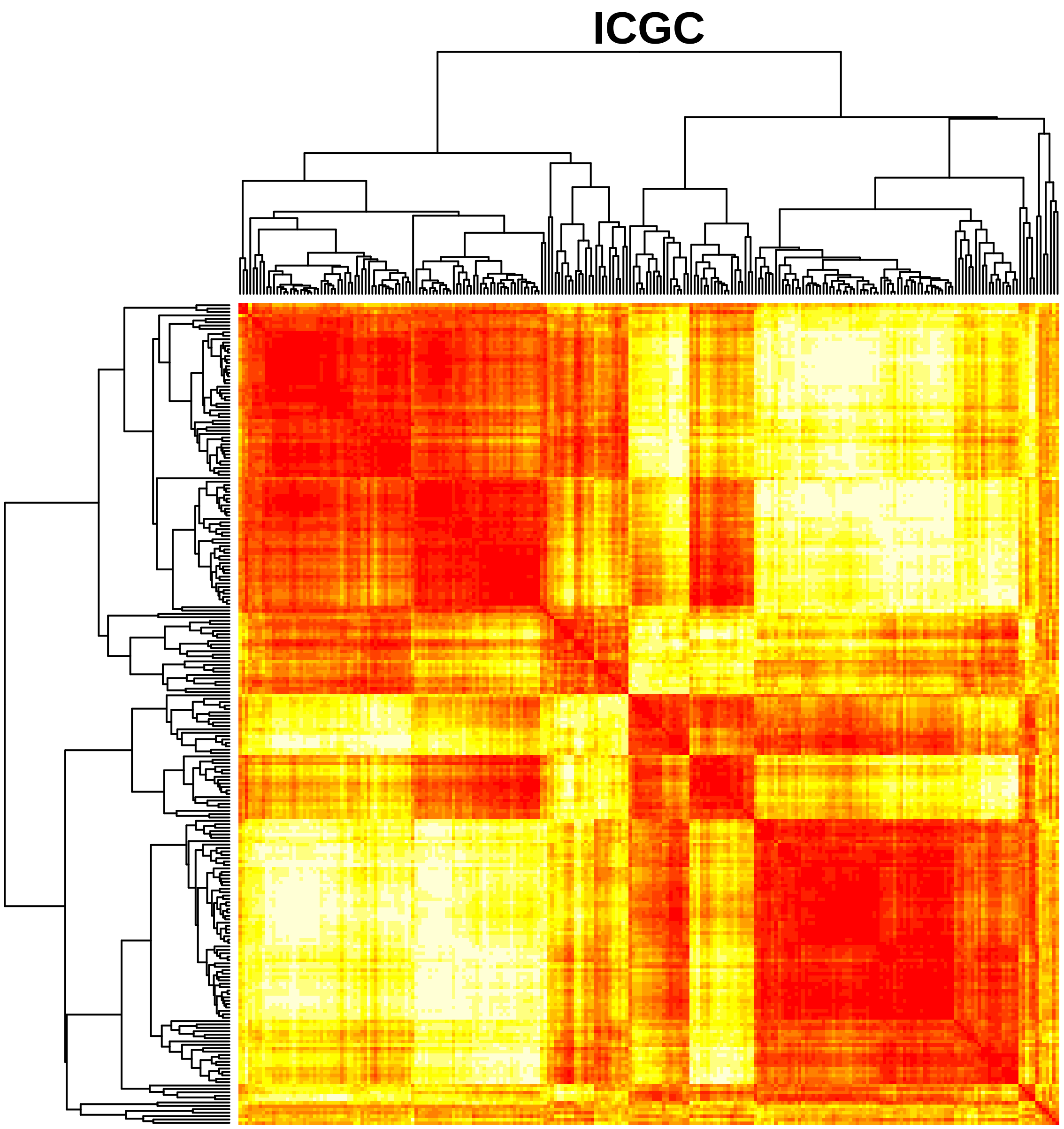

Figure 18: Heatmap showing the similarity between the samples in the ICGC cohort as measured by 'signature distance' distance between the MRs activity profiles

A high average silhouette widths were obtained for three clusters. Therefore we continue the analysis using three clusters as the optimum number of clusters.

```
par(mfrow=c(1,2))
cormat <- scale(TCGA.distance)
distance <- as.dist(cormat)
fit1 <- hclust(as.dist(cormat),method = "average")
```

```
avsi <- sapply(2:8, function(i) {a <- silhouette(cutree(fit1, i), distance); mean(a[,3])})
plot(2:8, avsi, type="b", xlab="Number of Clusters", ylab="Average silhouette width", main="TCGA")

cormat <- scale(ICGC.distance)
distance <- as.dist(cormat)
fit2 <- hclust(as.dist(cormat),method = "average")
avsi <- sapply(2:8, function(i) {a <- silhouette(cutree(fit2, i), distance); mean(a[,3])})
plot(2:8, avsi, type="b", xlab="Number of Clusters", ylab="Average silhouette width", main="ICGC")
```

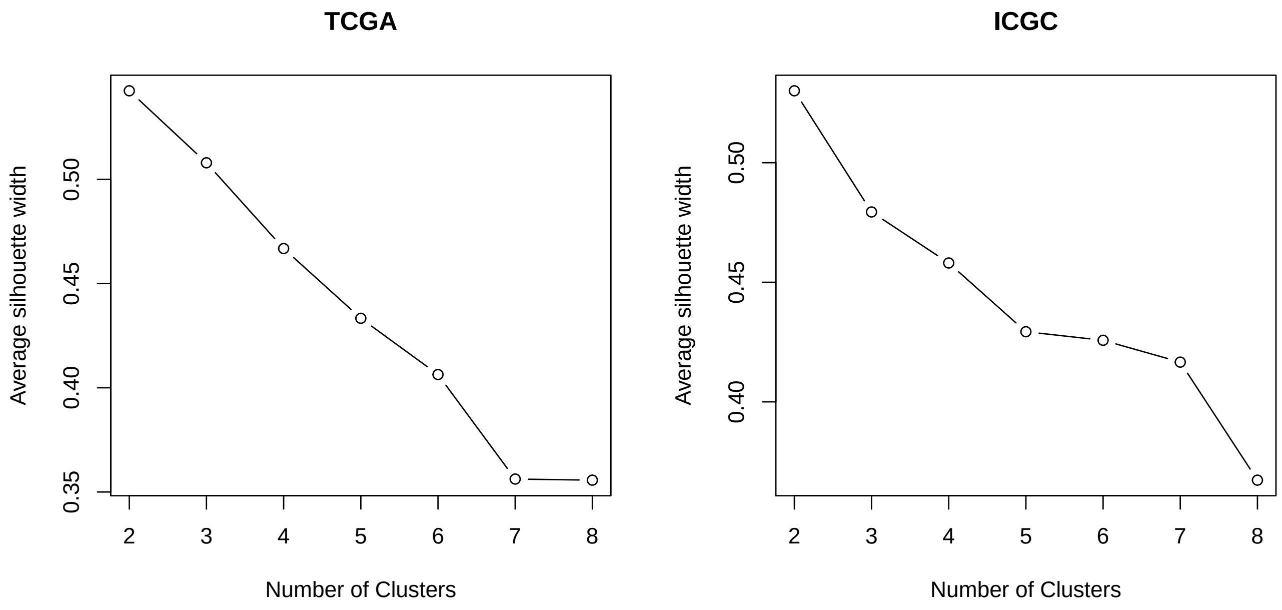


```
#get the groups
tgroupsTCGA <- cutree(fit1, k=3)
tgroupsICGC <- cutree(fit2, k=3)

#fixnames
bcr_aliquot_uuid = sapply(names(tgroupsTCGA),
function(x) {paste(strsplit(strsplit(x, "UNCID")[[1]][2],
                            "\\.")[[1]][2:6],collapse="-")})
names(tgroupsTCGA) <- as.character(bcr_aliquot_uuid)

#save(tgroupsICGC, tgroupsTCGA, file="data/tgroups.Rda")
```

```
COL = colorRampPalette(rev(brewer.pal(n = 7, name ="RdGy")))(100)

#ICGC
tgroups <- factor(tgroupsICGC)
levels(tgroups) <- c("CellCycle","Notch","Hedgehog")
act <- ICGCact
tgroups <- sort(tgroups)
annotation_colors <- list("groups"=c("CellCycle"="deeppink",
                                     "Hedgehog"="royalblue", "Notch"="springgreen"))
pheatmap(act[,names(tgroups)], scale="row", cluster_cols = F,
         annotation_col = data.frame("groups"=factor(tgroups)),
         annotation_colors=annotation_colors,
```

```
          show_colnames=FALSE, color = COL)
```

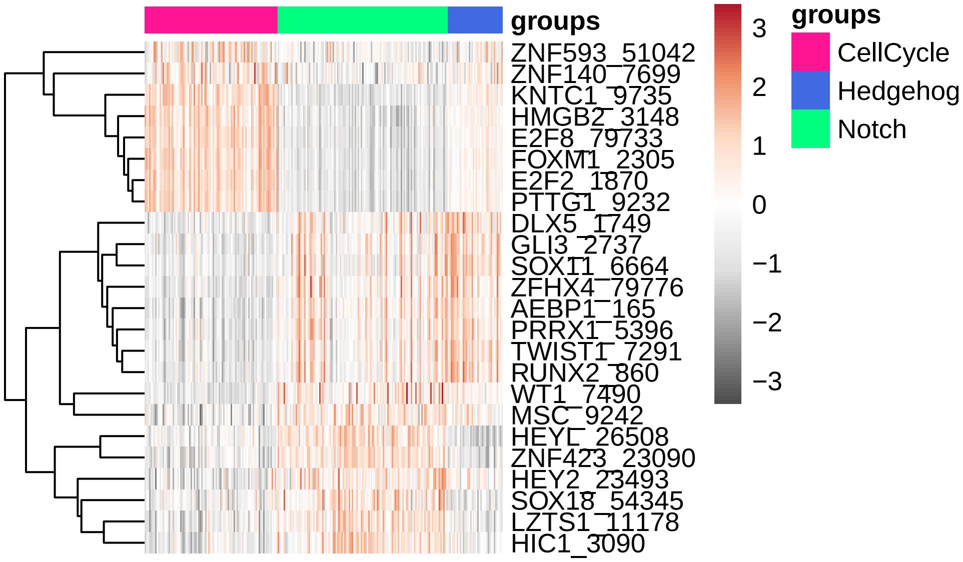


```
#TCGA
#fixnames
bcr_aliquot_uuid = sapply(colnames(TCGAact),
function(x) {paste(strsplit(strsplit(x, "UNCID")[[1]][2],
                               "\\.")[[1]][2:6],collapse="-")})
colnames(TCGAact) <- as.character(bcr_aliquot_uuid)
rm(bcr_aliquot_uuid)

act <- TCGAact
tgroups <- factor(tgroupsTCGA)
levels(tgroups) <- c("Hedgehog","CellCycle","Notch")
tgroups <- sort(tgroups)
annotation_colors <- list("groups"=c("CellCycle"="deeppink","Hedgehog"="royalblue", "Notch"="springgreen")
pheatmap(act[,names(tgroups)], scale="row", cluster_cols = F,
          annotation_col = data.frame("groups"=factor(tgroups)),
          annotation_colors=annotation_colors,
          show_colnames=FALSE,
          color = COL)
```

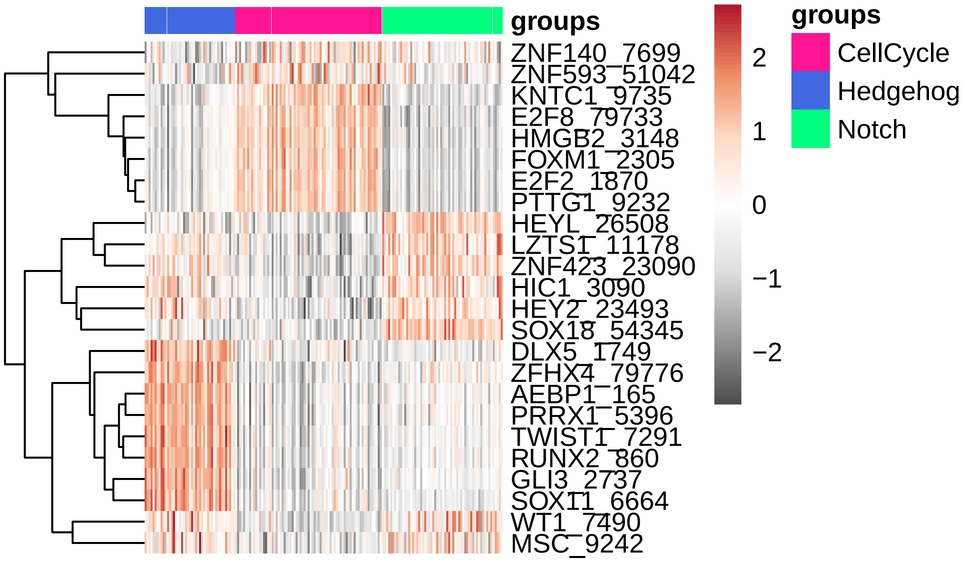

11.3 Comparing the different subtypes with previous datasets

We used the classification obtained by Bailey et al., 2016 to further evaluate to which classes are enriched in the three regulatory programs.

```
tgroups <- factor(tgroupsICGC)
levels(tgroups) <- c("CellCycle","Notch","Hedgehog")

a <- data.frame("BaileyClasses"=clinicalICGC$ICGCsubtypes, "group"=tgroups[match(rownames(clinicalICGC),na
data2plot <- melt(table(a))

bp1 <- ggplot(data2plot, aes(x=group, y=value, fill=BaileyClasses)) +
  geom_bar(stat="identity", color="black", position = "fill") + xlab("") + ylab("Proportion of samples") +
  theme_minimal() + theme(axis.text.y = element_text(size=15),
  axis.text.x = element_text(size=15),  axis.title=element_text(size=15))

#bp2 <- ggplot(data2plot, aes(x=group, y=value, fill=BaileyClasses)) +
#  geom_bar(stat="identity", color="black") + xlab("") + ylab("Number of #samples") +
#  theme_minimal() + theme(axis.text.y = element_text(size=15),
#  axis.text.x = element_text(size=15),  axis.title=element_text(size=15))
#multiplot(bp1, bp2)

plot(bp1)
```

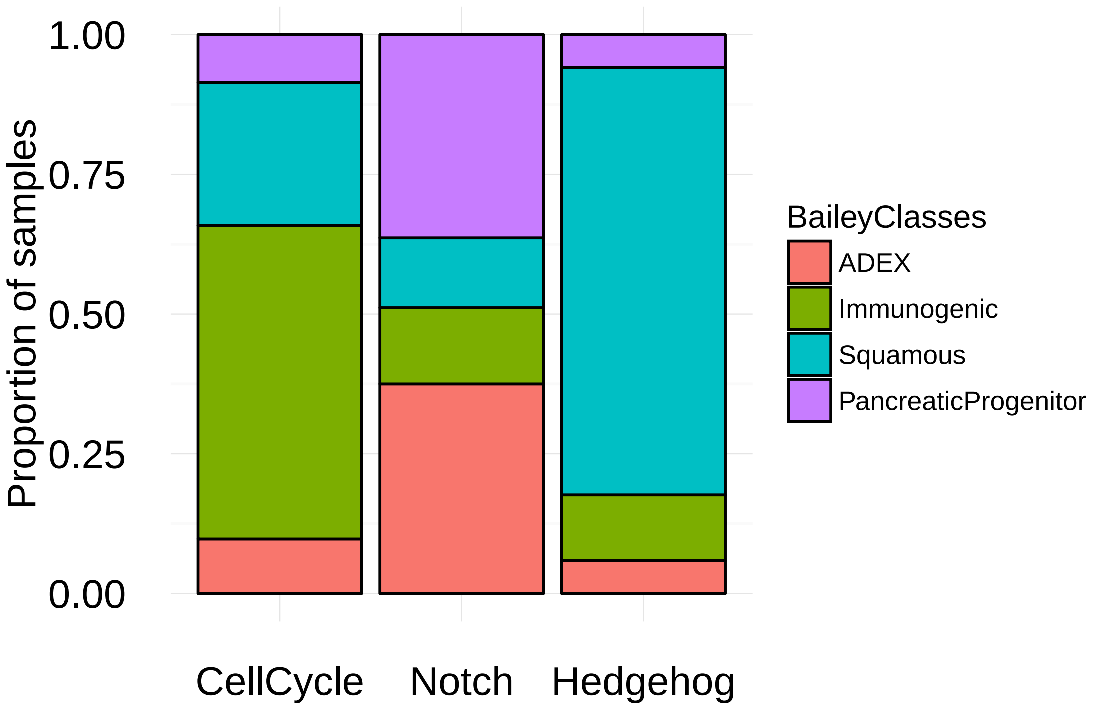

Figure 19: Comparison of disease regulatory processes identified in this study with the tumor subtypes identified previously in Bailey et al., 2016. The Hedgehog/Wnt process is over-represented by samples from the Squamous subtype (p-value=1.1e-8), the cell cycle process by samples from the Immunogenic subtype (p-value=7.8e-12) and the Notch by samples from the ADEX (p-value=8.2e-8) and Pancreatic Progenitor (p-value=6.1e-8) subtypes. The p-values for the degree of overlap calculated by a Hypergeometric test.

We computed the enrichment p-value of each overlap using the hipergeometric text:

```
phyperPvalue <- function(group1Name, group2Name) {
        tgroups <- factor(tgroupsICGC)
        levels(tgroups) <- c("CellCycle","Notch","Hedgehog")
        a <- data.frame("BaileyClasses"=clinicalICGC$ICGCsubtypes,
                        "group"=tgroups[match(rownames(clinicalICGC),
                                              names(tgroupsICGC))])
        a <- a[!is.na(a$BaileyClasses),]
        group1 <- sum(a$group == group1Name)
        group2 <- sum(a$BaileyClasses == group2Name)
        overlap <- sum(a$group == group1Name & a$BaileyClasses == group2Name)
        Totalnrgenes <- nrow(a)
        phyper (overlap, group2, Totalnrgenes-group2, group1, lower.tail=F)
}

sapply(c("CellCycle","Hedgehog","Notch"),
        function (x) {
                sapply(c("ADEX","Immunogenic","Squamous",
                         "PancreaticProgenitor"),
                function (y) {phyperPvalue(x,y)})})

##                           CellCycle     Hedgehog        Notch
```

```
## ADEX                    9.992600e-01 9.898259e-01 8.207257e-08
## Immunogenic             7.783549e-12 9.940054e-01 9.999967e-01
## Squamous                7.159243e-01 1.087957e-11 9.999936e-01
## PancreaticProgenitor 9.995554e-01 9.855011e-01 6.114662e-08
```

The hedgehog process is over-represented by samples from the Squamous subtype (p-value=1.1e-8), the cell cycle process by samples from the Immunogenic subtype (p-value=7.8e-12) and the Notch by samples from the ADEX (p-value=8.2e-8) and Pancreatic Progenitor (p-value=6.1e-8) subtypes.

12 Survival analysis

The R objects included with this data supplment, contain the tables with patient information for the TCGA and ICGC cohorts

```
survmatICGC <- mergewithGroups(survmatICGC, tgroupsICGC, groupLabels=c("CellCycle","Notch","Hedgehog"))
survmatTCGA <- mergewithGroups(survmatTCGA, tgroupsTCGA, groupLabels=c("Hedgehog","CellCycle","Notch"))

head(survmatICGC)

##          submitted_specimen_id Gender Age Overall.Survival.Months Overall.Survival.Status
## 8013861               8013861   male  59                   17.17                       1
## 8013218               8013218 female  77                   93.68                       1
## 8013274               8013274 female  58                   58.49                       0
## 8013915               8013915 female  78                   61.64                       1
## 8014201               8014201   male  63                   21.09                       1
## 8067814               8067814 female  49                   56.41                       0
##          Tumour.Stage    groups
## 8013861        T3N0MX     Notch
## 8013218     [Unknown] CellCycle
## 8013274     [Unknown]     Notch
## 8013915        T2N0MX CellCycle
## 8014201     [Unknown]  Hedgehog
## 8067814     [Unknown] CellCycle

head(survmatTCGA)

##                                                           bcr_aliquot_uuid Gender Age
## 435de9a0-3c2b-4b4f-87d1-92f37a0765b5 435de9a0-3c2b-4b4f-87d1-92f37a0765b5   male  48
## a737c8ad-5796-47f9-8505-4e7fb2a297ed a737c8ad-5796-47f9-8505-4e7fb2a297ed   male  75
## b9173a1f-a255-4e0e-88d2-ded795ba0e7b b9173a1f-a255-4e0e-88d2-ded795ba0e7b   male  71
## 23da230e-1c63-4e5b-bf92-8a1bec33073c 23da230e-1c63-4e5b-bf92-8a1bec33073c female  70
## 686f7d99-e94c-41a1-a22d-7fa1622828cb 686f7d99-e94c-41a1-a22d-7fa1622828cb   male  55
## 69c20a0f-f982-4974-b4ba-88b1cb9d72e5 69c20a0f-f982-4974-b4ba-88b1cb9d72e5   male  73
##                                      Overall.Survival.Months Overall.Survival.Status
## 435de9a0-3c2b-4b4f-87d1-92f37a0765b5                   23.95                       0
## a737c8ad-5796-47f9-8505-4e7fb2a297ed                    9.63                       1
## b9173a1f-a255-4e0e-88d2-ded795ba0e7b                    2.63                       0
## 23da230e-1c63-4e5b-bf92-8a1bec33073c                   20.60                       1
## 686f7d99-e94c-41a1-a22d-7fa1622828cb                   19.94                       1
## 69c20a0f-f982-4974-b4ba-88b1cb9d72e5                   22.21                       0
##                                      Tumour.Stage Adjuvant.Postoperative.Targeted.Therapy
## 435de9a0-3c2b-4b4f-87d1-92f37a0765b5    Stage IIB                                     YES
## a737c8ad-5796-47f9-8505-4e7fb2a297ed    Stage IIA                                     YES
## b9173a1f-a255-4e0e-88d2-ded795ba0e7b    Stage IIB                                     YES
```

```
## 23da230e-1c63-4e5b-bf92-8a1bec33073c    Stage IIB                                     YES
## 686f7d99-e94c-41a1-a22d-7fa1622828cb    Stage IIB                                     YES
## 69c20a0f-f982-4974-b4ba-88b1cb9d72e5    Stage IIA                                     YES
##                                       Adjuvant.Postoperative.radiotherapy    groups
## 435de9a0-3c2b-4b4f-87d1-92f37a0765b5                                 NO CellCycle
## a737c8ad-5796-47f9-8505-4e7fb2a297ed                                 NO CellCycle
## b9173a1f-a255-4e0e-88d2-ded795ba0e7b                          [Unknown]  Hedgehog
## 23da230e-1c63-4e5b-bf92-8a1bec33073c                                YES     Notch
## 686f7d99-e94c-41a1-a22d-7fa1622828cb                                 NO  Hedgehog
## 69c20a0f-f982-4974-b4ba-88b1cb9d72e5                                YES  Hedgehog
```

We eliminated from the survival analysis 2 PDAC samples from the ICGC cohort and 1 sample from the TCGA cohort for which there was no available survival time data:

```
#ICGC: eliminated 2 samples
rownames(clinicalICGC)[is.na(clinicalICGC$donor_survival_time)]

## [1] "8044572" "8070206"

#TCGA: eliminated 1 sample
rownames(clinicalTCGA)[is.na(clinicalTCGA$Overall.Survival..Months.)]

## [1] "bf6773cf-f233-4990-885e-56073c92cb0c"
```

Summary statistics for Overall Survival (OS) are:

```
summary(survmatICGC$Overall.Survival.Months)

##    Min. 1st Qu.  Median    Mean 3rd Qu.    Max.
##    0.03    8.95   15.26   18.37   24.56   93.68

summary(survmatTCGA$Overall.Survival.Months)

##    Min. 1st Qu.  Median    Mean 3rd Qu.    Max.
##    0.00    9.13   15.31   18.75   22.21   90.05
```

We plot OS for all cases.

```
ICGC.os.fit <- survfit(Surv(survmatICGC$Overall.Survival.Months, survmatICGC$Overall.Survival.Status) ~ 1)
ICGC.os.fit

## Call: survfit(formula = Surv(survmatICGC$Overall.Survival.Months, survmatICGC$Overall.Survival.Status)
##     1)
##
##       n  events  median 0.95LCL 0.95UCL
##   240.0   146.0    17.7    16.0    22.9

TCGA.os.fit <- survfit(Surv(survmatTCGA$Overall.Survival.Months, survmatTCGA$Overall.Survival.Status) ~ 1)
TCGA.os.fit

## Call: survfit(formula = Surv(survmatTCGA$Overall.Survival.Months, survmatTCGA$Overall.Survival.Status)
##     1)
##
##       n  events  median 0.95LCL 0.95UCL
##   177.0    92.0    20.2    17.5    24.1

par(mfrow=c(1,2))
plot(ICGC.os.fit, main="Kaplan-Meier estimate for ICGC\nwith 95% confidence bounds",
xlab="time [months]", ylab="survival", cex.main=0.8)
plot(TCGA.os.fit, main="Kaplan-Meier estimate for TCGA\nwith 95% confidence bounds",
xlab="time [months]", ylab="survival", cex.main=0.8)
```

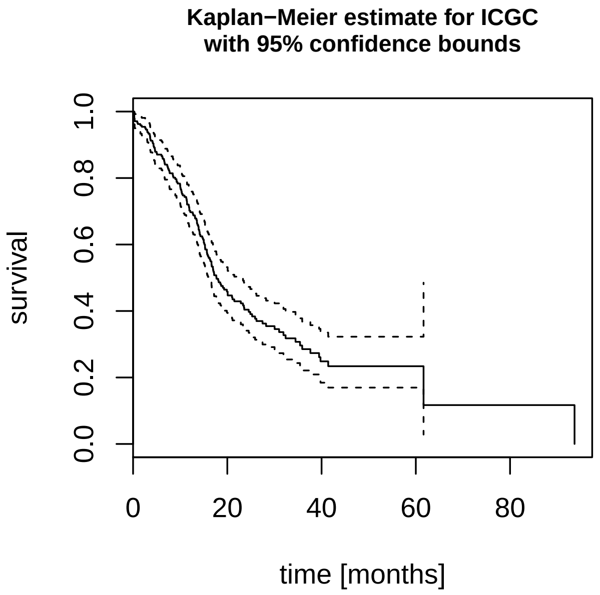

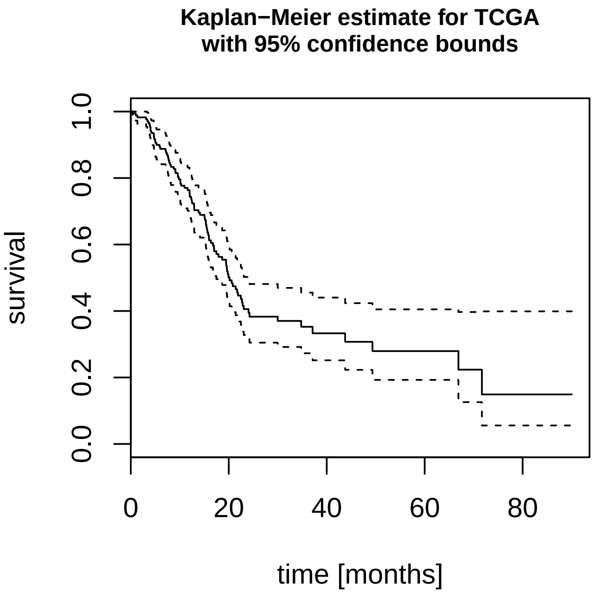

12.1 Survival differences in the ICGC cohort

We investigated survival differences between the three subgroups of tumours in the ICGC cohort.

```
survmat <- survmatICGC
table(survmat$groups)

##
## CellCycle  Hedgehog     Notch
##        90        37       113
```

The median Overall.Survival.Months differed between the three tumour subgroups.

```
library(rms)
ss <- Surv(survmat$Overall.Survival.Months,survmat$Overall.Survival.Status)
fit <- npsurv(ss ~ groups, data=survmat)
fit

## Call: npsurv(formula = ss ~ groups, data = survmat)
##
##                     n events median 0.95LCL 0.95UCL
## groups=CellCycle  90     57   17.0    12.1    31.9
## groups=Hedgehog   37     32   13.1    10.4    16.3
## groups=Notch     113     57   21.5    17.7    39.8
```

We test the significance of this survival difference using the log-rank test.

```
res1 <- survdiff(ss ~ groups, data=survmat)
p1 <- 1-pchisq(res1$chisq,2)
res1

## Call:
## survdiff(formula = ss ~ groups, data = survmat)
##
##                     N Observed Expected (O-E)^2/E (O-E)^2/V
```

```
## groups=CellCycle  90        57      52.5      0.382      0.616
## groups=Hedgehog   37        32      17.5     11.977     13.763
## groups=Notch     113        57      76.0      4.733     10.081
##
##  Chisq= 17.2  on 2 degrees of freedom, p= 0.00018
```

```
p1
```

```
## [1] 0.0001803876
```

```
ntab = table(survmat$groups)
txtlow = sprintf("%s (n=%d)", names(ntab)[1], ntab[1])
txtmed = sprintf("%s (n=%d)", names(ntab)[2], ntab[2])
txthigh = sprintf("%s (n=%d)", names(ntab)[3], ntab[3])

colours <- c("deeppink", "royalblue", "mediumseagreen")
survplot(fit, conf="none", n.risk=TRUE, col=colours,
         xlab="Overall survival [months]", lwd=3,las=1, lty=1)
legend("topright", c(txtlow, txtmed, txthigh), col=colours, lwd=2)
```

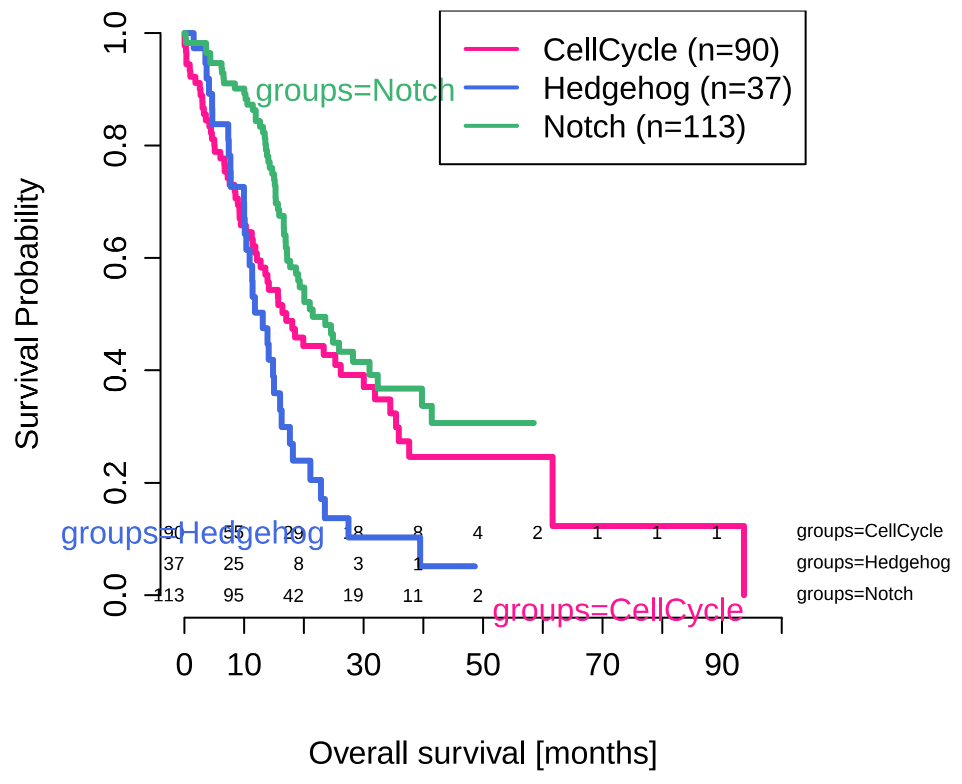

Figure 20: KaplanMeier survival curves of the different tumor subgroups using the ICGC cohort. Numbers of subjects at risk at the start of each time interval are shown above the x-axis.

Here we use multivariate Cox-hazard regression to assess if this result holds up when corrected for classical prognostic

factors: gender, age and tumour stage at diagnosis.

In the following analysis we use the anova() function to produce a sequential analysis of deviance table for the fitted model. Variables are added sequentially to the model in the order Gender, Age, Tumour.Stage, and finally groups. The p-value shows how significantly each variable contributes given the previously added variables.

```
## Cox-hazard model
model <- coxph(ss ~ Gender + Age + Tumour.Stage + groups, data=survmat)
summary(model)
```

```
## Call:
## coxph(formula = ss ~ Gender + Age + Tumour.Stage + groups, data = survmat)
##
##   n= 239, number of events= 145
##    (1 observation deleted due to missingness)
##
##                             coef exp(coef)  se(coef)      z Pr(>|z|)
## Gendermale              0.291666  1.338656  0.178435  1.635  0.10214
## Age                     0.023198  1.023469  0.009542  2.431  0.01505 *
## Tumour.StageIB         -2.681585  0.068455  1.292429 -2.075  0.03800 *
## Tumour.StageT2N0MX     -3.514787  0.029754  1.317085 -2.669  0.00762 **
## Tumour.StageT2NXMX     -3.513428  0.029795  1.502958 -2.338  0.01940 *
## Tumour.StageT3N0MX     -1.627040  0.196510  1.205818 -1.349  0.17723
## Tumour.StageT3N1MX     -0.960618  0.382656  1.464187 -0.656  0.51178
## Tumour.Stage[Unknown] -3.364809  0.034569  1.072018 -3.139  0.00170 **
## groupsHedgehog          0.548515  1.730682  0.231427  2.370  0.01778 *
## groupsNotch            -0.465401  0.627883  0.199257 -2.336  0.01951 *
## ---
## Signif. codes:  0 '***' 0.001 '**' 0.01 '*' 0.05 '.' 0.1 ' ' 1
##
##                        exp(coef) exp(-coef) lower .95 upper .95
## Gendermale               1.33866     0.7470  0.943593    1.8991
## Age                      1.02347     0.9771  1.004506    1.0428
## Tumour.StageIB           0.06845    14.6082  0.005436    0.8620
## Tumour.StageT2N0MX       0.02975    33.6088  0.002251    0.3932
## Tumour.StageT2NXMX       0.02979    33.5631  0.001566    0.5668
## Tumour.StageT3N0MX       0.19651     5.0888  0.018492    2.0882
## Tumour.StageT3N1MX       0.38266     2.6133  0.021702    6.7473
## Tumour.Stage[Unknown]    0.03457    28.9280  0.004228    0.2826
## groupsHedgehog           1.73068     0.5778  1.099579    2.7240
## groupsNotch              0.62788     1.5927  0.424885    0.9279
##
## Concordance= 0.652  (se = 0.027 )
## Rsquare= 0.141   (max possible= 0.997 )
## Likelihood ratio test= 36.19  on 10 df,   p=7.816e-05
## Wald test            = 43.67  on 10 df,   p=3.78e-06
## Score (logrank) test = 61.98  on 10 df,   p=1.525e-09
```

```
anova(model)
```

```
## Analysis of Deviance Table
##  Cox model: response is ss
## Terms added sequentially (first to last)
##
##               loglik   Chisq Df Pr(>|Chi|)
## NULL         -690.81
## Gender       -690.11  1.3976  1    0.23713
```

```
## Age          -687.89  4.4356  1    0.03520 *
## Tumour.Stage -682.09 11.6058  6    0.07136 .
## groups       -672.72 18.7484  2  8.489e-05 ***
## ---
## Signif. codes:  0 '***' 0.001 '**' 0.01 '*' 0.05 '.' 0.1 ' ' 1
```

In summary, stratifying patients by tumour subgroup of disease process is a significant predictor of OS, independent of covariates including gender, age and tumour stage.

12.2 Survival differences in the TCGA cohort

We investigated survival differences between the three subgroups of tumours in the TCGA cohort.

```
survmat <- survmatTCGA
table(survmat$groups)

##
## CellCycle  Hedgehog     Notch
##        73        45        59
```

The median Overall.Survival.Months differed between the three tumour subgroups.

```
fit <- npsurv(Surv(survmat$Overall.Survival.Months,
                   survmat$Overall.Survival.Status) ~ survmat$groups)
fit

## Call: npsurv(formula = Surv(survmat$Overall.Survival.Months, survmat$Overall.Survival.Status) ~
##     survmat$groups)
##
##                           n events median 0.95LCL 0.95UCL
## survmat$groups=CellCycle 73     44   19.5    15.9    37.1
## survmat$groups=Hedgehog  45     23   19.6    15.4    24.2
## survmat$groups=Notch     59     25   30.0    18.7      NA
```

First, we group the tumour stages into I - IV to increase the number of samples in each category, otherwise the first level ("Stage I") only contains 1 patient and the Cox proportional hazards regression model will not fit the data properly:

```
table(survmat$Tumour.Stage)

##
##   Stage I  Stage IA  Stage IB Stage IIA Stage IIB Stage III  Stage IV [Unknown]
##         1         5        15        28       118         4         4         2

survmat$Tumour.Stage <- as.character(survmat$Tumour.Stage)
tumdic <- c("Stage I"="I",  "Stage IA"="I", "Stage IB"="I", "Stage IIA"="IIA",
 "Stage IIB" = "IIB", "Stage III"="III" , "Stage IV"= "IV","[Unknown]"="[Unknown]")
survmat$Tumour.Stage <- tumdic[survmat$Tumour.Stage]
survmat$Tumour.Stage <- factor(survmat$Tumour.Stage, levels=c("I","IIA","IIB","III","IV"))
table(survmat$Tumour.Stage)

##
##   I IIA IIB III  IV
##  21  28 118   4   4
```

Using a multivariate Cox-hazard regression analysis we noticed a strong effect on the survival rates due to the different tretaments:

```
model <- coxph(Surv(survmat$Overall.Survival.Months,
                    survmat$Overall.Survival.Status) ~ Gender +
```

```
                 Age + Tumour.Stage +  Adjuvant.Postoperative.radiotherapy +
                 Adjuvant.Postoperative.Targeted.Therapy + groups, data= survmat)
anova(model)
```

```
## Analysis of Deviance Table
##  Cox model: response is Surv(survmat$Overall.Survival.Months, survmat$Overall.Survival.Status)
## Terms added sequentially (first to last)
##
##                                          loglik  Chisq Df Pr(>|Chi|)
## NULL                                    -404.99
## Gender                                  -404.69 0.6036  1    0.43719
## Age                                     -401.38 6.6054  1    0.01017 *
## Tumour.Stage                            -396.90 8.9725  4    0.06179 .
## Adjuvant.Postoperative.radiotherapy     -392.82 8.1607  2    0.01690 *
## Adjuvant.Postoperative.Targeted.Therapy -389.58 6.4861  2    0.03904 *
## groups                                  -388.60 1.9484  2    0.37749
## ---
## Signif. codes:  0 '***' 0.001 '**' 0.01 '*' 0.05 '.' 0.1 ' ' 1
```

We split the cohorts into "treated" and "non-treated" based on the Adjuvant.Postoperative.Targeted.Therapy labels. We can see that there is some effect on the treatment:

```
par(mfrow=c(1,2))
ss <- Surv(survmat$Overall.Survival.Months, survmat$Overall.Survival.Status)

ntab = table(survmat$groups, survmat$Adjuvant.Postoperative.Targeted.Therapy)
txtlowYES = sprintf("%s (n=%d)", rownames(ntab)[1], ntab[1,"YES"])
txtmedYES = sprintf("%s (n=%d)", rownames(ntab)[2], ntab[2,"YES"])
txthighYES = sprintf("%s (n=%d)", rownames(ntab)[3], ntab[3,"YES"])
txtlowNO = sprintf("%s (n=%d)", rownames(ntab)[1], ntab[1,"NO"])
txtmedNO = sprintf("%s (n=%d)", rownames(ntab)[2], ntab[2,"NO"])
txthighNO = sprintf("%s (n=%d)", rownames(ntab)[3], ntab[3,"NO"])

colours <- c("deeppink", "royalblue", "mediumseagreen")

fit <- npsurv(ss ~ survmat$groups, subset= survmat$Adjuvant.Postoperative.Targeted.Therapy=="YES")
survplot(fit, conf="none", n.risk=TRUE, col=colours,
         xlab="Overall survival [months]", lwd=3,las=1, lty=1, main="treated")
legend("topright", c(txtlowYES, txtmedYES, txthighYES), col=colours, lwd=2, cex=0.5)

fit <- npsurv(ss ~ survmat$groups, subset=survmat$Adjuvant.Postoperative.Targeted.Therapy=="NO")
survplot(fit, conf="none", n.risk=TRUE, col=colours,
         xlab="Overall survival [months]", lwd=3,las=1, lty=1, main="non-treated")
legend("topright", c(txtlowNO, txtmedNO, txthighNO), col=colours, lwd=2, cex=0.5)
```

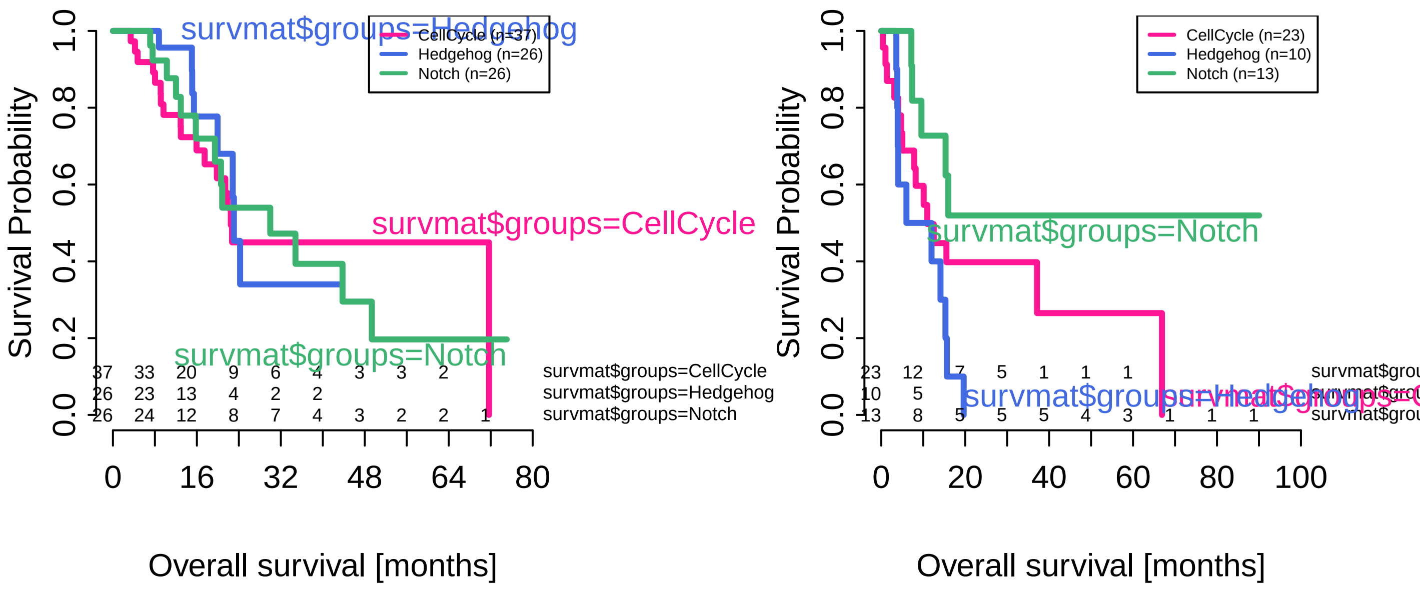

Figure 21: KaplanMeier survival curves of the different tumor subgroups using the TCGA cohort for subsets of individual that did or did not receive adjuvant targeted therapy treatment. Numbers of subjects at risk at the start of each time interval are shown above the x-axis.

We tested the interaction formally:

```
interacmodelTCGA <- coxph(ss ~ Gender + Age + Tumour.Stage + Adjuvant.Postoperative.radiotherapy +
groups*Adjuvant.Postoperative.Targeted.Therapy, data=survmat)
```

The obtained p-values are significant for the hedgehog group compared to the Cell Cycle group. The prognosis is worse only if the patientsin the Hedgehog group are not treated, which means that targeted treatment would be indicated for this group of patients.

```
anova(interacmodelTCGA)

## Analysis of Deviance Table
##  Cox model: response is ss
## Terms added sequentially (first to last)
##
##                                              loglik  Chisq Df Pr(>|Chi|)
## NULL                                        -404.99
## Gender                                      -404.69 0.6036  1    0.43719
## Age                                         -401.38 6.6054  1    0.01017 *
## Tumour.Stage                                -396.90 8.9725  4    0.06179 .
## Adjuvant.Postoperative.radiotherapy         -392.82 8.1607  2    0.01690 *
## groups                                      -391.70 2.2327  2    0.32747
## Adjuvant.Postoperative.Targeted.Therapy     -388.60 6.2018  2    0.04501 *
## groups:Adjuvant.Postoperative.Targeted.Therapy -384.31 8.5744  4    0.07266 .
## ---
```

```
## Signif. codes:  0 '***' 0.001 '**' 0.01 '*' 0.05 '.' 0.1 ' ' 1
```

```r
summary(interacmodelTCGA)
```

```
## Call:
## coxph(formula = ss ~ Gender + Age + Tumour.Stage + Adjuvant.Postoperative.radiotherapy +
##     groups * Adjuvant.Postoperative.Targeted.Therapy, data = survmat)
##
##   n= 175, number of events= 92
##    (2 observations deleted due to missingness)
##
##                                                               coef exp(coef)
## Gendermale                                                -0.30889   0.73426
## Age                                                        0.01529   1.01541
## Tumour.StageIIA                                            0.60702   1.83495
## Tumour.StageIIB                                            1.00719   2.73791
## Tumour.StageIII                                            0.44297   1.55733
## Tumour.StageIV                                             0.81291   2.25446
## Adjuvant.Postoperative.radiotherapyNO                      0.59775   1.81803
## Adjuvant.Postoperative.radiotherapy[Unknown]               0.12567   1.13391
## groupsHedgehog                                            -0.44713   0.63946
## groupsNotch                                               -0.09311   0.91109
## Adjuvant.Postoperative.Targeted.TherapyNO                  0.41274   1.51095
## Adjuvant.Postoperative.Targeted.Therapy[Unknown]           0.89922   2.45769
## groupsHedgehog:Adjuvant.Postoperative.Targeted.TherapyNO   1.41649   4.12261
## groupsNotch:Adjuvant.Postoperative.Targeted.TherapyNO     -0.36564   0.69375
## groupsHedgehog:Adjuvant.Postoperative.Targeted.Therapy[Unknown]  0.24527   1.27797
## groupsNotch:Adjuvant.Postoperative.Targeted.Therapy[Unknown]    -0.64297   0.52573
##                                                           se(coef)      z Pr(>|z|)
## Gendermale                                                 0.22238 -1.389   0.1648
## Age                                                        0.01117  1.368   0.1712
## Tumour.StageIIA                                            0.52380  1.159   0.2465
## Tumour.StageIIB                                            0.42483  2.371   0.0177
## Tumour.StageIII                                            1.12109  0.395   0.6928
## Tumour.StageIV                                             0.85920  0.946   0.3441
## Adjuvant.Postoperative.radiotherapyNO                      0.37369  1.600   0.1097
## Adjuvant.Postoperative.radiotherapy[Unknown]               0.86599  0.145   0.8846
## groupsHedgehog                                             0.43154 -1.036   0.3002
## groupsNotch                                                0.38273 -0.243   0.8078
## Adjuvant.Postoperative.Targeted.TherapyNO                  0.37605  1.098   0.2724
## Adjuvant.Postoperative.Targeted.Therapy[Unknown]           0.87146  1.032   0.3021
## groupsHedgehog:Adjuvant.Postoperative.Targeted.TherapyNO   0.61483  2.304   0.0212
## groupsNotch:Adjuvant.Postoperative.Targeted.TherapyNO      0.65144 -0.561   0.5746
## groupsHedgehog:Adjuvant.Postoperative.Targeted.Therapy[Unknown]  0.71101  0.345   0.7301
## groupsNotch:Adjuvant.Postoperative.Targeted.Therapy[Unknown]     0.62995 -1.021   0.3074
##
## Gendermale
## Age
## Tumour.StageIIA
## Tumour.StageIIB                                           *
## Tumour.StageIII
## Tumour.StageIV
## Adjuvant.Postoperative.radiotherapyNO
## Adjuvant.Postoperative.radiotherapy[Unknown]
## groupsHedgehog
```

```
## groupsNotch
## Adjuvant.Postoperative.Targeted.TherapyNO
## Adjuvant.Postoperative.Targeted.Therapy[Unknown]
## groupsHedgehog:Adjuvant.Postoperative.Targeted.TherapyNO        *
## groupsNotch:Adjuvant.Postoperative.Targeted.TherapyNO
## groupsHedgehog:Adjuvant.Postoperative.Targeted.Therapy[Unknown]
## groupsNotch:Adjuvant.Postoperative.Targeted.Therapy[Unknown]
## ---
## Signif. codes:  0 '***' 0.001 '**' 0.01 '*' 0.05 '.' 0.1 ' ' 1
##
##                                                                 exp(coef) exp(-coef)
## Gendermale                                                         0.7343     1.3619
## Age                                                                1.0154     0.9848
## Tumour.StageIIA                                                    1.8349     0.5450
## Tumour.StageIIB                                                    2.7379     0.3652
## Tumour.StageIII                                                    1.5573     0.6421
## Tumour.StageIV                                                     2.2545     0.4436
## Adjuvant.Postoperative.radiotherapyNO                              1.8180     0.5500
## Adjuvant.Postoperative.radiotherapy[Unknown]                       1.1339     0.8819
## groupsHedgehog                                                     0.6395     1.5638
## groupsNotch                                                        0.9111     1.0976
## Adjuvant.Postoperative.Targeted.TherapyNO                          1.5110     0.6618
## Adjuvant.Postoperative.Targeted.Therapy[Unknown]                   2.4577     0.4069
## groupsHedgehog:Adjuvant.Postoperative.Targeted.TherapyNO           4.1226     0.2426
## groupsNotch:Adjuvant.Postoperative.Targeted.TherapyNO              0.6938     1.4414
## groupsHedgehog:Adjuvant.Postoperative.Targeted.Therapy[Unknown]    1.2780     0.7825
## groupsNotch:Adjuvant.Postoperative.Targeted.Therapy[Unknown]       0.5257     1.9021
##                                                                 lower .95 upper .95
## Gendermale                                                         0.4749     1.135
## Age                                                                0.9934     1.038
## Tumour.StageIIA                                                    0.6573     5.122
## Tumour.StageIIB                                                    1.1907     6.296
## Tumour.StageIII                                                    0.1730    14.017
## Tumour.StageIV                                                     0.4185    12.145
## Adjuvant.Postoperative.radiotherapyNO                              0.8740     3.782
## Adjuvant.Postoperative.radiotherapy[Unknown]                       0.2077     6.190
## groupsHedgehog                                                     0.2745     1.490
## groupsNotch                                                        0.4303     1.929
## Adjuvant.Postoperative.Targeted.TherapyNO                          0.7230     3.158
## Adjuvant.Postoperative.Targeted.Therapy[Unknown]                   0.4454    13.562
## groupsHedgehog:Adjuvant.Postoperative.Targeted.TherapyNO           1.2355    13.757
## groupsNotch:Adjuvant.Postoperative.Targeted.TherapyNO              0.1935     2.487
## groupsHedgehog:Adjuvant.Postoperative.Targeted.Therapy[Unknown]    0.3172     5.149
## groupsNotch:Adjuvant.Postoperative.Targeted.Therapy[Unknown]       0.1529     1.807
##
## Concordance= 0.692  (se = 0.034 )
## Rsquare= 0.21   (max possible= 0.99 )
## Likelihood ratio test= 41.35  on 16 df,   p=0.0004927
## Wald test            = 43.12  on 16 df,   p=0.0002676
## Score (logrank) test = 49.44  on 16 df,   p=2.811e-05
```

Hazard ratios are different for the two situations

```
par(mfrow=c(1,2))
rm(groups)

attach(survmat)
d <- datadist(survmat)
options(datadist="d")
fit1 = cph(ss ~ Gender + Age + Tumour.Stage + groups, survmat, x=T, y=T,
          subset=Adjuvant.Postoperative.Targeted.Therapy=="YES")
plot(summary(fit1), col=c("orange", "gold", "blue"))

fit1 = cph(ss ~ Gender + Age + Tumour.Stage + groups, survmat, x=T, y=T,
          subset=Adjuvant.Postoperative.Targeted.Therapy=="NO")
plot(summary(fit1), col=c("orange", "gold", "blue"))
```

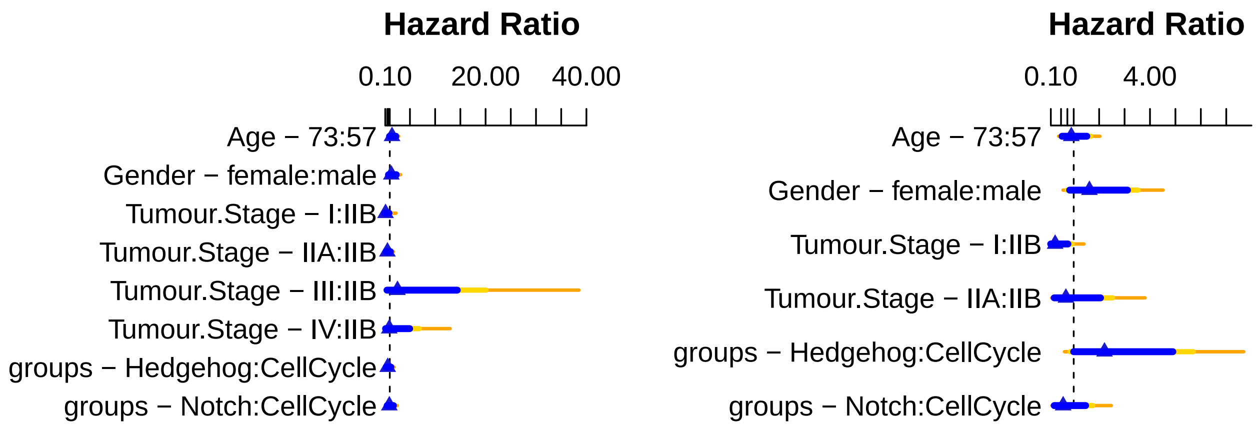


```
detach(survmat)
```

We can also examine the same effect of targeted treatment, by plotting the survival curves before and after target therapy separately for each group:

```
par(mfcol=c(1,3))
for (groupName in c("CellCycle","Hedgehog","Notch") ) {

  #log-rank model for targted-therapy
  d <- survmat[survmat$groups==groupName,]
  d <- d[d$Adjuvant.Postoperative.Targeted.Therapy %in% c("YES","NO"),]
  d$Adjuvant.Postoperative.Targeted.Therapy <- factor(d$Adjuvant.Postoperative.Targeted.Therapy, levels=c(
  attach(d)
  ss <- Surv(Overall.Survival.Months,Overall.Survival.Status)
  res1 <- survdiff(ss ~ Adjuvant.Postoperative.Targeted.Therapy, data=d)
  p2 <- 1-pchisq(res1$chisq,1) #1 degree of freendom

  #plot with survplot
  fit <- npsurv(ss ~ Adjuvant.Postoperative.Targeted.Therapy)
```

```
  colours <- c("darkorange2", "darkolivegreen")
  survplot(fit, conf="none", n.risk=T, col=colours, xlab="Overall survival [months]", lwd=3,las=1, lty=1)
  title(main=paste(groupName, "\nTarget.Therapy"))

  #write p-value
  if (groupName!="Hedgehog") {p2 <- round(p2,3)}else{p2 <- round(p2, 9)}
  if (groupName=="Notch") {text(15, 0.2, paste0("p=", p2))}
  if (groupName == "CellCycle"){text(10, 0.2, paste0("p=", p2))}
  if  (groupName == "Hedgehog"){text(30, 0.2, paste0("p=", p2))}
  detach(d)

  #legend
  ntab = table(d$Adjuvant.Postoperative.Targeted.Therapy)
  txtlow = sprintf("treated (n=%d)", ntab[1])
  txthigh = sprintf("non-treated (n=%d)",ntab[2])
  legend("topright", c(txtlow, txthigh), col=colours, lwd=2)
}
```

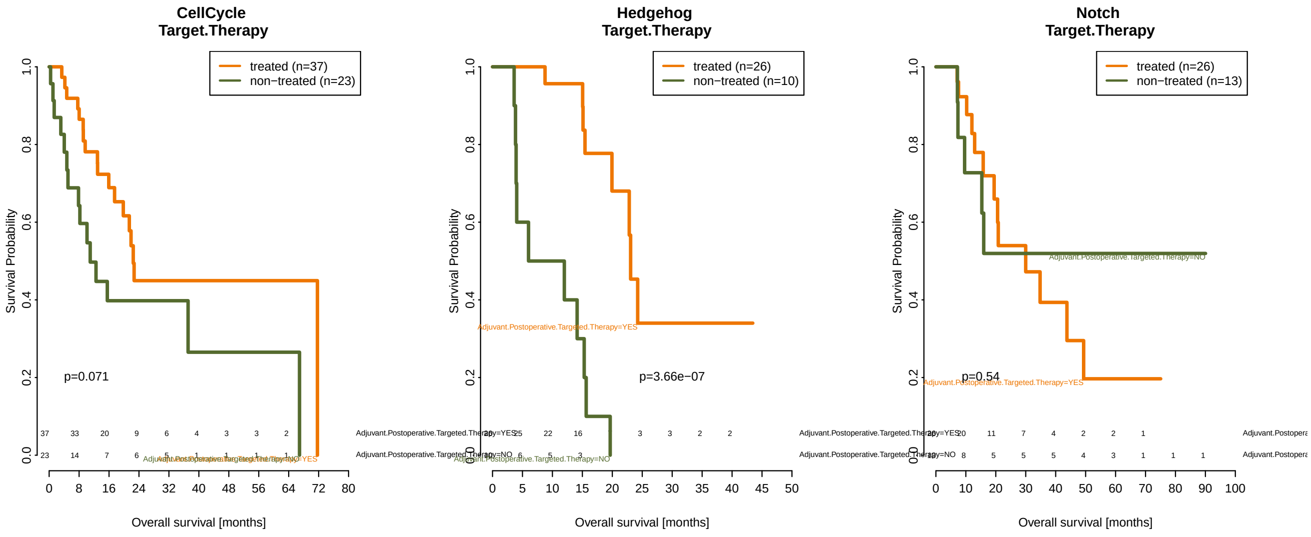

13 Analysis of mutation data

We next investigated the molecular basis for these survival differences and compared the mutational patterns between the three subtypes

For the ICGC cohort, we used the previously identified landscape (Bailey et al., 2016) of somatic mutations and copy number alterations in the ICGC cohort to investigate the frequency of samples with mutations in key 39 genes in pancreatic cancer (Waddell et al., 2015). Somatic mutations were previously filtered out to include only alterations with high and moderate impact on the basis of their predicted effect on the protein (Cingolani et al., 2012). Copy number alterations were restricted to losses of at least 1 copy and to amplifications of more than 5 copies.

The TCGA mutation data was downloaded directly from the cBioPortal. The copy-number data was downloaded in the form of GISTIC scores (Gscoes >= 1 represents a gain and Gscore <= -1 represents a loss).

```
load("data/PreComputedTCGAfreqmutadata.RData")
getfreq <- function(x) {
        sapply(split(x, x$groups), function(xx) length(unique(xx$submitted_specimen_id)))
```

```
}

#Number of samples in each group
tgroups <- factor(tgroupsICGC)
levels(tgroups) <- c("CellCycle","Notch","Hedgehog")
N <- unlist(c(table(tgroups)))[c("CellCycle","Hedgehog","Notch")]
N

## CellCycle  Hedgehog     Notch
##        90        37       115

#frequency of altered samples per gene
a <- split(gain, gain$hgnc_symbol)
a <- t(sapply(a, getfreq))[,c("CellCycle","Hedgehog","Notch")]
gain.freq <- a*100 / N

a <- split(loss, loss$hgnc_symbol)
a <- t(sapply(a,getfreq))[,c("CellCycle","Hedgehog","Notch")]
loss.freq <- a*100 / N

a <- split(mutadata, mutadata$hgnc_symbol)
a <- t(sapply(a, getfreq))[,c("CellCycle","Hedgehog","Notch")]
mutadata.freq <- a*100 / N


#Plot
library(reshape2)
library(ggplot2)

a <- data.frame(rbind(cbind("type"="gain",melt(gain.freq)),
                                          cbind("type"="loss",melt(loss.freq)),
                                          cbind("type"="mut", melt(mutadata.freq))))
colnames(a) <- c("type","symbol","group","freq")

#Plot
ggplot(a, aes(factor(symbol, levels=PDACgenes), freq, fill=group)) +
  geom_bar(stat="identity", position="dodge") +
  facet_grid(type ~ ., scales="free") +
  xlab("Key genes and pathways in pancreatic cancer") +
  scale_fill_manual(values=c("deeppink","royalblue","mediumseagreen")) +
  theme(axis.text.x=element_text(angle=90,hjust=1,vjust=0.5)) +
  ylab("Frequency of altered samples") + ggtitle("ICGC cohort")
ggplot(tcga_mutFreq, aes(factor(symbol, levels=PDACgenes), freq, fill=group)) +
  geom_bar(stat="identity", position="dodge") +
  facet_grid(type ~ ., scales="free") +
  xlab("Key genes and pathways in pancreatic cancer") +
  scale_fill_manual(values=c("deeppink","royalblue","mediumseagreen")) +
  theme(axis.text.x=element_text(angle=90,hjust=1,vjust=0.5)) +
  ylab("Frequency of altered samples") + ggtitle("TCGA cohort")
```

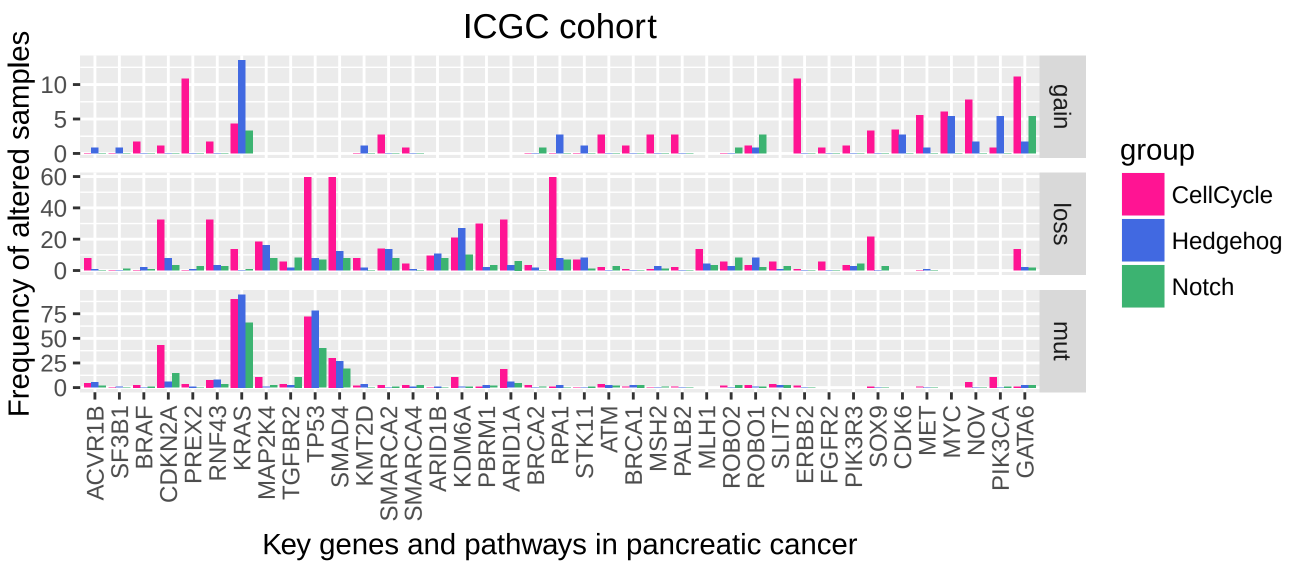

Figure 22: Mutations in key genes and pathways in pancreatic cancer. The upper and middle panels show the frequency of altered samples by gains or losses in copy number (gains refer to amplifications of >5 copies), bottom panel shows the frequency of altered samples by non-silent single nucleotide variants, small insertions or deletions with moderate-to-high biological effect.

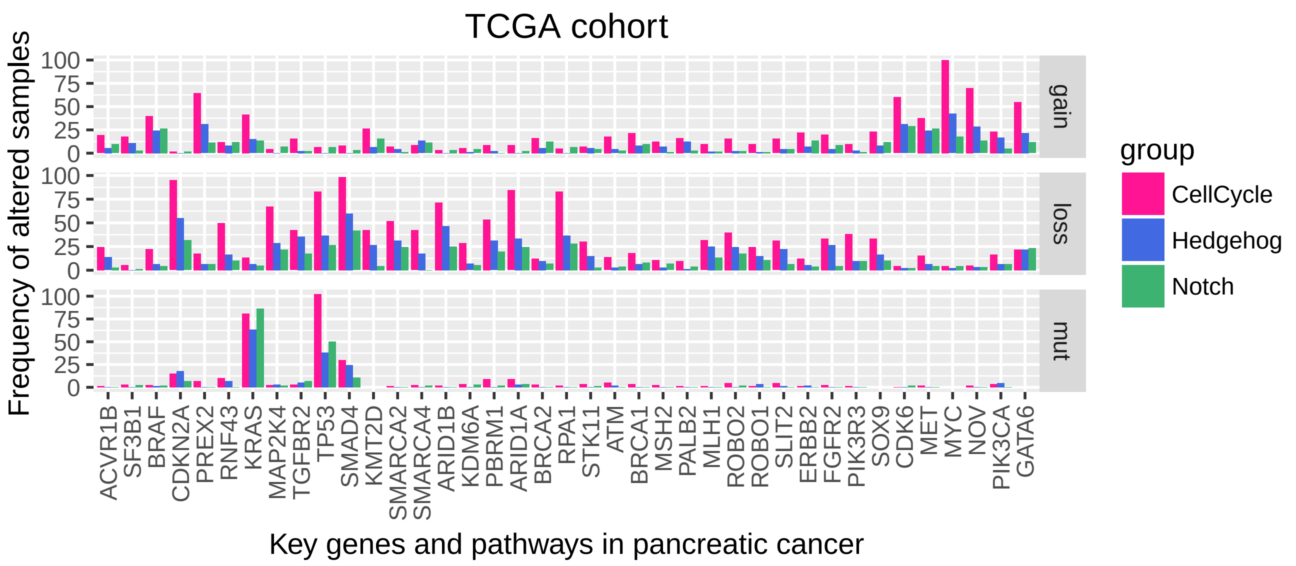

Figure 23: Mutations in key genes and pathways in the TCGA cohort. The upper and middle panels show the frequency of altered samples by copy number changes (GISTIC score >= 1 for gains, GISTIC score <= -1 for losses) bottom panel shows the frequency of altered samples by non-synonymous somatic mutations in gene coding regions.

We found that the Cell Cycle process had a higher mutational burden than the Hedgehog/Wnt and Notch processes, with an average of 9.4% of altered samples in the Cell Cycle group, compared to 4.6% and 3.2% in the Hedgehog/Wnt and Notch groups, respectively

```
print(aggregate(a$freq, by=list(a$group), FUN=mean))

##     Group.1        x
## 1 CellCycle 9.436477
## 2  Hedgehog 4.560354
## 3     Notch 3.218929
```

The following table shows in more detailed the data orignitaing the plot:

```
kable(gain.freq)
```

	CellCycle	Hedgehog	Notch
ACVR1B	0.0000000	0.8695652	0.0000000
ATM	2.7027027	0.0000000	0.0000000
BRAF	1.7391304	0.0000000	0.0000000
BRCA1	1.1111111	0.0000000	0.0000000
BRCA2	0.0000000	0.0000000	0.8695652
CDK6	3.4782609	2.7027027	0.0000000
CDKN2A	1.1111111	0.0000000	0.0000000
ERBB2	10.8108108	0.0000000	0.0000000
FGFR2	0.8695652	0.0000000	0.0000000
GATA6	11.1111111	1.7391304	5.4054054
KMT2D	0.0000000	1.1111111	0.0000000
KRAS	4.3478261	13.5135135	3.3333333
MET	5.5555556	0.8695652	0.0000000
MSH2	2.7027027	0.0000000	0.0000000
MYC	6.0869565	5.4054054	0.0000000
NOV	7.7777778	1.7391304	0.0000000
PALB2	2.7027027	0.0000000	0.0000000
PIK3CA	0.8695652	5.4054054	0.0000000
PIK3R3	1.1111111	0.0000000	0.0000000
PREX2	10.8108108	0.0000000	0.0000000
RNF43	1.7391304	0.0000000	0.0000000
ROBO1	1.1111111	0.8695652	2.7027027
ROBO2	0.0000000	0.0000000	0.8695652
RPA1	0.0000000	2.7027027	0.0000000
SF3B1	0.0000000	0.8695652	0.0000000
SMARCA2	2.7027027	0.0000000	0.0000000
SMARCA4	0.8695652	0.0000000	0.0000000
SOX9	3.3333333	0.0000000	0.0000000
STK11	0.0000000	1.1111111	0.0000000

```
kable(loss.freq)
```

	CellCycle	Hedgehog	Notch
ACVR1B	7.7777778	0.8695652	0.0000000
ARID1A	32.4324324	3.3333333	6.0869565
ARID1B	9.5652174	10.8108108	7.7777778
ATM	2.2222222	0.0000000	2.7027027
BRAF	0.0000000	2.2222222	0.8695652
BRCA1	0.8695652	0.0000000	0.0000000
BRCA2	3.3333333	1.7391304	0.0000000
CDKN2A	32.4324324	7.7777778	3.4782609
ERBB2	0.8695652	0.0000000	0.0000000
FGFR2	5.5555556	0.0000000	0.0000000
GATA6	13.5135135	2.2222222	1.7391304
KDM6A	20.8695652	27.0270270	10.0000000
KMT2D	7.7777778	1.7391304	0.0000000
KRAS	13.5135135	0.0000000	0.8695652
MAP2K4	18.2608696	16.2162162	7.7777778
MET	0.0000000	0.8695652	0.0000000
MLH1	13.5135135	4.4444444	3.4782609
MSH2	0.8695652	2.7027027	1.1111111
PALB2	2.2222222	0.0000000	0.0000000
PBRM1	29.7297297	2.2222222	3.4782609
PIK3R3	3.4782609	2.7027027	4.4444444
PREX2	0.0000000	0.8695652	2.7027027
RNF43	32.4324324	3.3333333	2.6086957
ROBO1	3.4782609	8.1081081	2.2222222
ROBO2	5.5555556	2.6086957	8.1081081
RPA1	59.4594595	7.7777778	6.9565217
SF3B1	0.0000000	0.0000000	1.1111111
SLIT2	5.5555556	0.8695652	2.7027027
SMAD4	59.4594595	12.2222222	7.8260870
SMARCA2	13.9130435	13.5135135	7.7777778
SMARCA4	4.4444444	0.8695652	0.0000000
SOX9	21.6216216	0.0000000	2.6086957
STK11	6.9565217	8.1081081	1.1111111
TGFBR2	5.5555556	1.7391304	8.1081081
TP53	59.4594595	7.7777778	6.9565217

```
kable(mutadata.freq)
```

	CellCycle	Hedgehog	Notch
ACVR1B	4.4444444	5.4054054	1.7391304
ARID1A	18.9189189	6.0869565	4.4444444
ARID1B	0.0000000	1.1111111	0.0000000
ATM	3.3333333	2.7027027	1.7391304
BRAF	2.7027027	0.0000000	1.1111111
BRCA1	0.8695652	2.2222222	2.7027027
BRCA2	2.2222222	0.0000000	0.8695652
CDKN2A	43.2432432	6.0869565	14.4444444
ERBB2	1.7391304	0.0000000	0.0000000
GATA6	1.1111111	2.7027027	2.6086957
KDM6A	10.8108108	0.8695652	1.1111111
KMT2D	1.7391304	3.3333333	0.0000000
KRAS	90.0000000	94.5945946	66.0869565
MAP2K4	10.8108108	0.8695652	2.2222222
MET	0.8695652	0.0000000	0.0000000
MSH2	0.0000000	0.0000000	0.8695652
NOV	5.4054054	0.0000000	0.0000000
PALB2	0.8695652	0.0000000	0.0000000
PBRM1	1.1111111	2.7027027	1.7391304
PIK3CA	10.8108108	0.0000000	1.1111111
PREX2	3.4782609	1.1111111	0.0000000
RNF43	7.7777778	8.1081081	3.4782609
ROBO1	2.7027027	0.8695652	1.1111111
ROBO2	1.7391304	0.0000000	2.7027027
RPA1	1.1111111	2.7027027	0.0000000
SF3B1	0.0000000	0.8695652	0.0000000
SLIT2	3.4782609	2.2222222	2.7027027
SMAD4	30.0000000	27.0270270	19.1304348
SMARCA2	2.7027027	0.0000000	1.1111111
SMARCA4	2.6086957	1.1111111	2.7027027
SOX9	1.1111111	0.0000000	0.0000000
STK11	0.0000000	0.0000000	1.1111111
TGFBR2	3.4782609	2.2222222	10.8108108
TP53	72.2222222	78.3783784	40.0000000

14 References

Alvarez, M. J. et al. Inferring global protein activity profiles by network based analysis of gene expression signatures. Manuscript in preparation.

Anders, S. Huber, W. Differential expression analysis for sequence count data. Genome Biol 11, R106, doi:10.1186/gb-2010-11-10-r106 (2010).

Badea, L., Herlea, V., Dima, S. O., Dumitrascu, T. Popescu, I. Combined gene expression analysis of whole-tissue and microdissected pancreatic ductal adenocarcinoma identifies genes specifically overexpressed in tumor epithelia. Hepato-gastroenterology 55, 2016-2027 (2008).

Bailey, P. et al. Genomic analyses identify molecular subtypes of pancreatic cancer. Nature 531, 47-52, doi:10.1038/nature16965 (2016).

Carro, M. S. et al. The transcriptional network for mesenchymal transformation of brain tumours. Nature 463, 318-25 (2010).

Castro, M. A. et al. Regulators of genetic risk of breast cancer identified by integrative network analysis. Nat Genet 48, 12-21, doi:10.1038/ng.3458 (2016).

Cingolani, P. et al. A program for annotating and predicting the effects of single nucleotide polymorphisms, SnpEff: SNPs in the genome of Drosophila melanogaster strain w1118; iso-2; iso-3. Fly (Austin) 6, 80-92, doi:10.4161/fly.19695 (2012).

Clauset, A., Newman, M. E. Moore, C. Finding community structure in very large networks. Phys Rev E Stat Nonlin Soft Matter Phys 70, 066111, doi:10.1103/PhysRevE.70.066111 (2004).

Collisson, E. A. et al. Subtypes of pancreatic ductal adenocarcinoma and their differing responses to therapy. Nat Med 17, 500-503, doi:10.1038/nm.2344 (2011). 9 Bailey, P. et al. Genomic analyses identify molecular subtypes of pancreatic cancer. Nature 531, 47-52, doi:10.1038/nature16965 (2016).

Donahue, T. R. et al. Integrative survival-based molecular profiling of human pancreatic cancer. Clin Cancer Res 18, 1352-1363, doi:10.1158/1078-0432.CCR-11-1539 (2012).

Edgar R, Domrachev M, Lash AE. Gene Expression Omnibus: NCBI gene expression and hybridization array data repository. Nucleic Acids Res. 2002;30(1):20710.

Expression Project for Oncology (expO) seeks to integrate longitudinal clinical annotation with gene expression data for a unique and powerful portrait of human malignancies, providing critical perspective on diagnostic markers, prognostic indicators, and therapeutic targets., ¡ https://expo.intgen.org/geo/home.do¿ (2015).

Fletcher, M. N. et al. Master regulators of FGFR2 signalling and breast cancer risk. Nat Commun 4, 2464, doi:10.1038/ncomms3464 (2013).

Gautier, L., Cope, L., Bolstad, B. M. Irizarry, R. A. affy–analysis of Affymetrix GeneChip data at the probe level. Bioinformatics 20, 307-315, doi:10.1093/bioinformatics/btg405 (2004).

Irizarry RA, Hobbs B, Collin F, Beazer-Barclay YD, Antonellis KJ, Scherf U, Speed TP. Exploration, normalization, and summaries of high density oligonucleotide array probe level data. Biostatistics (2003).

Johnson WE, Li C, Rabinovic A. Adjusting batch effects in microarray data using empirical bayes methods. Biostatistics, 8(1):118127, 2007

Opgen-Rhein, R. K. Strimmer, K. Accurate ranking of differentially expressed genes by a distribution-free shrinkage approach. Statist. Appl. Genet. Mol. Biol. 6:9 (2007).

Pei, H. et al. FKBP51 affects cancer cell response to chemotherapy by negatively regulating Akt. Cancer Cell 16, 259-266, doi:10.1016/j.ccr.2009.07.016 (2009).

Ritchie, M. E. et al. limma powers differential expression analyses for RNA-sequencing and microarray studies. Nucleic Acids Res 43, e47, doi:10.1093/nar/gkv007 (2015).

Schafer, J. Strimmer, K. A shrinkage approach to large-scale covariance matrix estimation and implications for functional genomics. Stat Appl Genet Mol Biol 4, Article32, doi:10.2202/1544-6115.1175 (2005).

Stouffer, S. A., Edward A. Suchman, Leland C. DeVinney, Shirley A. Star, and Robin M. Williams, Jr. Studies in Social Psychology in World War II: The American Soldier. Vol. 1, Adjustment During Army Life., (Princeton University Press., 1949).

Strimmer, K. fdrtool: a versatile R package for estimating local and tail area-based false discovery rates. Bioinformatics 24, 1461-1462, doi:10.1093/bioinformatics/btn209 (2008).

The Cancer Genome Atlas Data Portal, ¡http://cancergenome.nih.gov¿ (2015).

Waddell, N. et al. Whole genomes redefine the mutational landscape of pancreatic cancer. Nature 518, 495-501, doi:10.1038/nature14169 (2015).

15 Session Info

```
sessionInfo()

## R version 3.3.1 (2016-06-21)
## Platform: x86_64-apple-darwin13.4.0 (64-bit)
## Running under: OS X 10.10.5 (Yosemite)
##
## locale:
## [1] en_GB.UTF-8/en_GB.UTF-8/en_GB.UTF-8/C/en_GB.UTF-8/en_GB.UTF-8
##
## attached base packages:
##  [1] grid      parallel  stats4    stats     graphics  grDevices utils     datasets
##  [9] methods   base
##
## other attached packages:
##  [1] ReactomePA_1.16.2                  clusterProfiler_3.0.5
##  [3] DOSE_2.10.7                        sigPathway_1.40.0
##  [5] rms_4.5-0                          SparseM_1.72
##  [7] Hmisc_3.17-4                       Formula_1.2-1
##  [9] lattice_0.20-33                    survival_2.39-5
## [11] cluster_2.0.4                      snow_0.4-1
## [13] KEGG.db_3.2.3                      GO.db_3.3.0
## [15] org.Mm.eg.db_3.3.0                 HTSanalyzeR_2.24.0
## [17] RTN_1.11.2                         pheatmap_1.0.8
## [19] ggplot2_2.1.0                      RedeR_1.20.0
## [21] viper_1.7.4                        fdrtool_1.2.15
## [23] corpcor_1.6.8                      biomaRt_2.28.0
## [25] limma_3.28.21                      reshape2_1.4.1
## [27] igraph_1.0.1                       Rsubread_1.22.3
## [29] xtable_1.8-2                       devtools_1.12.0
## [31] made4_1.46.0                       scatterplot3d_0.3-37
## [33] gplots_3.0.1                       RColorBrewer_1.1-2
## [35] ade4_1.7-4                         sva_3.20.0
## [37] genefilter_1.54.2                  mgcv_1.8-13
## [39] nlme_3.1-128                       affyPLM_1.48.0
## [41] preprocessCore_1.34.0              gcrma_2.44.0
## [43] affy_1.50.0                        hgu133plus2hsentrezgprobe_19.0.0
## [45] hgu133plus2hsentrezgcdf_19.0.0     hgu133plus2hsentrezg.db_19.0.0
## [47] org.Hs.eg.db_3.3.0                 AnnotationDbi_1.34.4
## [49] IRanges_2.6.1                      S4Vectors_0.10.2
## [51] Biobase_2.32.0                     BiocGenerics_0.18.0
## [53] knitr_1.14
##
## loaded via a namespace (and not attached):
##  [1] plyr_1.8.4            GSEABase_1.34.1      splines_3.3.1        TH.data_1.0-7
##  [5] digest_0.6.10         BiocInstaller_1.22.3 GOSemSim_1.30.3      gdata_2.17.0
##  [9] magrittr_1.5          memoise_1.0.0        mixtools_1.0.4       prada_1.48.0
## [13] Biostrings_2.40.2     annotate_1.50.0      pvclust_2.0-0        matrixStats_0.50.2
## [17] sandwich_2.3-4        colorspace_1.2-6     rappdirs_0.3.1       rrcov_1.4-3
## [21] RCurl_1.95-4.8        graph_1.50.0         lme4_1.1-12          zoo_1.7-13
## [25] gtable_0.2.0          zlibbioc_1.18.0      XVector_0.12.1       MatrixModels_0.4-1
## [29] RankProd_2.44.0       graphite_1.18.1      car_2.1-3            cellHTS2_2.36.0
## [33] DEoptimR_1.0-6        scales_0.4.0         vsn_3.40.0           mvtnorm_1.0-5
```

```
## [37] DBI_0.5-1            Rcpp_0.12.7         reactome.db_1.55.0  foreign_0.8-66
## [41] bit_1.1-12           acepack_1.3-3.3     ff_2.2-13           XML_3.98-1.4
## [45] nnet_7.3-12          labeling_0.3        munsell_0.4.3       tools_3.3.1
## [49] RSQLite_1.0.0        evaluate_0.9        stringr_1.0.0       robustbase_0.92-6
## [53] caTools_1.17.1       RBGL_1.48.1         quantreg_5.29       formatR_1.4
## [57] BioNet_1.32.0        DO.db_2.9           BiocStyle_2.0.3     pbkrtest_0.4-6
## [61] e1071_1.6-7          affyio_1.42.0       minet_3.30.0        tibble_1.2
## [65] pcaPP_1.9-61         stringi_1.1.1       highr_0.6           Matrix_1.2-6
## [69] nloptr_1.0.4         data.table_1.9.6    bitops_1.0-6        qvalue_2.4.2
## [73] latticeExtra_0.6-28  topGO_2.24.0        KernSmooth_2.23-15  gridExtra_2.2.1
## [77] codetools_0.2-14     polspline_1.1.12    boot_1.3-18         MASS_7.3-45
## [81] gtools_3.5.0         assertthat_0.1      chron_2.3-47        Category_2.38.0
## [85] withr_1.0.2          multcomp_1.4-6      rpart_4.1-10        tidyr_0.6.0
## [89] class_7.3-14         minqa_1.2.4         segmented_0.5-1.4
```
