# Supplementary material for: Master Regulators of Oncogenic KRAS Response in Pancreatic Cancer: An Integrative Network Biology Analysis
Source: PLoS Med. 2017 Jan 31;14(1):e1002223. doi: 10.1371/journal.pmed.1002223 (PMC5283690; doi:10.1371/journal.pmed.1002223)
Supplement: S2 Computational Analysis — Also included is a PDF output of the complied code. (ZIP) [file pmed.1002223.s012.zip › S2_Sweave/S2_Sweave.pdf]

Master regulators of KRAS response in pancreatic cancer
# – Supporting Information – Sweave 2 –

October 28, 2016

Contents

1 Function definitions 3

2 ESTIMATE Analysis 7

3 Immunological Pathways Analysis 14

4 CIBERSORT Analysis 19

5 Immune Checkpoint Analysis 25

6 Cancer Cell Line Analysis 29

Description

This document contains a set of documentation, R code, results and datasets to fully reproduce the analysis and results of the correspondent publication. This second half of the supporting documentation is split into four parts:

- Part I: ESTIMATE Analysis
- Part II: GSVA Analysis
- Part III: CIBERSORT Analysis
- Part IIII: Cell Line Analysis

Contents

In this section we probe the immune component of the microenvironment for the three subtypes of pancreatic cancer derived from transcription factor master regulator analysis. We first run the `"ESTIMATE"` package in order to obtain the inferred levels of stroma and immune activity present within the tumour sample. This method is based on single sample gene set enrichment analysis `"ssGSEA"` with gene signatures for stromal tissue and immune system modulators extracted from a set of tumour samples.

Furthermore, we leverage a novel deconvolution tool, `"CIBERSORT"` to gauge the fraction of immune phenotypes directly from the gene expression profile of a sample. This tool has proven to yield high efficacy in bulk tumour compositions of varying tumour cellularity. Patients with a higher immune infiltration will deconvolve more successfully and patients with a lower immune density in the tumour will deconvolve poorly or even fail to deconvolve at all. The mathematics governing the deconvolution algorithm are described in the CIBERSORT paper cited in the main paper.

Finally, we run `"ssGSEA"` with a curated set of immunological pathways in order to elucidate the mechanisms underlying the immune response within the different subtypes.

1 Function definitions

We first declare a function that will check if the data required for the analysis is within the working directory of the Sweave file and to download the required files otherwise.

```
#Load the package manager function
codePath <- paste(getwd(),"/code/",sep="")
dataPath <- paste(getwd(),"/data/",sep="")
site <- "www.lightcone.net/"
if (!file.exists(dataPath)) dir.create(dataPath)
if (!file.exists(codePath)) dir.create(codePath)

lightGet <- function(x,type)
{
  if(type=="code")
{
  codePath <- paste(codePath,x,sep="")
  if(file.exists(codePath)){print(paste(x,"is stored locally"));
    return(codePath)}
  else{download.file(paste(site,x,sep=""),codePath,"curl");
    return(codePath)}
}
  else
  {
  dataPath <- paste(dataPath,x,sep="")
  if(file.exists(dataPath)){print(paste(x,"is stored locally"));
    return(dataPath)}
  else{download.file(paste(site,x,sep=""),dataPath,"curl");
    return(dataPath)}
  }
}
```

We now define the functions that we utilise for the analyses throughout the sweave file.

```
#Function Definitions
#Load the package manager function
pkgTest <- function(x)
{
  if (!require(x,character.only = TRUE))
  {
    install.packages(x,dep=TRUE, repos="http://cran.rstudio.com/")
    if(!require(x,character.only = TRUE))
    {
      source("http://bioconductor.org/biocLite.R")
      biocLite(x)
      if(!require(x,character.only = TRUE)) stop("Package not found")
    }
  }
}
pkgTest("GSVA")
pkgTest("ppcor")
pkgTest("ggplot2")

## Warning:  package 'ggplot2' was built under R version 3.2.4

pkgTest("reshape2")
```

```
pkgTest("pamr")

#Define a function to test association between GSVA score and subtype
gTest <- function(moo,sets,path){
  set1 <- as.numeric(moo[moo[,78] == sets[1],path])
  set2 <- as.numeric(moo[moo[,78] == sets[2],path])
  wilcox.test(set1,set2)
}

#Define a function to perform the ESTIMATE analysis
runEstimate <- function(newGEP, name) {#Create gct file
  gctFn = "~/Desktop/gexpGSEA.gct"
  saveLoc <- "~/Desktop/estimateSave.txt"
  #Grab the estimate function
  codeDir = paste(getwd(),"/Code/",sep="")
  unzip(lightGet("estimate.zip","code"),exdir = codeDir)
  print(codeDir)
  library("estimate", lib.loc=codeDir)
  write.table(newGEP,saveLoc,quote = F,row.names=T,sep="\t",col.names=T)
  filterCommonGenes(input.f=saveLoc, output.f=gctFn, id="GeneSymbol")
  estimateScore(gctFn,"~/Desktop/output.gct",platform = "illumina")
  out <- data.frame(read.table("~/Desktop/output.gct",fill=T,sep="\t",
                             row.names=1, header=TRUE,stringsAsFactors = F))
  out <- data.frame(t(rbind(out,rep(name,cohort = dim(out)[2]))),
                    stringsAsFactors = F)
  out <- out[-1,]
}

#Define a function to perform the CIBERSORT analysis
runCibersort <- function(GEP, tag,sig){
  GEP <- GEP[order(rownames(GEP)),]
  source(lightGet("Cibersort.R","code"))
  ptm <- proc.time()
  resultsDiscovery <-CIBERSORT(sig,GEP,
                              1000,T,
                              "~/Desktop/CibersortDiscoveryOutput.csv")
  print(proc.time() - ptm)
  data.frame(resultsDiscovery, tag = rep(tag, dim(GEP)[2]))
}
```

Now we load in the data and examine the gene level distribution of the ICGC and TCGA cohorts

```
#Load in the pancreatic data
load(lightGet("PDAC.Rda","data"))

## [1] "PDAC.Rda is stored locally"

dgexp <- grimmondGexp
dgroups <- tgroups_Grimm
vgexp <- TCGAvst
vgroups <- tgroups_TCGA
vgroups[vgroups == 3] = "Notch"

#Lets examine how the data is distributed
barplot(sort(apply(dgexp,1,median),decreasing=T),col='yellow',
```

```
        main='Distribution of ICGC Cohort Expression Matrix')
```

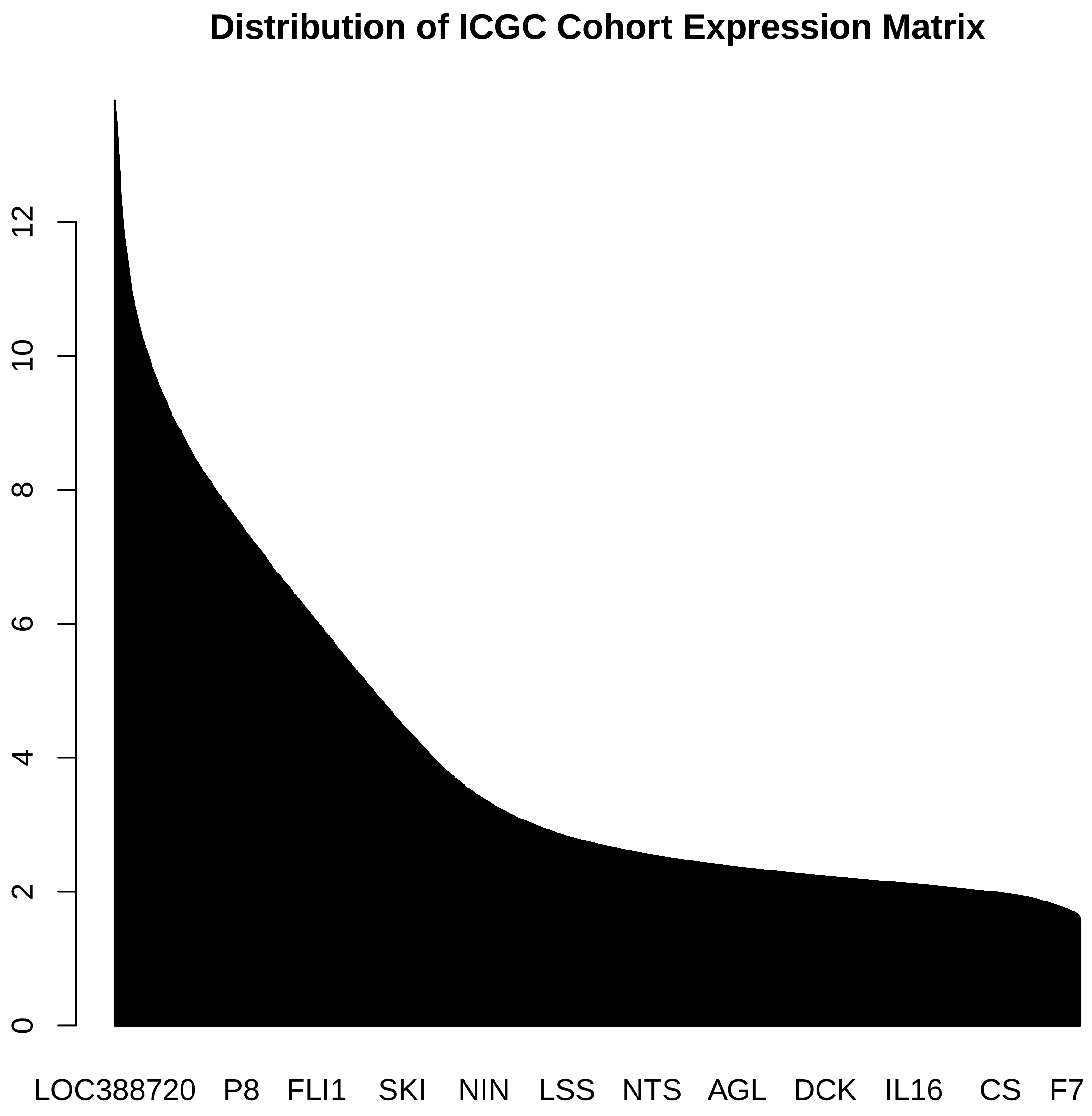


```
barplot(sort(apply(vgexp,1,median),decreasing=T),col='yellow',
        main='Distribution of TCGA Cohort Expression Matrix')
```

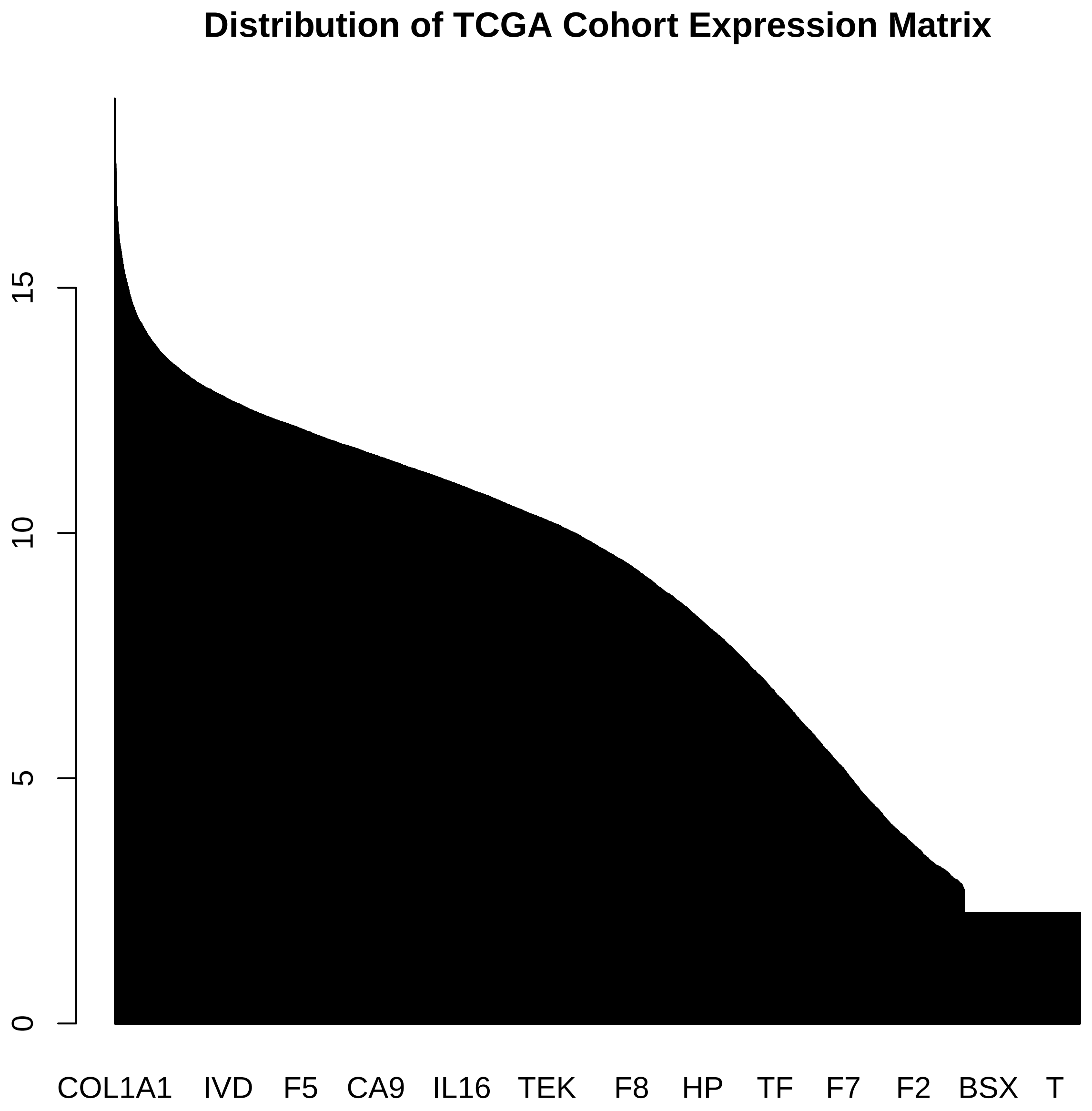


```
#Lets determine number within each group
table(vgroups)

## vgroups
## CellCycle  Hedgehog     Notch
##        69        47        62

table(dgroups)

## dgroups
## CellCycle  Hedgehog     Notch
##        91        38       113
```

2 ESTIMATE Analysis

We now run ESTIMATE in order to gauge the estimated stromal and immune components of the three subtypes from the gene expression data.

```
#Run immunoscore on the pancreatic samples and gauge differences between
#subgroups
destimateResults <- runEstimate(dgexp,"ICGC")

## [1] "estimate.zip is stored locally"
## [1] "/Users/chlon01/Desktop/PLOSMED Review Round 2/Code/"
## [1] "This dataset includes 10387genes."
## [1] "25genes were mismatched."
## [1] "1 gene set: StromalSignature  overlap= 141"
## [1] "2 gene set: ImmuneSignature  overlap= 141"

vestimateResults <- runEstimate(vgexp,"TCGA")

## [1] "estimate.zip is stored locally"
## [1] "/Users/chlon01/Desktop/PLOSMED Review Round 2/Code/"
## [1] "This dataset includes 10128genes."
## [1] "284genes were mismatched."
## [1] "1 gene set: StromalSignature  overlap= 137"
## [1] "2 gene set: ImmuneSignature  overlap= 140"

destimateResults <- cbind(destimateResults,subtype = dgroups)
vestimateResults <- cbind(vestimateResults,subtype = vgroups)
immuno <- rbind(destimateResults[,c("ImmuneScore","X5","subtype")],
                vestimateResults[,c("ImmuneScore","X5","subtype")])
immuno[,1] <- as.numeric(immuno[,1])


ggplot(immuno, aes(subtype, ImmuneScore, fill=X5)) + geom_boxplot() +
ggtitle("ESTIMATE Immune Score\n ICGC and TCGA Cohorts")+
  theme(text = element_text(size=18),
      plot.title = element_text(lineheight=.8, face="bold"),
  axis.text.y = element_text(size=13),
  axis.text.x = element_text(size=13))
```

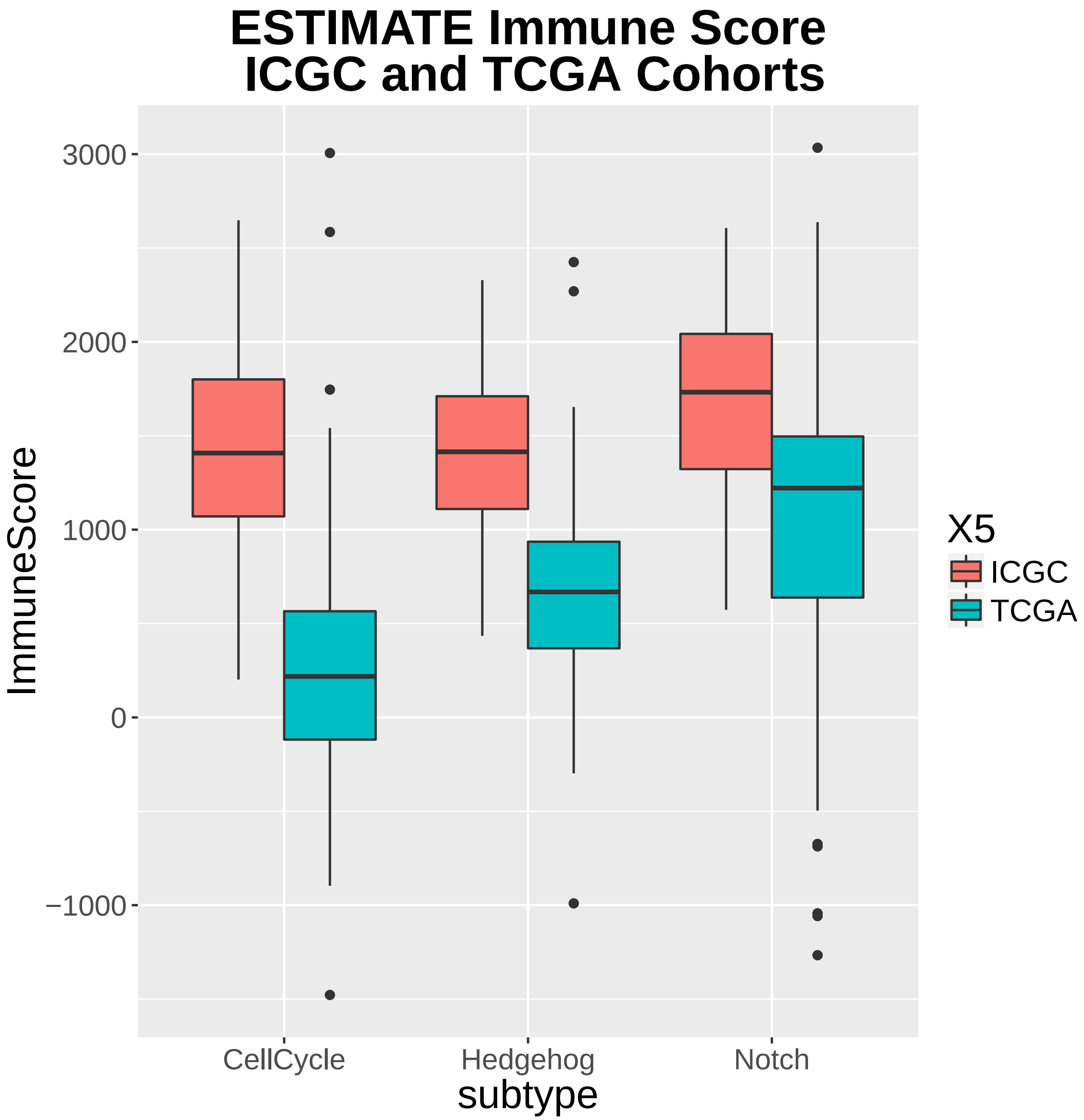


```
stroma <- rbind(destimateResults[,c("StromalScore","X5","subtype")],
                vestimateResults[,c("StromalScore","X5","subtype")])
stroma[,1] <- as.numeric(stroma[,1])
ggplot(stroma, aes(subtype, StromalScore, fill=X5)) + geom_boxplot()+
  ggtitle("ESTIMATE Stromal Score Per Subtype\n ICGC and TCGA Cohorts")+
  theme(text = element_text(size=18),
      plot.title = element_text(lineheight=.8, face="bold"),
  axis.text.y = element_text(size=13),
  axis.text.x = element_text(size=13))
```

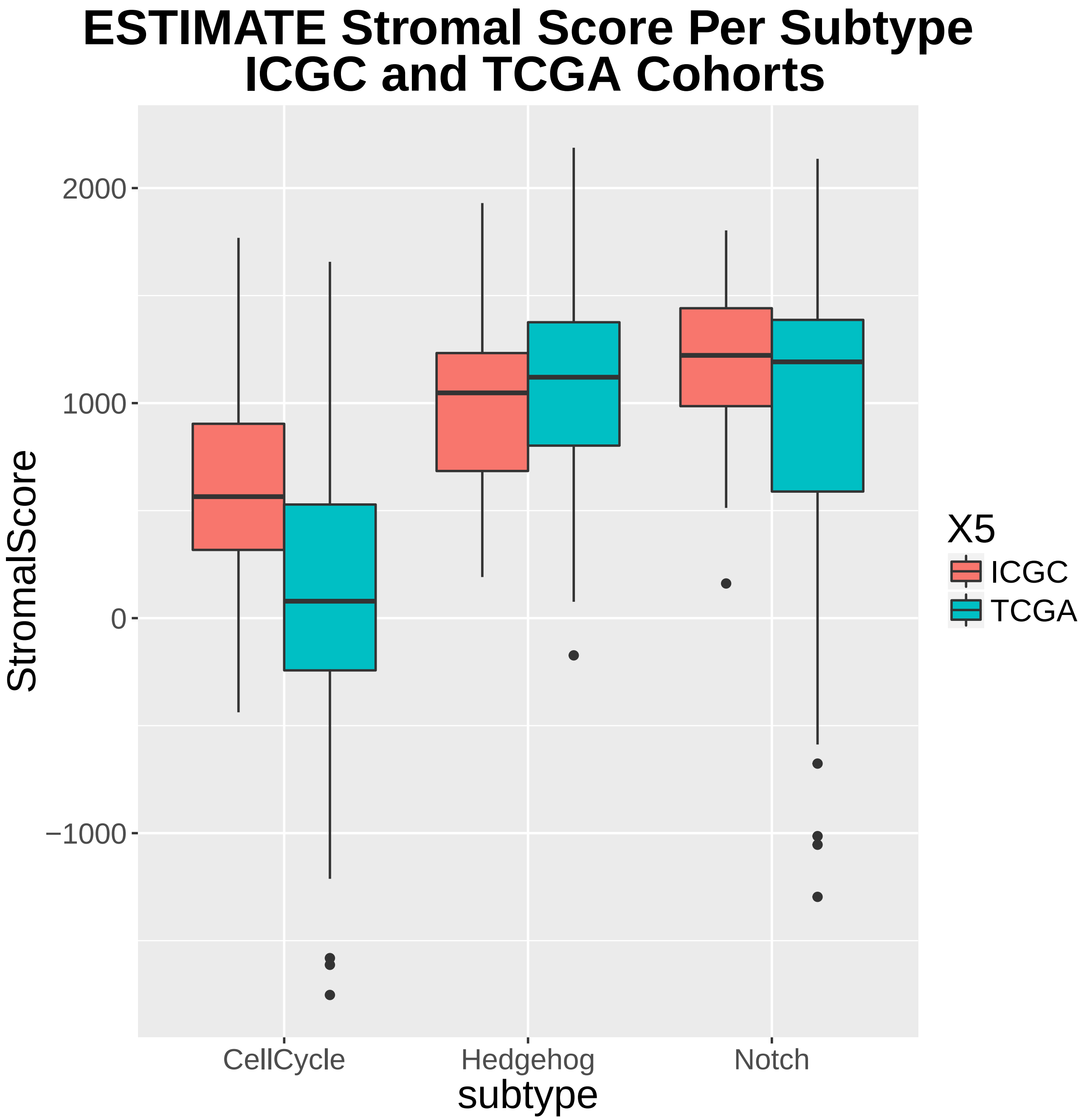

It is evident that Hedgehog and Notch have significantly higher measures of stromal infiltration than CellCycle. It can also be discerned that Notch is the most immunogenic of all three pancreatic subtypes. We confirm this using the Wilcoxon signed-rank test for both the ICGC and TCGA cohorts.

```
####### ICGC Array ######
#General stromal difference by aov
anova(lm(destimateResults$StromalScore ~ dgroups))

## Analysis of Variance Table
##
## Response: destimateResults$StromalScore
##             Df   Sum Sq  Mean Sq F value    Pr(>F)
## dgroups      2 20108129 10054064  67.445 < 2.2e-16 ***
```

```
## Residuals 239 35628096   149072
## ---
## Signif. codes:  0 '***' 0.001 '**' 0.01 '*' 0.05 '.' 0.1 ' ' 1

anova(lm(vestimateResults$ImmuneScore ~ vgroups))

## Analysis of Variance Table
##
## Response: vestimateResults$ImmuneScore
##            Df   Sum Sq Mean Sq F value    Pr(>F)
## vgroups     2 16340585 8170293  14.616 1.349e-06 ***
## Residuals 175 97822886  558988
## ---
## Signif. codes:  0 '***' 0.001 '**' 0.01 '*' 0.05 '.' 0.1 ' ' 1

#Lets determine how immunologically different the Notch and CellCycle groups are
#using a wilcoxon rank sum test

wilcox.test(
  as.numeric(destimateResults$ImmuneScore[destimateResults$subtype == "Notch"]),
  as.numeric(destimateResults$ImmuneScore[destimateResults$subtype == "CellCycle"]))

##
##  Wilcoxon rank sum test with continuity correction
##
## data:  as.numeric(destimateResults$ImmuneScore[destimateResults$subtype ==  and as.numeric(destimateRes
## W = 6947, p-value = 1.658e-05
## alternative hypothesis: true location shift is not equal to 0

#Lets determine how different the stromal content is between the Notch and Hedgehog groups are using a wil
wilcox.test(
  as.numeric(destimateResults$StromalScore[destimateResults$subtype == "Notch"]),
  as.numeric(destimateResults$StromalScore[destimateResults$subtype == "Hedgehog"]))

##
##  Wilcoxon rank sum test with continuity correction
##
## data:  as.numeric(destimateResults$StromalScore[destimateResults$subtype ==  and as.numeric(destimateRe
## W = 2885, p-value = 0.001565
## alternative hypothesis: true location shift is not equal to 0

####### TCGA Array ######
#Lets determine how immunologically different the Notch and CellCycle groups are using a wilcoxon rank sum
wilcox.test(
  as.numeric(vestimateResults$ImmuneScore[vestimateResults$subtype == "Notch"]),
  as.numeric(vestimateResults$ImmuneScore[vestimateResults$subtype == "CellCycle"]))

##
##  Wilcoxon rank sum test with continuity correction
##
## data:  as.numeric(vestimateResults$ImmuneScore[vestimateResults$subtype ==  and as.numeric(vestimateRes
## W = 3305, p-value = 7.755e-08
## alternative hypothesis: true location shift is not equal to 0

#Lets determine how different the stromal content is between the Notch and Hedgehog groups are using a wil
wilcox.test(
  as.numeric(vestimateResults$StromalScore[vestimateResults$subtype == "Notch"]),
  as.numeric(vestimateResults$StromalScore[vestimateResults$subtype == "Hedgehog"]))
```

```
##
##  Wilcoxon rank sum test with continuity correction
##
## data:  as.numeric(vestimateResults$StromalScore[vestimateResults$subtype ==  and as.numeric(vestimateRe
## W = 1451, p-value = 0.9732
## alternative hypothesis: true location shift is not equal to 0
```

Given that Hedgehog and Notch are both strongly associated with stromal infiltration, we examine whether there is any relationship between the pathways. Pathway enrichment is measured using single sample Gene Set Enrichment Analysis (ssGSEA) via the package GSVA with the earlier-derived curated genesets for Hedgehog and Notch.

```
#Single Sample Gene Set Enrichment Analysis
dat <- read.table(lightGet("leo.gmt","data"),
                  stringsAsFactors = F,fill=T,sep="\t")
hog <- c(unlist(dat[78,]), unlist(dat[79,]))
hog <- hog[-c(1,2)]
names(hog) <- NULL
dat <- dat[-c(78,79),]
nams <- dat[,1]
dat <- dat[,-c(1,2)]
ldat <- split(dat, seq_len(nrow(dat)))
nams <- c(nams,"HEDGEHOG")
ldat[[length(ldat) + 1]] = hog
ldat <- lapply(ldat, function(x) x[x != ""])

dmoo <- gsva(as.matrix(dgexp), ldat, method="ssgsea",
            rnaseq=F, verbose=TRUE)
dmoo <- apply(dmoo,2,as.numeric)
rownames(dmoo) <- nams
dstore <- dmoo


vmoo <- gsva(as.matrix(vgexp), ldat, method="ssgsea",
            rnaseq=F, verbose=TRUE)

## Warning in .local(expr, gset.idx.list, ...):  310genes with constant expression values throuhgout
the samples.

vmoo <- apply(vmoo,2,as.numeric)
rownames(vmoo) <- nams
vstore <- vmoo


test = cor.test(as.numeric(dmoo["HEDGEHOG",]),
as.numeric(dmoo["NOTCH",]))
p = test$p.value
if(p == 0){p = "P < 2.2e-16"}else{p = paste("P = ",format(p,digits=4))}
plot(as.numeric(dmoo["HEDGEHOG",]),as.numeric(dmoo["NOTCH",]),
     xlab = "Hedgehog Activity",
     ylab = "Notch Activity",
     main = "ICGC \nHedgehog Activity vs Notch Activity")
abline(lm(as.numeric(dmoo["NOTCH",]) ~ as.numeric(dmoo["HEDGEHOG",])), col='red')
legend("topleft", bty="n",
       legend=paste("r:",format(test$estimate,digits=4),"\n",p),
       cex=1)
```

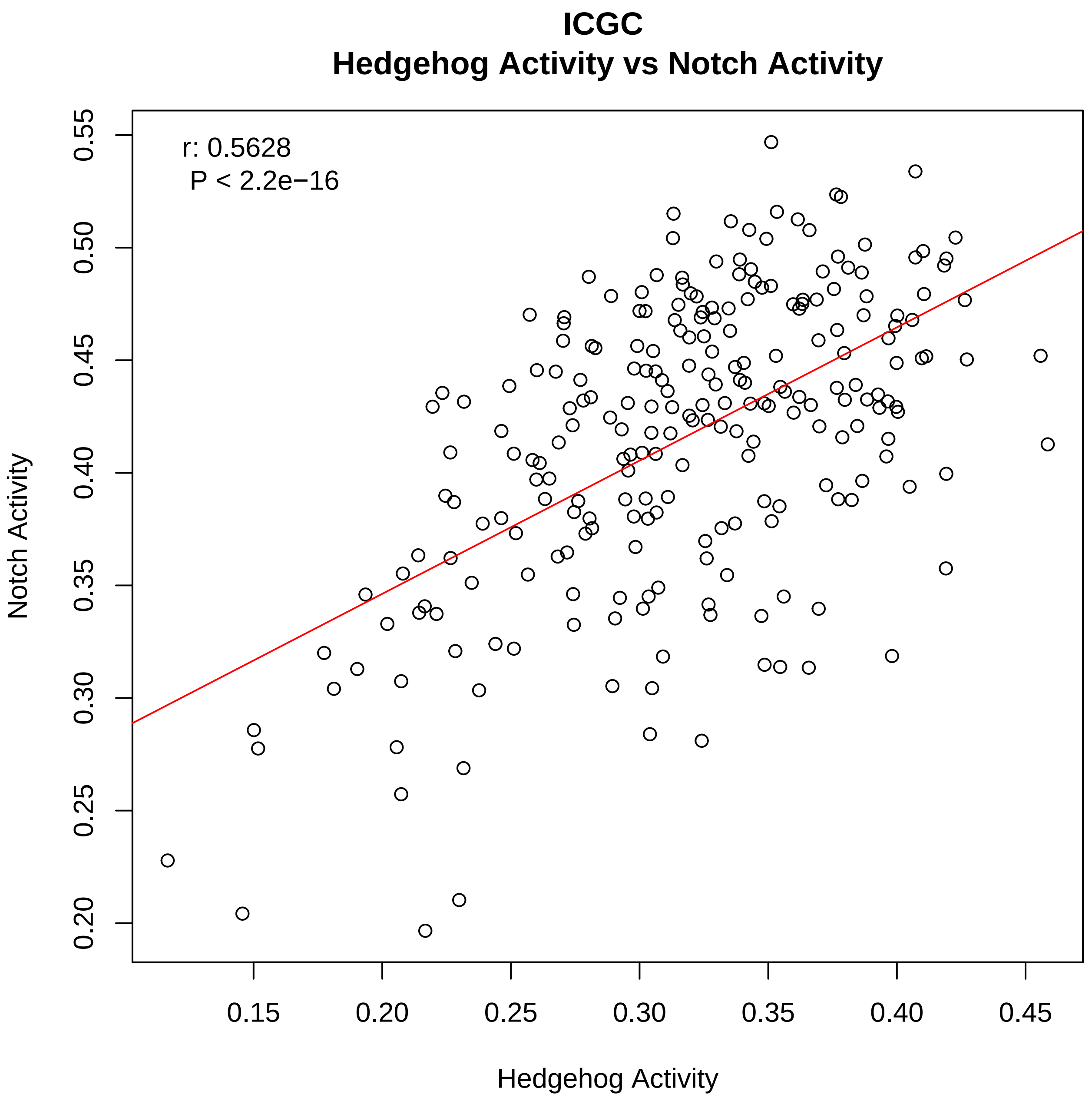


```
test = cor.test(as.numeric(vmoo["HEDGEHOG",]),
as.numeric(vmoo["NOTCH",]))
p = test$p.value
if(p == 0){p = "P < 2.2e-16"}else{p = paste("P = ",format(p,digits=4))}
plot(as.numeric(vmoo["HEDGEHOG",]),as.numeric(vmoo["NOTCH",]),
     xlab = "Hedgehog Activity",
     ylab = "Notch Activity",
     main = "TCGA \nHedgehog Activity vs Notch Activity")
abline(lm(as.numeric(vmoo["NOTCH",]) ~ as.numeric(vmoo["HEDGEHOG",])), col='red')
legend("topleft", bty="n",
       legend=paste("r:",format(test$estimate,digits=4),"\n",p),
       cex=1)
```

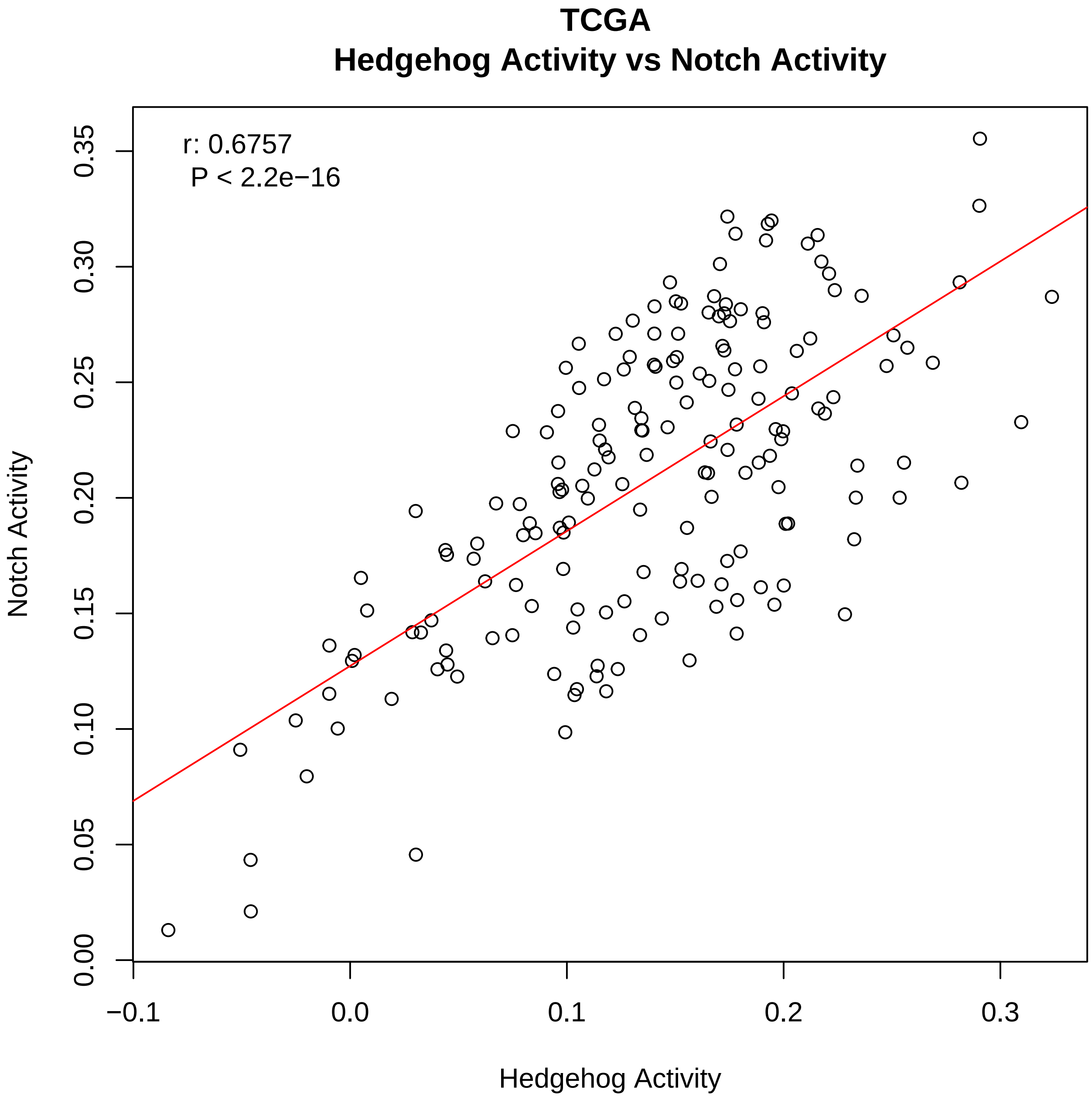

It is clear that there is a strong and significant correlation between the Hedgehog and Notch pathways across both the ICGC and TCGA cohorts.

3 Immunological Pathways Analysis

We now take a set of curated gene sets from the MSigDB filtered for immunological terms in order to gauge how pathway enrichment terms vary given the different pathways. Enrichment of these pathways is measured across each sample using the ssGSEA algorithm via the R package GSVA. We do this using partial correlation studies in order to correct for the influence of the Notch pathway activity over Hedgehog's as demonstrated by the previous correlations. The significance threshold is adjusted by dividing 0.05 by the number of tests in order to correct for multiple testing associated type I errors. The P value threshold is then converted to a T statistic threshold by using the R function "qt" with the number of degrees of freedom being equal to the number of samples in the cohort - 3. The following plots demonstrate the strong affinity of Notch pathway dominated samples towards immunogenic pathways, with T Cell related pathways playing a crucial and consistent role in Notch-dominated microenvironments across both the TCGA and ICGC cohorts.

```
stat = c(0,0)
for(i in 1:(nrow(dmoo)-3))
{
 a = pcor.test(dmoo["NOTCH",],dmoo[i,],dmoo["HEDGEHOG",])
 b = pcor.test(dmoo["HEDGEHOG",],dmoo[i,],dmoo["NOTCH",])
 stat <- rbind(stat,c(a$statistic,b$statistic))
}
stat = stat[-1,]
rownames(stat) = rownames(dmoo)[1:(nrow(dmoo)-3)]
colnames(stat) = c("NOTCH","HEDGEHOG")


thresh = qt(p = 0.025/160,df = 239,lower.tail = F)
dstat = stat[abs(stat[,1]) > thresh | abs(stat[,2]) > thresh,]
fail1 = dstat[abs(dstat[,2]) < thresh,2]
failID1 = names(fail1)[grep("T_CELL",names(fail1))]
overall = data.frame(Notch = dstat[failID1,1], Hedgehog = dstat[failID1,2])
mdat <- melt(as.matrix(overall), id.vars=rownames())
colnames(mdat) <- c("Pathway", "Type", "Metric")
mdat = cbind(mdat, d2 = rep(thresh,length(failID1)))

jpeg('plot1.jpg',width = 1000,height = 1000)
ggplot(data = mdat,aes(Pathway)) +
    geom_bar(aes(y=Metric, fill=Type), stat="identity", position="dodge") +
    geom_line(aes(y=d2,group=1,color="Significance\nThreshold"),linetype="dotted") +
    geom_line(aes(y=-d2,group=1,color="Significance\nThreshold"),linetype="dotted") +
  scale_colour_manual(" ", values=c("Significance\nThreshold" = "black"))+
    ggtitle("ICGC\n T Cell Related Pathways Notch/Hedgehog") +
theme(text = element_text(size=18),
  plot.title = element_text(lineheight=.8, face="bold"),
  axis.text.y = element_text(angle = 0,size=13),
  axis.text.x = element_text(angle = 0,size=13),
  legend.key=element_blank(),
          legend.title=element_blank()) +
  ylab("T Statistic")+
      coord_flip()
dev.off()

## pdf
##   2
```

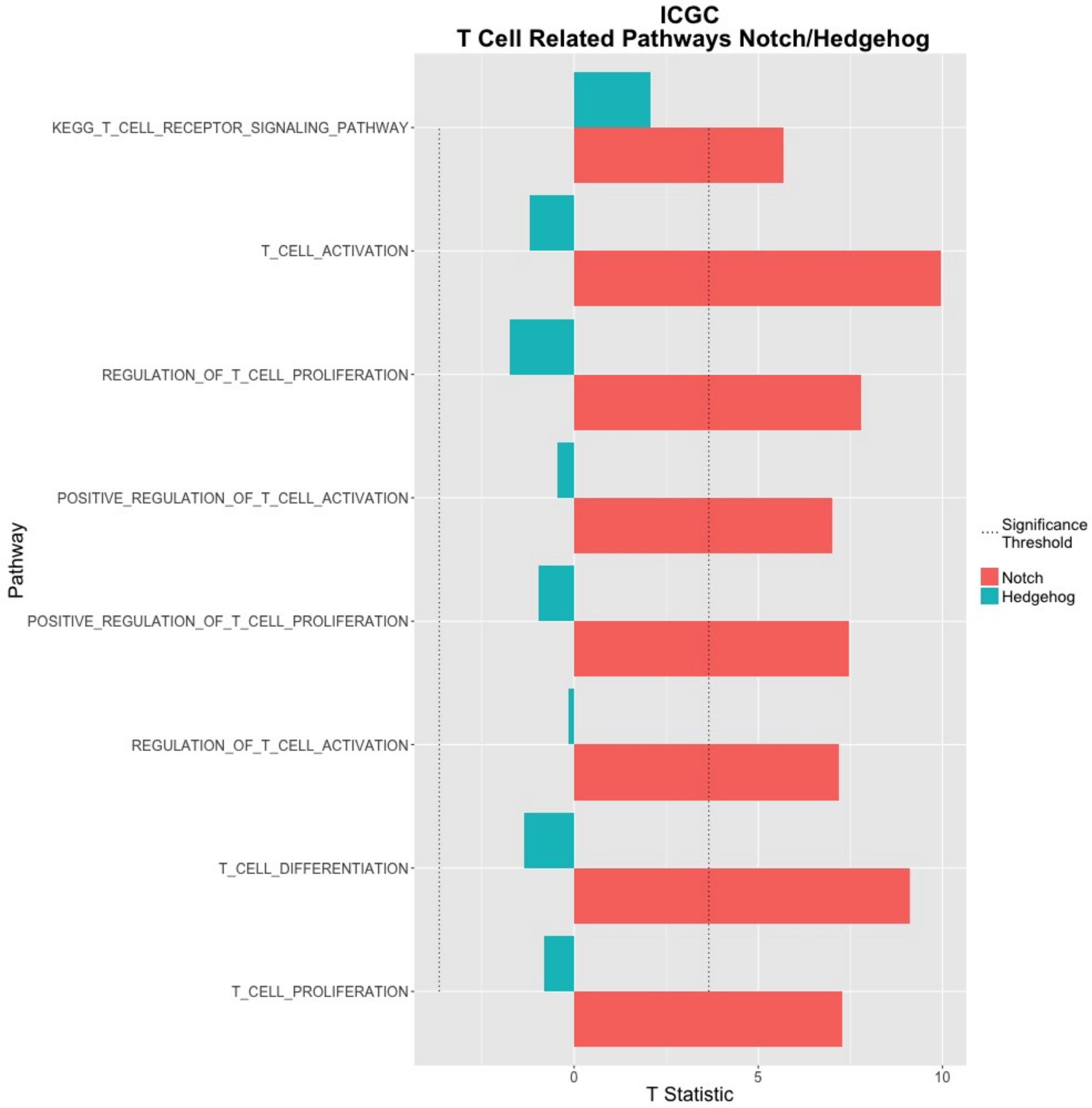


```
stat = c(0,0)
for(i in 1:(nrow(vmoo)-3))
{
 a = pcor.test(vmoo["NOTCH",],vmoo[i,],vmoo["HEDGEHOG",])
 b = pcor.test(vmoo["HEDGEHOG",],vmoo[i,],vmoo["NOTCH",])
 stat <- rbind(stat,c(a$statistic,b$statistic))
}
stat = stat[-1,]
rownames(stat) = rownames(vmoo)[1:(nrow(vmoo)-3)]
colnames(stat) = c("NOTCH","HEDGEHOG")
thresh = qt(p = 0.025/160,df = 175,lower.tail = F)
vstat = stat[abs(stat[,1]) > thresh | abs(stat[,2]) > thresh,]
fail2 = vstat[abs(vstat[,2]) < thresh,2]
failID2 = names(fail2)[grep("T_CELL",names(fail2))]

overall = data.frame(Notch = vstat[failID2,1], Hedgehog = vstat[failID2,2])
```

```
mdat <- melt(as.matrix(overall), id.vars=rownames())
colnames(mdat) <- c("Pathway", "Type", "Metric")
mdat = cbind(mdat, d2 = rep(thresh,length(failID2)))

jpeg('plot2.jpg',width = 1000,height = 1000)
ggplot(data = mdat,aes(Pathway)) +
    geom_bar(aes(y=Metric, fill=Type), stat="identity", position="dodge") +
    geom_line(aes(y=d2,group=1,color="Significance\nThreshold"),linetype="dotted") +
    geom_line(aes(y=-d2,group=1,color="Significance\nThreshold"),linetype="dotted") +
  scale_colour_manual(" ", values=c("Significance\nThreshold" = "black"))+
    ggtitle("TCGA \nT Cell Related Pathways Notch/Hedgehog") +
theme(text = element_text(size=18),
  plot.title = element_text(lineheight=.8, face="bold"),
  axis.text.y = element_text(angle = 0,size=13),
  axis.text.x = element_text(angle = 0,size=13),
  legend.key=element_blank(),
          legend.title=element_blank()) +
  ylab("T Statistic")+
      coord_flip()
dev.off()

## pdf
##   2
```

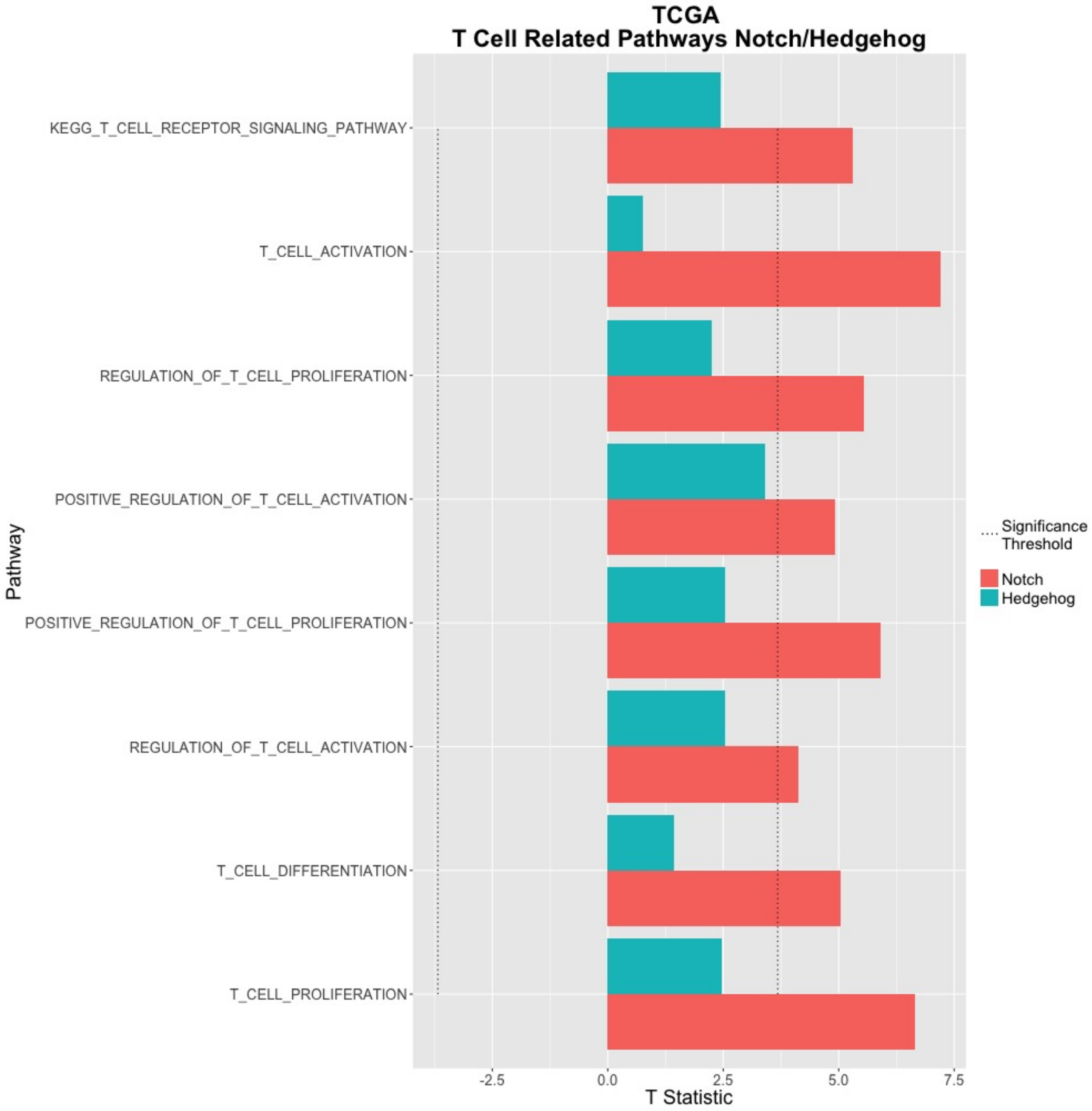


```
intersected = intersect(rownames(vstat), rownames(dstat))


overall = cbind(Disc = dstat[intersected,1] - dstat[intersected,2],
                Val= (vstat[intersected,1] - vstat[intersected,2]))

overall = overall[(overall[,1] * overall[,2]) > 0,]
overall = overall[order(overall[,1]),]
overall = overall[abs(overall[,1]) >= quantile(abs(overall[,1]),0.5),]

colnames(overall) = c("ICGC","TCGA")
mdat <- melt(overall, id.vars=rownames())
colnames(mdat) <- c("Pathway", "Cohort", "Metric")
jpeg('plot3.jpg',width = 1000,height = 1000)
ggplot(mdat, aes(Pathway, Metric, fill=Cohort)) +
    geom_bar(stat="identity", position="dodge") +
```

```
    coord_flip() + ggtitle("ssGSEA Analysis\nHedgehog
theme(text = element_text(size=18),
  plot.title = element_text(lineheight=.8, face="bold"),
  axis.text.y = element_text(angle = 0,size=13),
  axis.text.x = element_text(size=13)) +
  ylab("T Statistic Difference")
dev.off()

## pdf
##   2
```

The following plot is produced by isolating associations between Hedgehog or Notch that fall below the significance threshold and the metric plotted is the difference between the t statistic calculated for Notch and that calculated for Hedgehog for all such significant pathways.

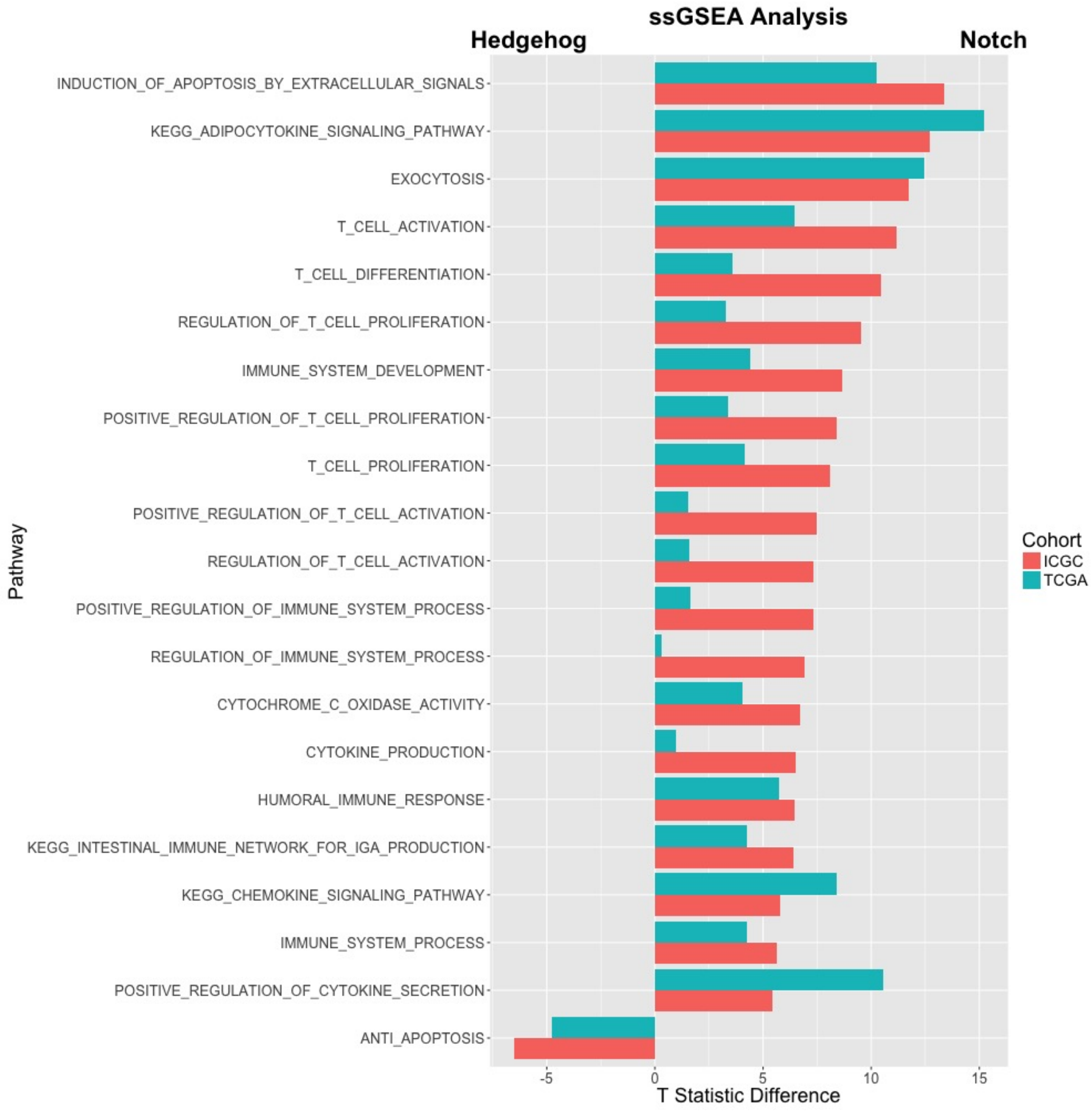

4 CIBERSORT Analysis

We now use CIBERSORT in order to gauge the estimated proportions of different leukocyte families within the tumour microenvironment. The TCGA cohort is in the form of RNA-seq derived gene expression data which has been normalised via the Variance Stabilising Transform (VST). This is done to transform its underlying gene expression distribution to that of the microarray-derived leukocyte signatures used in CIBERSORT's deconvolution algorithm. CIBERSORT is run with 1000 permutations and a cutoff of p ¡ 0.05 is taken to mean the sample's deconvolution was successful.

```
#Run CIBERSORT and infer estimated populations of leukocytes
dciberResults <- runCibersort(dgexp,"pancreatic",
                              lightGet("LM22.txt","data"))

## [1] "Cibersort.R is stored locally"
## [1] "LM22.txt is stored locally"
##     user   system  elapsed
## 1122.550   89.084  570.960

dciberResults <- cbind(dciberResults,subtype = dgroups)
dciberResults <- dciberResults[order(rownames(dciberResults)),]

vciberResults <- runCibersort(vgexp,"pancreatic",
                              lightGet("LM22.txt","data"))

## [1] "Cibersort.R is stored locally"
## [1] "LM22.txt is stored locally"
##    user  system elapsed
## 935.208 100.447 503.720

vciberResults <- vciberResults[order(rownames(vciberResults)),]
vciberResults <- cbind(vciberResults,subtype = vgroups)
vestimateResults = vestimateResults[order(vestimateResults$NAME),]
```

After running CIBERSORT, we look at whether or not CIBERSORT's "correlation" metric for how well a deconvolution went compares against the immunescore of each sample. The following plots show that there is a strong agreement between the ability of CIBERSORT to deconvolve a sample and the estimated immune content of that sample.

```
#Gauge agreement between immunoscore and cibersort
test = cor.test(as.numeric(destimateResults$ImmuneScore),
                as.numeric(dciberResults$Correlation))
plot(as.numeric(destimateResults$ImmuneScore),
     as.numeric(dciberResults$Correlation),
     main= "ICGC Cohort \n ImmunoScore vs CIBERSORT correlation")
legend("topleft", bty="n",
       legend=paste("r:",format(test$estimate,digits=4)))
```

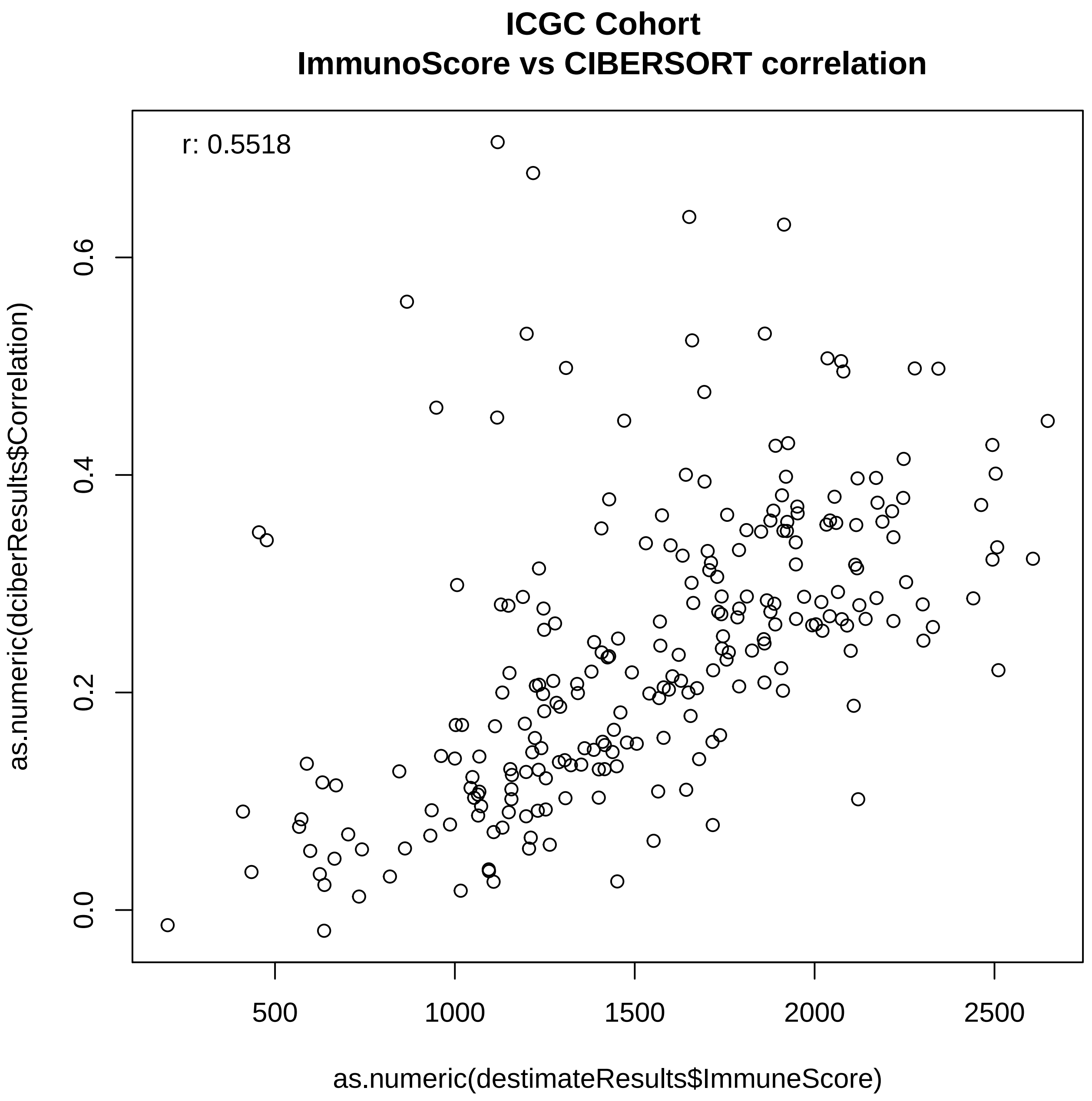


```
test = cor.test(as.numeric(vestimateResults$ImmuneScore),
                as.numeric(vciberResults$Correlation))
plot(as.numeric(vestimateResults$ImmuneScore),
     as.numeric(vciberResults$Correlation),
     main= "TCGA Cohort \n ImmunoScore vs CIBERSORT correlation")
legend("topleft", bty="n",
       legend=paste("r:",format(test$estimate,digits=4)))
```

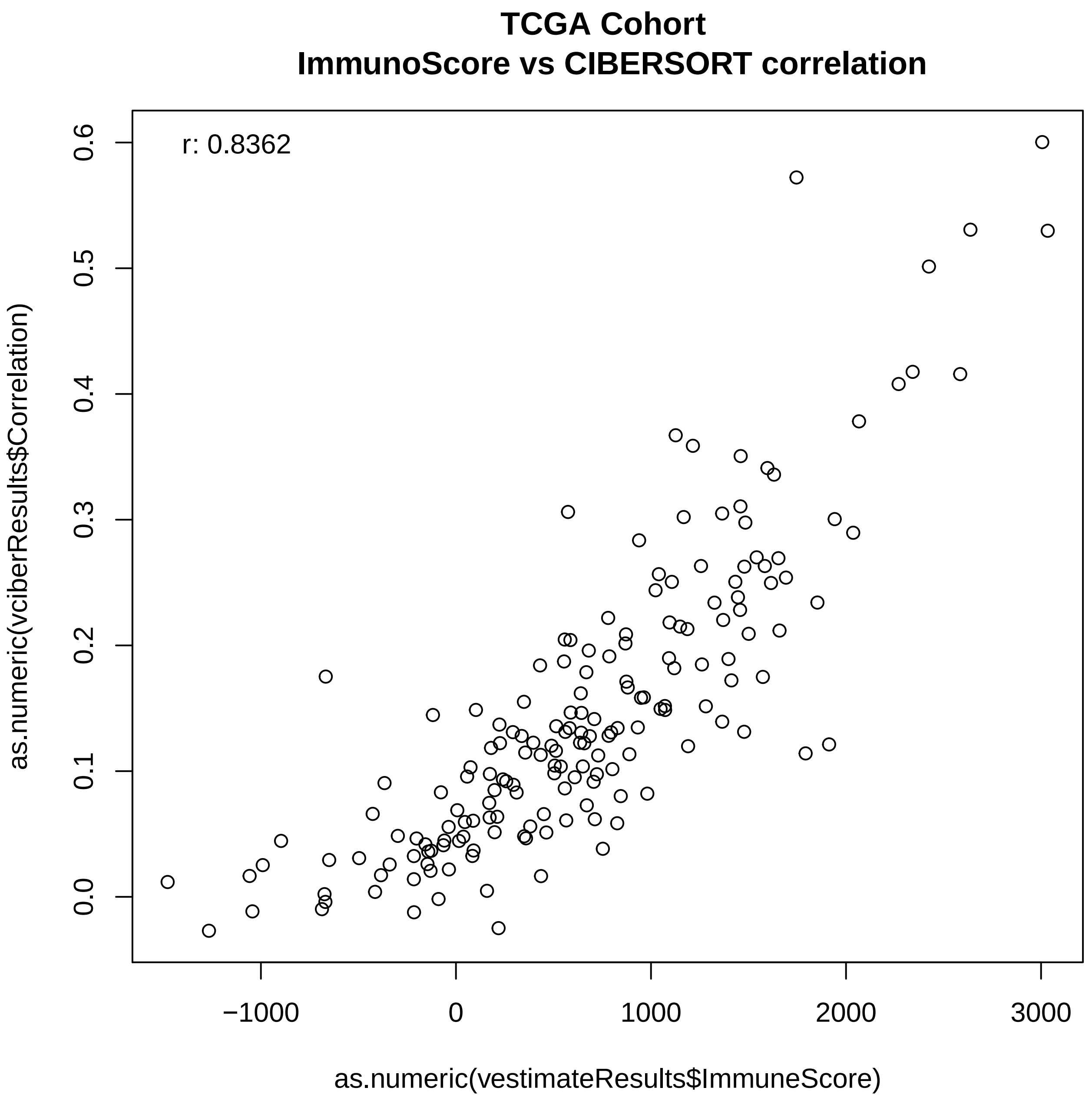

The following plot demonstrates the strong affinity of Notch pathway dominated samples towards CD8 T cells. The significance threshold is adjusted by dividing 0.05 by the number of tests done in each cohort in order to correct for multiple testing associated type I errors. The significant results found in the ICGC cohort were investigated in the TCGA cohort which meant that fewer tests were performed in the latter. The P value threshold is then converted to a T statistic threshold by using the R function "qt" with the number of degrees of freedom being equal to the number of samples in the cohort - 3.The plot is produced by isolating associations between Hedgehog or Notch that fall below the significance threshold and the metric plotted is the difference between the t statistic calculated for Notch and that calculated for Hedgehog.

```
#Let us correlate the cell type population with the HEDGEHOG and NOTCH activity scores in order to deduce

#First we examine the discovery set
```

```
dciberResults <- dciberResults[dciberResults$P.value <= 0.05,]
dciberResults <- dciberResults[,1:20]
dmoo <- dmoo[,colnames(dmoo) %in% rownames(dciberResults)]
all(colnames(dmoo) == rownames(dciberResults))

## [1] TRUE

dciberResults <- dciberResults[,!(colSums(dciberResults) == 0)]

#Remove ambiguous leukocyte from the results matrix
neg = which(colnames(dciberResults) == "Macrophages.M0")

dciberResults = dciberResults[,-neg]


stat = c(0,0)
for(i in 1:ncol(dciberResults))
{
 vec = dciberResults[,i]
 outUp = mean(vec) + sd(vec) * 3
 outDown  = mean(vec) - sd(vec) * 3
 neg = vec > outUp | vec < outDown
 a = pcor.test(vec[!neg],dmoo["NOTCH",!neg],dmoo["HEDGEHOG",!neg],
              method="spearman")
 b = pcor.test(vec,dmoo["HEDGEHOG",],dmoo["NOTCH",],
              method="spearman")
 stat = rbind(stat,c(a$statistic,b$statistic))
}
dpvals <- stat[-1,]
rownames(dpvals) = colnames(dciberResults)
colnames(dpvals) = c("Notch","Hedgehog")
thresh = qt(0.025/40,(nrow(dciberResults) - 3),lower.tail = F)
dpvals = dpvals[abs(dpvals[,1]) > thresh | abs(dpvals[,2]) > thresh,]

#And now the validation set
vciberResults <- vciberResults[vciberResults$P.value <= 0.05,]
vciberResults <- vciberResults[,1:20]
neg = which(colnames(vciberResults) == "Macrophages.M0")
vciberResults = vciberResults[,-neg]
vmoo <- vmoo[,colnames(vmoo) %in% rownames(vciberResults)]
vmoo <- vmoo[,order(colnames(vmoo))]
vciberResults <- vciberResults[order(rownames(vciberResults)),]
all(colnames(vmoo) == rownames(vciberResults))

## [1] TRUE

vciberResults <- vciberResults[,!(colSums(vciberResults) == 0)]
stat = c(0,0)
for(i in 1:ncol(vciberResults))
{
   vec = vciberResults[,i]
   outUp = mean(vec) + sd(vec) * 3
   outDown  = mean(vec) - sd(vec) * 3
   neg = vec > outUp | vec < outDown
 a = pcor.test(vmoo["NOTCH",!neg],vec[!neg],vmoo["HEDGEHOG",!neg],
```

```
               method="spearman")
 b = pcor.test(vmoo["HEDGEHOG",!neg],vec[!neg],vmoo["NOTCH",!neg],
               method="spearman")
stat = rbind(stat,c(a$statistic,b$statistic))
}
## Warning in pcor(xyz, method = method):  The inverse of variance-covariance matrix is calculated
using Moore-Penrose generalized matrix invers due to its determinant of zero.
## Warning in cov2cor(icvx):  diag(.)  had 0 or NA entries; non-finite result is doubtful
## Warning in pcor(xyz, method = method):  The inverse of variance-covariance matrix is calculated
using Moore-Penrose generalized matrix invers due to its determinant of zero.
## Warning in cov2cor(icvx):  diag(.)  had 0 or NA entries; non-finite result is doubtful
## Warning in pcor(xyz, method = method):  The inverse of variance-covariance matrix is calculated
using Moore-Penrose generalized matrix invers due to its determinant of zero.
## Warning in cov2cor(icvx):  diag(.)  had 0 or NA entries; non-finite result is doubtful
## Warning in pcor(xyz, method = method):  The inverse of variance-covariance matrix is calculated
using Moore-Penrose generalized matrix invers due to its determinant of zero.
## Warning in cov2cor(icvx):  diag(.)  had 0 or NA entries; non-finite result is doubtful
vpvals <- stat[-1,]
rownames(vpvals) = colnames(vciberResults)
colnames(vpvals) = c("Notch","Hedgehog")
#Validate samples we are interested in
vpvals = vpvals[rownames(dpvals),]
vpvals = vpvals[!is.na(rowSums(vpvals)),]
thresh = qt(0.025,(nrow(vciberResults) - 3),lower.tail = F)


cellID = rownames(vpvals[which(abs(vpvals[,1]) >= thresh | abs(vpvals[,2]) >= thresh),])
vpvals = vpvals[cellID,]
dpvals = dpvals[cellID,]
mdat = data.frame(Cohort = c(rep("ICGC",nrow(dpvals)),rep("TCGA",nrow(vpvals))),
                  Cell = c(rownames(dpvals),rownames(vpvals)),
                  value = c(dpvals[,1] - dpvals[,2], vpvals[,1] - vpvals[,2]),
                  stringsAsFactors = F)


ggplot(mdat, aes(Cell, value, fill=Cohort)) +
    geom_bar(stat="identity", position="dodge") +
    coord_flip() + ggtitle("CIBERSORT Analysis") +
theme(text = element_text(size=18),
  plot.title = element_text(lineheight=.8, face="bold"),
  axis.text.y = element_text(angle = 0,size=13),
  axis.text.x = element_text(size=13)) +
  ylab("T-Statistic Difference")
```

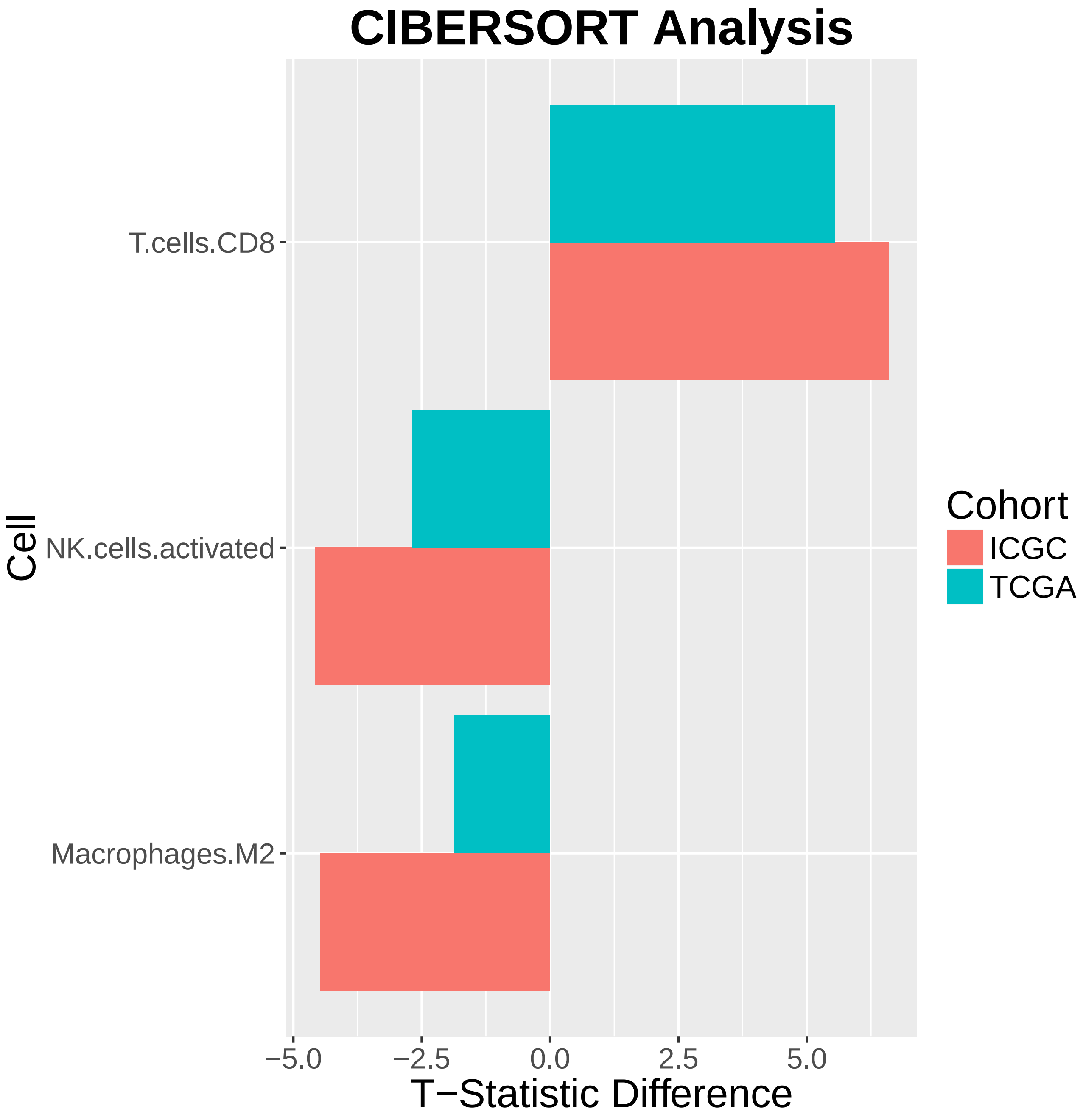
CIBERSORT Analysis
T.cells.CD8
NK.cells.activated
Macrophages.M2
Cell
−5.0
−2.5
0.0
2.5
5.0
T−Statistic Difference
Cohort
ICGC
TCGA

5 Immune Checkpoint Analysis

We now investigate the association between the Hedgehog and Notch pathways and the immune checkpoint markers PD-1, PD-L1 and CLTA-4.

```
rnams <- rownames(dgexp)
dgexp <- apply(dgexp,2,as.numeric)
rownames(dgexp) <- rnams
all(colnames(dgexp) == colnames(dstore))

## [1] TRUE

genes = c("CD274", "CTLA4", "PDCD1", "LAG3", "TIGIT")
store = c(0,0)
for(i in 1:length(genes)){
a = pcor.test(dstore["HEDGEHOG",],as.numeric(dgexp[genes[i],]),
              dstore["NOTCH",])$statistic
b = pcor.test(dstore["NOTCH",],as.numeric(dgexp[genes[i],]),
          dstore["HEDGEHOG",])$statistic
store = rbind(store,c(b,a))
}
store = store[-1,]
rownames(store) = genes
colnames(store) = c("Notch","Hedgehog")
thresh = qt(p = 0.025/(2 * length(genes)),df = 239,lower.tail = F)
daccept = store[abs(store[,1]) > thresh | abs(store[,2]) > thresh,]

mdat <- melt(as.matrix(daccept), id.vars=rownames())
colnames(mdat) <- c("Immune_Checkpoint", "Type", "Metric")
mdat = cbind(mdat, d2 = rep(thresh,length(daccept)))

jpeg("plot4.jpg")
ggplot(data = mdat,aes(Immune_Checkpoint)) +
    geom_bar(aes(y=Metric, fill=Type), stat="identity", position="dodge") +
    geom_line(aes(y=d2,group=1,color="Significance\nThreshold"),linetype="dotted",size=1.5) +
    geom_line(aes(y=-d2,group=1,color="Significance\nThreshold"),linetype="dotted",size=1.5) +
  scale_colour_manual(" ", values=c("Significance\nThreshold" = "black"))+
    ggtitle("ICGC Immune Checkpoint Expression in \nNotch/Hedgehog") +
theme(text = element_text(size=18),
  plot.title = element_text(lineheight=.8, face="bold"),
  axis.text.y = element_text(angle = 0,size=13),
  axis.text.x = element_text(angle = 0,size=13),
  legend.key=element_blank(),
          legend.title=element_blank()) +
  ylab("T Statistic")+
      coord_flip()
dev.off()

## pdf
##   2

store = c(0,0)
for(i in 1:length(genes)){
a = pcor.test(vstore["HEDGEHOG",],as.numeric(vgexp[genes[i],]),
              vstore["NOTCH",])$statistic
b = pcor.test(vstore["NOTCH",],as.numeric(vgexp[genes[i],]),
```

```
          vstore["HEDGEHOG",])$statistic
store = rbind(store,c(b,a))
}
store = store[-1,]
rownames(store) = genes
colnames(store) = c("Notch","Hedgehog")
thresh = qt(p = 0.025/(2 * length(genes)),df = 175,lower.tail = F)
vaccept = store[abs(store[,1]) > thresh | abs(store[,2]) > thresh,]

mdat <- melt(as.matrix(vaccept), id.vars=rownames())
colnames(mdat) <- c("Immune_Checkpoint", "Type", "Metric")
mdat = cbind(mdat, d2 = rep(thresh,length(vaccept)))

jpeg("plot5.jpg")
ggplot(data = mdat,aes(Immune_Checkpoint)) +
    geom_bar(aes(y=Metric, fill=Type), stat="identity", position="dodge") +
    geom_line(aes(y=d2,group=1,color="Significance\nThreshold"),linetype="dotted",size=1.5) +
    geom_line(aes(y=-d2,group=1,color="Significance\nThreshold"),linetype="dotted",size=1.5) +
  scale_colour_manual(" ", values=c("Significance\nThreshold" = "black"))+
    ggtitle("TCGA Immune Checkpoint Expression in \nNotch/Hedgehog") +
theme(text = element_text(size=18),
  plot.title = element_text(lineheight=.8, face="bold"),
  axis.text.y = element_text(angle = 0,size=13),
  axis.text.x = element_text(angle = 0,size=13),
  legend.key=element_blank(),
          legend.title=element_blank()) +
  ylab("T Statistic")+
      coord_flip()
dev.off()

## pdf
##   2
```

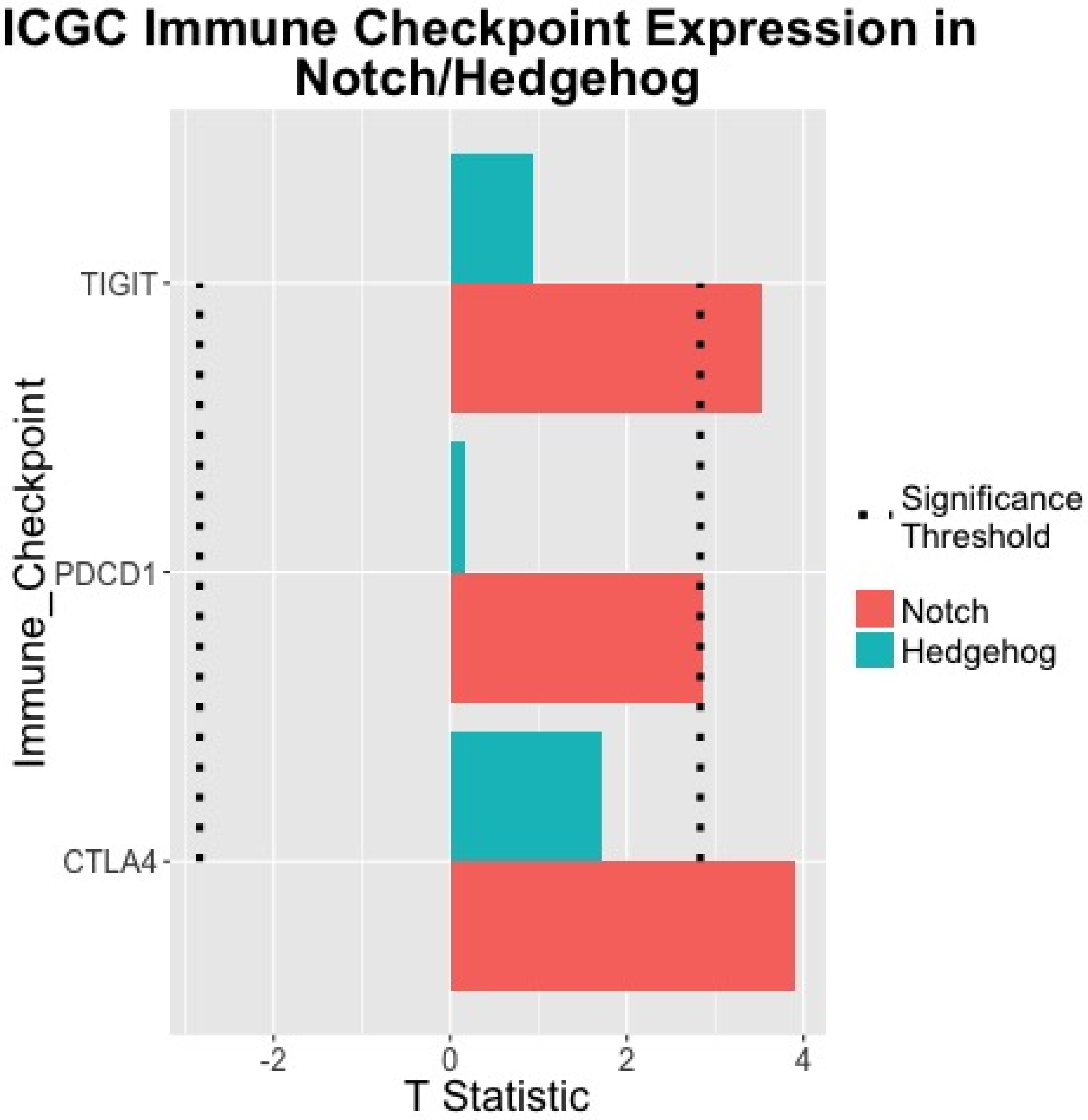
ICGC Immune Checkpoint Expression in Notch/Hedgehog
TIGIT
PDCD1
CTLA4
Immune_Checkpoint
-2
0
2
4
T Statistic
Significance Threshold
Notch
Hedgehog

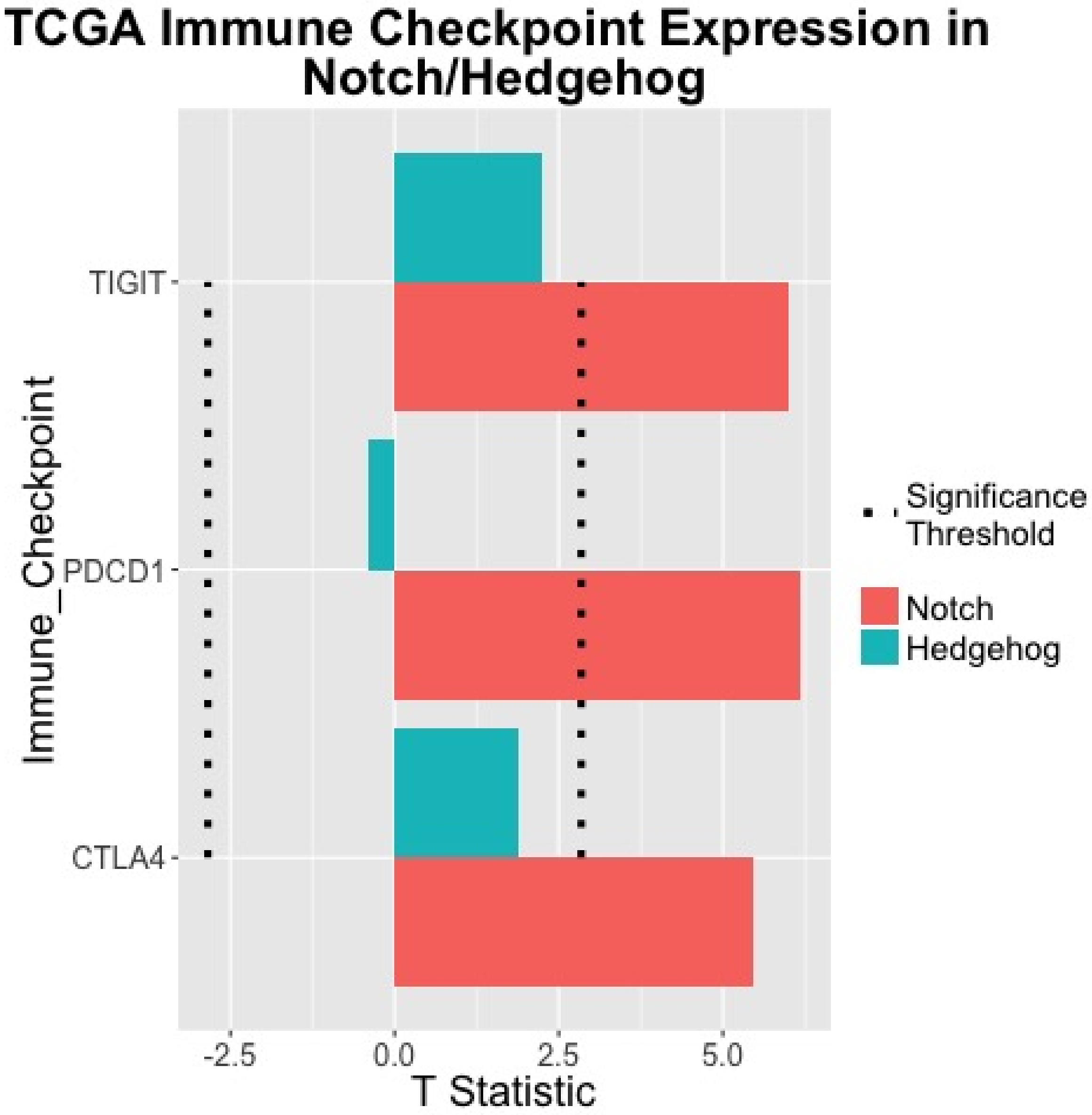

It is evident that there is a significant and consistent association between the immune checkpoint markers PD-1, PD-L1 and CLTA-4 and Notch pathway dominated tumours in both the ICGC and TCGA cohorts. There is no significant association between these markers and Hedgehog pathway dominated tumours.

6 Cancer Cell Line Analysis

44 pancreatic cancer cell lines were downloaded from the Broad Institute Cancer Cell Line Encyclopaedia. These were classified into Hedgehog, CellCycle or Notch by means of the nearest shrunken centroids method implemented via the R package pamr. This was then verified by comparing the subtype classification with each cell lines ssGSEA-derived enrichment score for the Hedgehog, CellCycle and Notch pathway. A barplot shows the enrichment scores for these pathways in each of the cell lines whilst the shrunken centroids classification results are printed as a table.

```
#Run immunoscore on the pancreatic samples and gauge differences between subgroups
ccl <- read.table(lightGet("cclExp.txt","data"),sep="\t",header=T)

## [1] "cclExp.txt is stored locally"

estimateResults <- runEstimate(ccl,"pancreatic")

## [1] "estimate.zip is stored locally"
## [1] "/Users/chlon01/Desktop/PLOSMED Review Round 2/Code/"
## [1] "This dataset includes 10143genes."
## [1] "269genes were mismatched."
## [1] "1 gene set: StromalSignature  overlap= 139"
## [1] "2 gene set: ImmuneSignature  overlap= 139"

toPlot = data.frame(ImmuneScore = as.numeric(estimateResults$ImmuneScore),
                    StromalScore = as.numeric(estimateResults$StromalScore))
boxplot(toPlot,ylab = "ESTIMATE output")
```

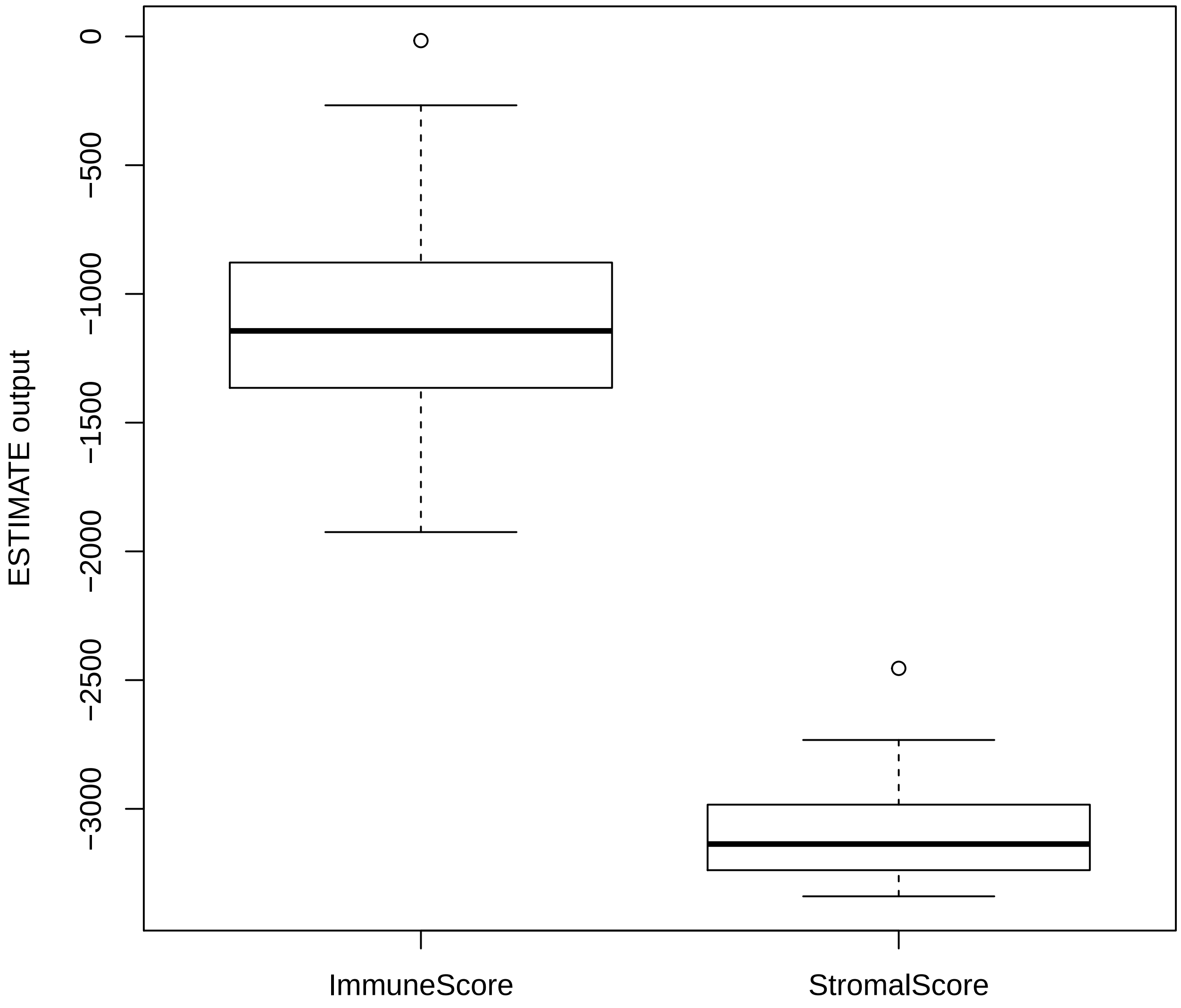


```
#Run ssGSEA to examine enrichment of the Hedgehog Cellcycle and Notch
#Pathways for all pancreatic cell lines
moo <- gsva(as.matrix(ccl), ldat, method="ssgsea", rnaseq=F, verbose=F)
moo <- apply(moo,2,as.numeric)
rownames(moo) <- nams
classes1 <- moo[c("HEDGEHOG","NOTCH","CELLCYCLE"),]
mdat <- melt(classes1, id.vars=rownames())
colnames(mdat) <- c("Pathway", "Cell_Line", "ssGSEA_activity")
ggplot(mdat, aes(Cell_Line, ssGSEA_activity, fill=Pathway)) +
    geom_bar(stat="identity", position="dodge") +
    ggtitle("ssGSEA Analysis\n Cell Line Pathway Association") +
theme(axis.text.x = element_text(angle = 90, hjust = 1),
```

```
    plot.title = element_text(lineheight=.8, face="bold")) +
  ylab("ssGSEA enrichment score")+
  theme(text = element_text(size=18),
    plot.title = element_text(lineheight=.8, face="bold"),
  axis.text.y = element_text(size=13),
  axis.text.x = element_text(size=13))
```

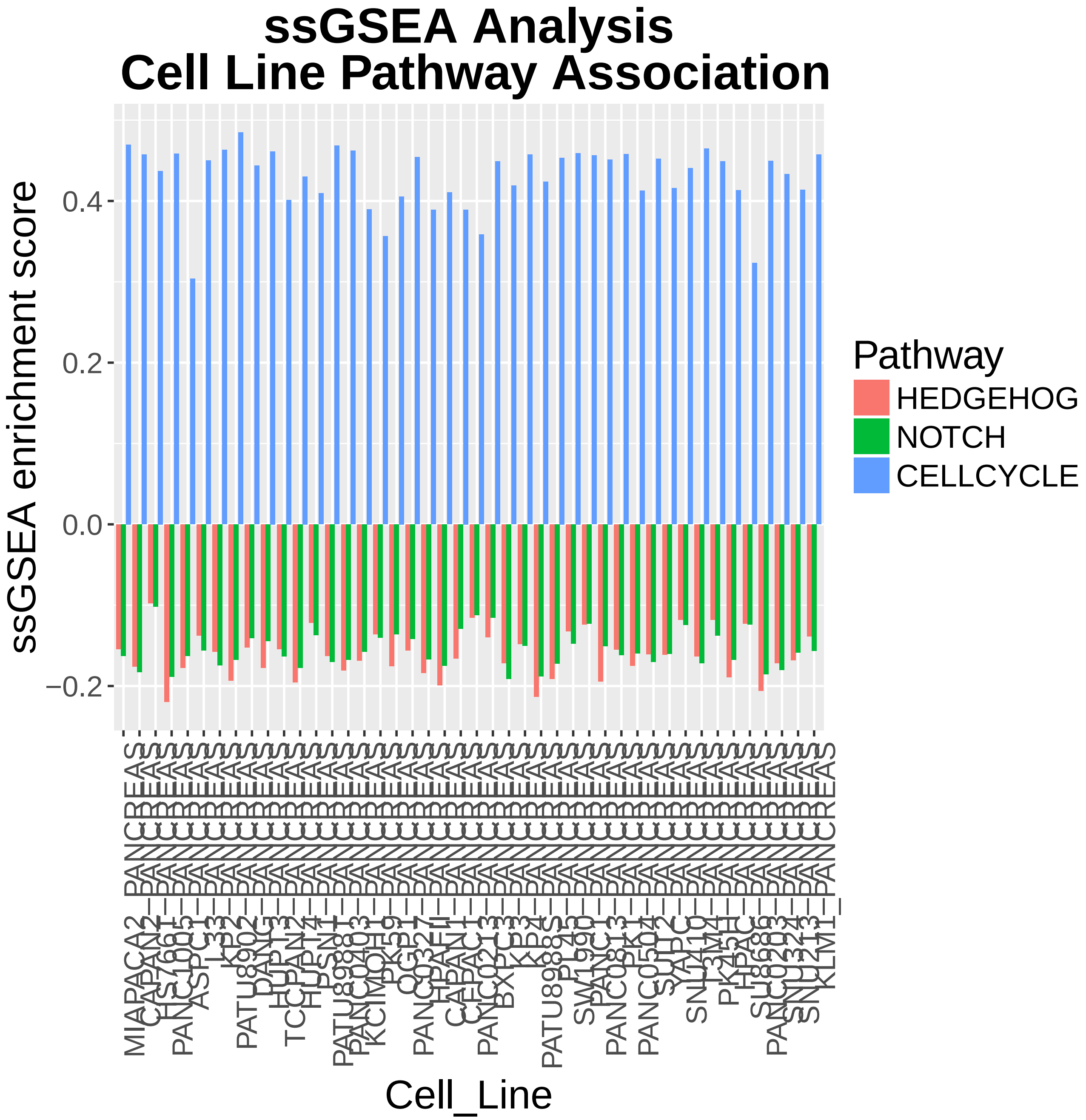


```
gexp <- dgexp
groups = tgroups_Grimm
rows = rownames(gexp)
cols = colnames(gexp)
gexp <- apply(gexp,2,scale)
```

```
rownames(gexp) = rows
colnames(gexp) = cols
gexp <- gexp[rownames(gexp) %in% rownames(ccl),]
ccl <- ccl[rownames(ccl) %in% rownames(gexp),]
ccl <- ccl[order(rownames(ccl)),]
gexp <- gexp[order(rownames(gexp)),]
all(rownames(gexp) == rownames(ccl))

## [1] TRUE

data = list(x= gexp, y= groups, geneids= rownames(gexp),
            samples=colnames(gexp))
#Generate shrunken centroids for each subtype from ICGC data
pamObj = pamr.train(data)

## 123456789101112131415161718192021222324252627282930

#Predict the classes for each cell line and tabulate the results
table(pamr.predict(pamObj,ccl,
                   pamObj$threshold[pamObj$errors == min(pamObj$errors)][1],
                   type = "class"))

##
## CellCycle  Hedgehog     Notch
##        44         0         0
```

```
print(sessionInfo())

## R version 3.2.2 (2015-08-14)
## Platform: x86_64-apple-darwin13.4.0 (64-bit)
## Running under: OS X 10.11.3 (El Capitan)
##
## locale:
## [1] en_GB.UTF-8/en_GB.UTF-8/en_GB.UTF-8/C/en_GB.UTF-8/en_GB.UTF-8
##
## attached base packages:
## [1] parallel  stats     graphics  grDevices utils     datasets  methods   base
##
## other attached packages:
##  [1] preprocessCore_1.32.0 e1071_1.6-7           estimate_1.0.11
##  [4] pamr_1.55             survival_2.38-3       cluster_2.0.3
##  [7] reshape2_1.4.1        ggplot2_2.1.0         ppcor_1.1
## [10] MASS_7.3-45           GSVA_1.16.0           knitr_1.11
##
## loaded via a namespace (and not attached):
##  [1] Rcpp_0.12.4          formatR_1.2.1        highr_0.5.1          plyr_1.8.3
##  [5] class_7.3-14         tools_3.2.2          digest_0.6.9         annotate_1.48.0
##  [9] RSQLite_1.0.0        evaluate_0.8         gtable_0.2.0         graph_1.48.0
## [13] DBI_0.3.1            stringr_1.0.0        S4Vectors_0.8.0      IRanges_2.4.1
## [17] stats4_3.2.2         grid_3.2.2           GSEABase_1.32.0      Biobase_2.30.0
## [21] AnnotationDbi_1.32.0 XML_3.98-1.3         magrittr_1.5         scales_0.4.0
## [25] BiocGenerics_0.16.0  splines_3.2.2        BiocStyle_1.8.0      xtable_1.8-0
## [29] colorspace_1.2-6     labeling_0.3         stringi_1.0-1        munsell_0.4.3
```
