# Supplementary material for: Master Regulators of Oncogenic KRAS Response in Pancreatic Cancer: An Integrative Network Biology Analysis
Source: PLoS Med. 2017 Jan 31;14(1):e1002223. doi: 10.1371/journal.pmed.1002223 (PMC5283690; doi:10.1371/journal.pmed.1002223)
Supplement: S1 STROBE Checklist — (DOC) [file pmed.1002223.s013.doc]

STROBE Statement—Checklist of items that should be included in reports of ***cohort studies***

|  | Item No | Recommendation |
| --- | --- | --- |
| **Title and abstract** | 1 | (*a*) Indicate the study’s design with a commonly used term in the title or the abstract  We described this study as a network biology approach using 560 cases from 7 previous studies in title and abstract. |
|  |  | (*b*) Provide in the abstract an informative and balanced summary of what was done and what was found  Abstract |
| Introduction | | |
| Background/rationale | 2 | Explain the scientific background and rationale for the investigation being reported  Introduction section, paragraphs 1+2 |
| Objectives | 3 | State specific objectives, including any prespecified hypotheses  Introduction section, paragraph 3 |
| Methods | | |
| Study design | 4 | Present key elements of study design early in the paper  Methods, paragraph 1 |
| Setting | 5 | Describe the setting, locations, and relevant dates, including periods of recruitment, exposure, follow-up, and data collection  We used multiple publically available data sets where this was described in their original manuscripts that were referenced in our manuscript. |
| Participants | 6 | (*a*) Give the eligibility criteria, and the sources and methods of selection of participants. Describe methods of follow-up  Not relevant for our study- we used all available transcriptomic datasets |
|  |  | (*b*) For matched studies, give matching criteria and number of exposed and unexposed  Not relevant for our study |
| Variables | 7 | Clearly define all outcomes, exposures, predictors, potential confounders, and effect modifiers. Give diagnostic criteria, if applicable  We use the definitions described in the publically available resources. |
| Data sources/ measurement | 8* | For each variable of interest, give sources of data and details of methods of assessment (measurement). Describe comparability of assessment methods if there is more than one group  Not relevant to our study |
| Bias | 9 | Describe any efforts to address potential sources of bias  There are potential sources of bias if clinical data was not properly annotated at the time of download. We used co-variates in our statistical tests for survival data analysis to try to correct for potential bias. Methods section, paragraph on Survival Analysis. |
| Study size | 10 | Explain how the study size was arrived at  We collected all available pancreatic cancer transcriptomic datasets that had a reasonable size for network reconstruction (all but one bigger than 25 samples). |
| Quantitative variables | 11 | Explain how quantitative variables were handled in the analyses. If applicable, describe which groupings were chosen and why  S1 Computational Analysis includes a reproducible script of how all variables were handled. |
| Statistical methods | 12 | (*a*) Describe all statistical methods, including those used to control for confounding  Methods section: part 1 for network reconstruction, part 2 for Master Regulator Analysis, part 3 for downstream analysis including survival. |
|  |  | (*b*) Describe any methods used to examine subgroups and interactions  Not relevant to our study |
|  |  | (*c*) Explain how missing data were addressed  Not relevant to our study |
|  |  | (*d*) If applicable, explain how loss to follow-up was addressed  Not relevant to our study |
|  |  | (*e*) Describe any sensitivity analyses  Not relevant to our study |
| Results | | |
| Participants | 13* | (a) Report numbers of individuals at each stage of study—eg numbers potentially eligible, examined for eligibility, confirmed eligible, included in the study, completing follow-up, and analysed  We used published data and did not recruit participants ourselves. |
|  |  | (b) Give reasons for non-participation at each stage  Not relevant to our study |
|  |  | (c) Consider use of a flow diagram  Not relevant to our study |
| Descriptive data | 14* | (a) Give characteristics of study participants (eg demographic, clinical, social) and information on exposures and potential confounders  S2 Table |
|  |  | (b) Indicate number of participants with missing data for each variable of interest  Clinical data on KRAS status was only available for 420 out of 560 cases. |
|  |  | (c) Summarise follow-up time (eg, average and total amount)  Not relevant to our study |
| Outcome data | 15* | Report numbers of outcome events or summary measures over time  S1 Computational Analysis summarizes all clinical information. |
| Main results | 16 | (*a*) Give unadjusted estimates and, if applicable, confounder-adjusted estimates and their precision (eg, 95% confidence interval). Make clear which confounders were adjusted for and why they were included  Main result was identification of subtypes. Results section, section 2. |
|  |  | (*b*) Report category boundaries when continuous variables were categorized  Not relevant to our study |
|  |  | (*c*) If relevant, consider translating estimates of relative risk into absolute risk for a meaningful time period  Not relevant to our study |
| Other analyses | 17 | Report other analyses done—eg analyses of subgroups and interactions, and sensitivity analyses  Downstream analysis of subtypes included mutational load and survival analysis (Results section 3) and immune activity (Results section 4) |
| Discussion | | |
| Key results | 18 | Summarise key results with reference to study objectives  Discussion paragraph 1 |
| Limitations | 19 | Discuss limitations of the study, taking into account sources of potential bias or imprecision. Discuss both direction and magnitude of any potential bias  Discussion paragraph 2 |
| Interpretation | 20 | Give a cautious overall interpretation of results considering objectives, limitations, multiplicity of analyses, results from similar studies, and other relevant evidence  Discussion paragraph 3+4 |
| Generalisability | 21 | Discuss the generalisability (external validity) of the study results  Discussion paragraph 3+4 |
| Other information | | |
| Funding | 22 | Give the source of funding and the role of the funders for the present study and, if applicable, for the original study on which the present article is based  IdS, FM were funded by Cancer Research UK core grant C14303/A17197 and A19274  (to FM). LC was supported by the Cancer Research UK and Engineering and Physical  Sciences Research Council Imaging Centre in Cambridge and Manchester,  grant C197/A16465 (to FM). Funder's website: www.cancerresearchuk.org/ and [www.epsrc.ac.uk/](http://www.epsrc.ac.uk/). The funders had no role in study design, data collection and analysis, decision to publish, or preparation of the manuscript. |

*Give information separately for exposed and unexposed groups.

**Note:** An Explanation and Elaboration article discusses each checklist item and gives methodological background and published examples of transparent reporting. The STROBE checklist is best used in conjunction with this article (freely available on the Web sites of PLoS Medicine at http://www.plosmedicine.org/, Annals of Internal Medicine at http://www.annals.org/, and Epidemiology at http://www.epidem.com/). Information on the STROBE Initiative is available at http://www.strobe-statement.org.
