# Supplementary material for: Master Regulators of Oncogenic KRAS Response in Pancreatic Cancer: An Integrative Network Biology Analysis
Source: PLoS Med. 2017 Jan 31;14(1):e1002223. doi: 10.1371/journal.pmed.1002223 (PMC5283690; doi:10.1371/journal.pmed.1002223)
Supplement: S2 Table — Data are represented as mean (SD), median (OQR), n (%) or n. (PDF) [file pmed.1002223.s015.pdf]

		ICGC	*TCGA*
Number of patients		242	178
Age at diagnosis (years)		67 (10.6)	65 (11)
Gender	Female	110 (45%)	80 (45%)
	Male	132 (55%)	97 (55%)
Survival	Data available	240 (99%)	177 (99%)
	Overall Surival (months)	15 (9 to 25)	15 (9 to 22)
Vital Status	Alive	94 (39%)	85 (48%)
	Dead	146 (61%)	92 (52%)
Stage		IA/IB: 3 (1%)	IA/IB: 21 (12%)
		T2: 3 (1%)	IIA: 28 (16%)
		T3: 4 (2%)	IIB: 118 (67%)
		Unknown: 230 (96%)	III/IV: 4 (2%)
			IV: 4 (2%)
			Unknown: 2 (1%)
Radiation Treatment	Unknown	-	32 (18%)
	Yes	-	101 (57%)
	No	-	44 (25%)
Targeted Therapy	Unknown	-	89 (50%)
	Yes	-	46 (26%)
	No	-	42 (24%)
Disease Processes	Cell Cycle	90 (38%)	73 (41%)
	Hedgehog/Wnt	37 (15%)	45 (25%)
	Notch	113 (47%)	59 (33%)

Table S2: Summary of clinical features in the ICGC and TCGA cohorts. Data are mean (SD), median (OQR), n (%) or n.
